# Minimizing reference bias with an imputed personalized reference

**DOI:** 10.1101/gr.280989.125

**Published:** 2026-04

**Authors:** Kavya Vaddadi, Mao-Jan Lin, Sina Majidian, Taher Mun, Ben Langmead

**Affiliations:** Department of Computer Science, Whiting School of Engineering, Johns Hopkins University, Baltimore, Maryland 21218, USA

## Abstract

Pangenome indexes reduce reference bias in sequencing data analysis. However, bias can be reduced further by using a personalized reference, for example, a diploid human reference constructed to match a donor individual's alleles. Here, we present a new impute-first alignment framework that combines elements of genotype imputation and read alignment. We first genotype the individual using a subsample of the input reads. Using a reference panel and an efficient imputation algorithm, we impute a personalized diploid reference. Finally, we index the personalized reference and apply a read aligner (either linear or graph) to align the full read set to the personalized reference. On the HG002 sample, this framework achieves a higher variant-calling F1-score (99.77%) compared with the traditional linear aligner (99.62%), graph pangenome aligner (99.72%), and graph personalized-pangenome aligner (99.75%), with substantial reduction in the number of errors (38.73% vs. a linear aligner, 14.97% vs. a graph aligner, and 6.05% vs. a personalized graph). An imputed reference can have comparable efficiency to a pangenome reference, making it an overall advantageous choice for whole-genome DNA sequencing experiments. Advantages of our impute-first approach include that (1) it fully considers linkage disequilibrium and produces a phased diploid reference as an output; (2) it produces accurate personalized references even from low-coverage data; and (3) it is compatible with both graph and linear reference representations, achieving its highest variant-calling F1 accuracy using a standard linear aligner (BWA-MEM).

A goal of pangenome methods is to reduce the “reference bias” that results when aligning reads to a single reference genome (Lin et al. 2024). Bias occurs when reads containing nonreference alleles fail to align to their true point of origin, leading to inaccurate results for analyses concerned with hypervariable regions (Brandt et al. 2015), allele-specific effects (Degner et al. 2009; Rozowsky et al. 2011; Van De Geijn et al. 2015; Salavati et al. 2019), ancient DNA (Günther and Nettelblad 2019; Martiniano et al. 2020), or epigenomics (Groza et al. 2020). Pangenome alignment methods reduce bias by using a pangenome graph (Siren et al. 2014; Kim et al. 2019; Sirén et al. 2021) or a collection of linear genomes (Kuhnle et al. 2020; Ahmed et al. 2023; Zakeri et al. 2024) rather than a single reference. The aligner can thereby remove unwanted penalties associated with known genetic variants.

Although these methods reduce bias (Garrison et al. 2018; Pritt et al. 2018; Chen et al. 2021), personalized references, which are tailored to include the specific alleles present in the individual under study (the “donor”), are the most effective at reducing bias (Pritt et al. 2018; Chen et al. 2021). We present an efficient and practical “impute-first alignment” framework that combines advantages of genotype imputation and pangenome alignment. Recent imputation tools are efficient (Browning et al. 2018; Rubinacci et al. 2021, 2023) and can leverage comprehensive genetic and linkage-disequilibrium information from reference panels like The 1000 Genomes Project (1 KGP) (The 1000 Genomes Project Consortium et al. 2015) and Structural Variation Consortium (HGSVC) (Ebert et al. 2021; Logsdon et al. 2025). Because these panels are phased, they enable us to compute phased, diploid haplotypes, fully leveraging the genetic variation and linkage disequilibrium information in reference panels.

We begin by subsampling the input reads and performing genotyping and imputation steps. The output is a diploid personalized reference. We then index the personalized reference and align all the input reads to the personalized index. Here we can use one of two alignment methods. We can use a graph pangenome aligner, in which the graph encodes the imputed alleles using diverging paths for heterozygous (HET) variants. Alternately, we can use a typical linear aligner like Bowtie 2 (Langmead and Salzberg 2012) or BWA-MEM (Li 2013) but align to the personalized haplotypes rather than to the typical reference.

Past methods used personalized references to alleviate reference bias for RNA-seq (Rozowsky et al. 2011; Yuan and Qin 2012; Liu et al. 2018), with the user providing the donor's genotypes as input. Some methods additionally perform a personalization step but have other drawbacks. Gramtools (Letcher et al. 2021) builds a pangenome that can be used both to align reads and to perform imputation of a personalized genome. That study was limited to monoploids and did not attempt human scale. The iCORN method (Otto et al. 2010) gradually refines the reference to contain more alternate (ALT) alleles through an expensive iterative alignment process, also focusing on monoploids. MMSEQ (Turro et al. 2011) performs variant calling on an input BAM file to construct a personalized diploid transcriptome but does not perform imputation. RefEditor (Yuan et al. 2015) uses genotype imputation to obtain phased diploid genotypes based on SNP genotyping array data. In contrast, our work performs personalization based on the input (sequencing) data set itself and does not require a second data set. Importantly, none of the above tools use efficient imputation methods like Glimpse (Rubinacci et al. 2021), which uses the positional Burrows–Wheeler transform (PBWT) (Durbin 2014) as its computational engine. Further, none of the above-mentioned studies assessed the computational cost, and thus the practicality, of the workflow.

Groza et al. (2020) introduced Graph Personal Genome, or a graph-shaped reference containing only the donor's diploid variants, as called from a separate data set. Using this reference enables less-biased ChIP-seq peak calling. Our framework also uses this idea, but we both impute the diploid variants and create this personalized reference as part of the same data analysis. Sirén et al. (2024) introduced a personalization facility to VG (Sirén et al. 2021). The method begins with an inclusive pangenome and then uses *k*-mers derived from the input reads to personalize the reference chunk by chunk. This has the drawbacks that (1) it requires *k*-mer counting over the entire input reads set; (2) it produces a personalized pangenome that can be used together with VG Giraffe but does not provide a personalized reference that would be compatible with other nongraph tools; and (3) its personalization process is based on a *k*-mer heuristic rather than on an imputation process that can leverage the full-fidelity linkage disequilibrium information inherent in an imputation panel.

Our impute-first framework constructs phased diploid personalized references by combining read-based genotyping, imputation, and diploid reference construction, followed by alignment using either graph-based or nongraph methods. We evaluate its impact on reference bias, variant-calling accuracy, and computational performance across multiple samples, sequencing coverages, and reference panels, compared with commonly used linear and pangenome-based methods.

## Results

### Overview of impute-first alignment workflow and personalization

**Figure 1.**
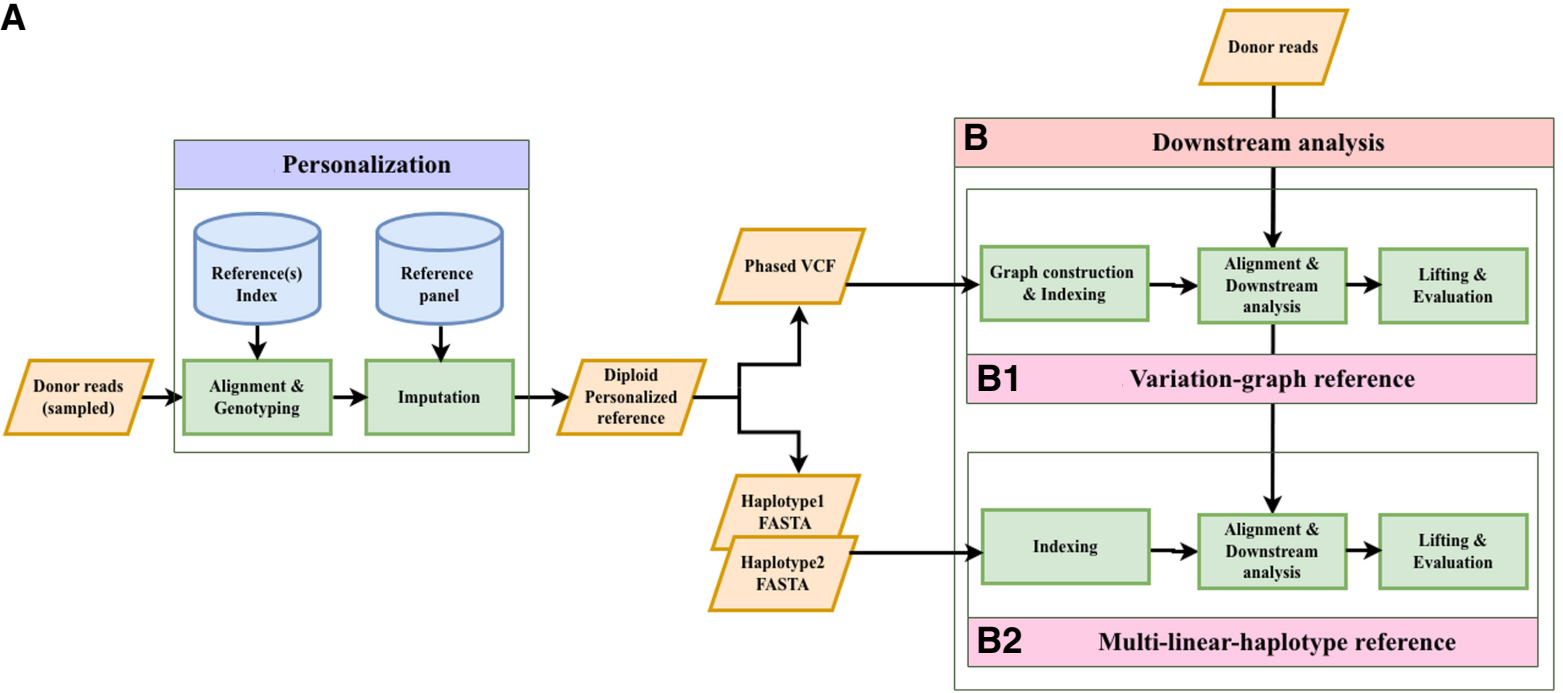
Impute-first alignment workflow in the context of analyzing a human whole-genome sequencing data set. (*A*) The workflow up until it creates a personalized diploid reference. This is the “personalization” component. (*B*) Continues the workflow by aligning the full set of reads to the personalized reference. This is the “downstream” component.

The impute-first framework includes two components, illustrated in Figure 1. In the first component, a sample of the input reads is analyzed using genotyping and imputation tools. The output is a set of phased, personalized variants, which are used to construct a personalized diploid reference genome. In the second component, the personalized reference is indexed, and an aligner is used to align all reads to this index. The workflow is modular; different tools can be substituted for the initial genotyping step (e.g., Bowtie 2 + BCFtools or pangenome genotypers such as Rowbowt or BayesTyper), the imputation step (e.g., Beagle or Glimpse), and the read alignment step (using linear aligners, e.g., Bowtie 2/BWA-MEM, or graph-based aligners, e.g., VG Giraffe).

We evaluated personalization methods in which the genotyping step used Bowtie 2 (Langmead and Salzberg 2012) and BCFtools (Li 2011). We evaluated its ability to efficiently call correct phased genotypes for five data sets derived from the Genome in a Bottle (GIAB) samples HG001–HG005 (HG001/NA12878, HG002/NA24385, HG003/NA24149, HG004/NA24143, and HG005/NA24631), which were sequenced as part of the Google Brain project (Baid et al. 2020). The initial sequencing depth for all samples were ∼30 ×, which we subsampled to lower coverage levels ranging from 0.01 × to 20 ×, respectively (Table 1). We evaluate the quality of the results from the genotyper alone and from the subsequent imputation step that uses the genotypers calls as input.

**Table 1.** The steps, inputs, outputs, and tools used in the tested impute-first workflows

Step	Input	Tool	Output
A. Personalization			
Read sampling	Donor reads: whole-genome DNA-seq reads (Baid et al. 2020) from HG001/NA12878, HG002/NA24385, HG003/NA24149, HG004/NA24143, and HG005/NA24631	seqtk (https://github.com/lh3/seqtk)	Reads sampled to 0.01×, 0.05×, 0.1×, 0.2×, 0.5×, 1×, 2×, 5×, 10×, and 20× average coverage
Alignment and genotyping	Genotype panels: HGSVC2 (Ebert et al. 2021), HGSVC3 (Logsdon et al. 2025), HPRC_filtered VCF (Ebler 2022), excluding respective samples and family members; reference: GRCh38 primary assembly (Church et al. 2015); reads: output from sampling step	Bowtie 2 (Langmead and Salzberg 2012) + BCFtools (Li 2011)	Rough genotype calls in VCF format
Imputation	Imputation panel and reference: same as previous step; genotype calls: output from genotyping step	Beagle (v5.1) (Browning et al. 2018); Glimpse (v1.0.0) (Rubinacci et al. 2021)	Personalized reference as phased VCF file
Personalized reference construction	Personalized reference: from imputation step; reference: GRCh38 primary assembly	BCFtools (bcftools consensus)	Personalized reference as diploid FASTA
B. Downstream analysis			
B.1. Variation-graph reference			
Graph construction and Indexing	Personalized reference as phased VCF file: from construction step; reference: GRCh38 primary assembly	vg (v1.55.0) autoindex (Garrison et al. 2018)	Indexed graph reference
Alignment and Lifting	Donor reads; graph reference: from previous step	vg (v1.55.0) surject (Sirén et al. 2021)	Aligned reads
Variant calling and Evaluation	Aligned reads: from previous step; true variants: HG001, HG002, HG003, HG004, and HG005 VCF from GIAB (Zook et al. 2016) high-confidence region annotations, etc.	DeepVariant v1.5.0 (Poplin et al. 2018); hap.py v0.3.15 (The Global Alliance for Genomics and Health Benchmarking Team et al. 2019)	Variant calls as VCF; benchmarking metrics
B.2. Multi-linear-haplotype reference			
Indexing	Personalized reference: From construction step; T2T-CHM13v1.0 genome assembly (Nurk et al. 2022)	bwa index (Li 2013)	Indexed reference
Alignment and Lifting	Donor reads HG001/NA12878, HG002/NA24385, HG003/NA24149, HG004/NA24143, and HG005/NA24631; indexed reference: from previous step	bwa mem (Li 2013) and levioSAM2 lift and levioSAM2 reconcile (Chen et al. 2024)	Aligned reads
Variant calling and Evaluation	Aligned reads: from previous step; true variants: HG001, HG002, HG003, HG004, and HG005 VCF from GIAB high-confidence region annotations, etc.	DeepVariant v1.5.0; hap.py v0.3.15	Variant calls as VCF; benchmarking metrics

As our accuracy measures, we measured the workflows’ ability to correctly call ALT alleles and HET variants. To measure the success of phasing, we measured “window accuracy,” namely, the fraction of 200 bp windows in the imputed diploid genome that exactly match the same window in the true genome. Ground-truth genotypes for HG002 were taken from the HGSVC2 reference panel (Chaisson et al. 2019; Ebert et al. 2021), with HG002 family members removed to prevent bias. We evaluated the workflow using reads from HG002/NA24385, subsampled to coverage levels ranging from 0.01× to 20×.

#### Genotyped and imputed call accuracy

**Figure 2.**
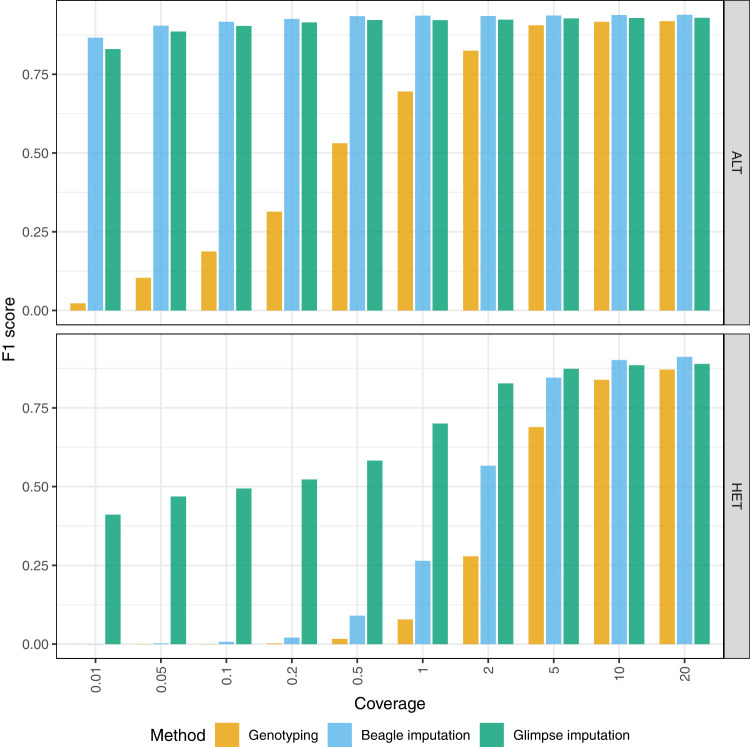
Aggregate F1-scores of alternate allele calls (ALT) and heterozygous calls (HET) across all variant types, generated using each alignment/genotyping method in the impute-first alignment workflow compared with ground-truth HG002.

We assessed genotype accuracy across various coverages both before and after imputation. We measured precision, recall, and F1-score, namely, the harmonic mean of precision and recall. For HG002, increased coverage led to more accurate calls (Fig. 2; Supplemental Fig. S1). Both Beagle and Glimpse significantly boosted the genotype call accuracies after imputation. Imputation led to notable improvements in F1 for both ALT and HET calls. For single-nucleotide variants (SNVs), both imputation methods performed comparably across all coverage levels, with each achieving F1-scores above 0.9 even at low coverage. For indel variants, a similar pattern was observed, although Beagle showed slightly higher F1-scores at higher coverages (Supplemental Fig. S1). The most striking differences appeared in structural variant (SV) calling, in which preimputation methods failed to detect SVs, whereas Glimpse demonstrated remarkable recovery of SV calls even at 0.01 × coverage with F1-scores of approximately 0.3, compared with Beagle, which showed minimal SV recovery below 0.5× coverage. At higher coverages (≥5 ×), both methods achieved comparable SV detection performance. In most scenarios, Glimpse produced more accurate genotypes at ultralow coverages, particularly for SV variants, compared with Beagle. Overall, Glimpse excels at recovering variants at low coverage, particularly for HET variants, compared with Beagle. A notable shift was observed at higher coverages (≥5×), at which Beagle achieved higher F1-scores across all variant types, when including both ALT and HET variants.

#### Window accuracy

Window accuracy is a measure of how frequently a window of genomic bases covering one or more polymorphic sites is correctly inferred in the personalized reference. We considered each of the 18.8 million sites that were polymorphic according to the HGSVC2 reference panel. We call the site under consideration the “pivot.” As a group, we consider the pivot as well as all other polymorphic sites to the left of and within 200 bp of the pivot. For each such group, we determined whether all variant calls for all variants in the group were correctly genotyped and correctly phased. This is described and illustrated further in Supplemental Figure S11.

**Figure 3.**
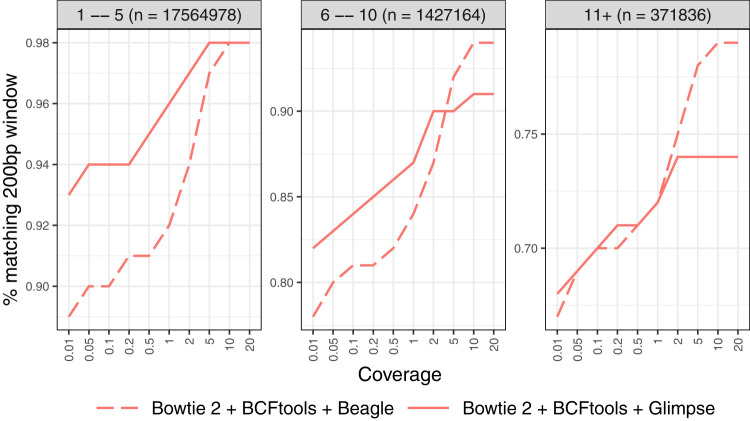
Window accuracy for diploid personalized genomes. The imputed sequence for each 200 bp window anchored to a polymorphic site was compared to the ground-truth HG002 sequence. Results are stratified by the number of polymorphic sites in the window.

Figure 3 presents the window accuracy results for HG002, with windows stratified according to the total number of polymorphic sites falling inside. The “1–5” stratum (n = 17,564,978) includes groups with one to five polymorphic sites; the “6–10” stratum (n = 1,427,164) includes groups with six to 10 polymorphic sites; and the “11+” stratum (n = 371,836) includes groups with 11 or more polymorphic sites.

For HG002 with the Bowtie 2 + BCFtools genotyper, both imputation methods demonstrated high window accuracy across all coverage levels. For the sparsest windows (“1–5” stratum), Glimpse achieved superior accuracy at lower coverages (0.93 at 0.01× vs. 0.89 for Beagle). However, as coverage increased to 5× and above, both methods converged to nearly identical performance (0.97–0.98). In the medium-density windows (“6–10” stratum), a similar pattern was observed, with Glimpse maintaining an advantage at lower coverages (0.82 vs. 0.78 at 0.01×) whereas Beagle eventually reached comparable performance at higher coverages (0.94 at 20 × vs. 0.91 for Glimpse). For the densest windows (“11+” stratum), both methods performed similarly across all coverage levels, with modest improvement as coverage increased (from approximately 0.67–0.68 at 0.01× to 0.74–0.79 at 20×). Additionally, we note that substituting BWA-MEM for Bowtie 2 in the personalization step yielded similar window accuracy, indicating that the upstream aligner choice has only a modest impact (Supplemental Table S2). As expected, window accuracy increases with average coverage because more total evidence leads to more accurate genotyping calls. Also as expected, window accuracy decreases as we consider denser strata (11+ being the densest), reflecting the increased difficulty of correctly calling all phased genotypes for many nearby variants. Glimpse excels at maintaining high window accuracy at lower coverages, whereas Beagle performs better or comparably at higher coverages, consistent with our observations in the genotype call accuracy evaluation.

Although Glimpse ran twice as fast and in ∼1% of the memory needed by Beagle (Supplemental Fig. S2), Beagle's steadily increasing accuracy at high coverages prompted us to use it in our subsequent evaluations.

#### Evaluation across multiple reference panels and samples

We next evaluated how the accuracy of the personalized reference changes when we vary the imputation panel used.

We focused on HG002, the Bowtie 2 + BCFtools genotyper and the Beagle imputation method (hereafter referred to as BBBC, in which BBBC*n* denotes the Bowtie 2 + BCFtools + Beagle pipeline evaluated at *n* × coverage and is equivalently referred to as Imputefirst_c*n* in downstream analyses, e.g., BBBC5/Imputefirst_c5 for 5× and BBBC20/Imputefirst_c20 for 20×). We evaluated coverage levels of 5×, 20 ×, and 30× (where 30× represents the original full coverage of the sequencing data). We used vcfeval engine within the hap.py to assess each phased imputed call set against the GIAB HG002 ground-truth call set. We excluded HG002 and any family members from all reference panels, if present, because including them would lead to unrealistically accurate results.

We compared three imputation panels: HGSVC2 (70 haplotypes, 18.8 million variants), HGSVC3 (130 haplotypes, 27.0 million variants), and HPRC_filtered (27.5 million variants), a biallelic imputation panel derived from the PanGenie-preprocessed HPRC v1.0 Minigraph-Cactus variant set, which includes upstream filtering of large bubbles, removal of records with >20% missing alleles, and decomposition of nested alternative alleles into single-ID records, followed by CHM13-haplotype removal, trimming of ALT alleles, and retaining only variants with at least one observed ALT allele (https://zenodo.org/record/6796991).

**Figure 4.**
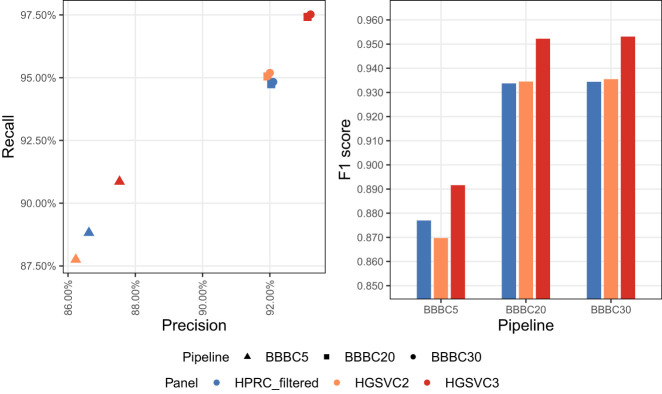
Reference panel accuracy comparison on HG002 sample. BBBC5, BBBC20, and BBBC30 denote the Bowtie 2(B) + BCFtools(B) + Beagle(B) pipeline evaluated at 5×, 20×, and 30× coverage (C), respectively.

The HGSVC3 panel yielded the highest F1-scores across all coverage levels, achieving 0.89 at 5× and 0.95 at both 20× and 30 × (Fig. 4). HGSVC3 showed higher recall (0.91–0.97) compared with other panels, while maintaining high precision (0.88–0.93). Notably, the performance at 20× and 30× was nearly identical, suggesting that beyond 20× coverage suffices in this scenario.

Given these results, we selected HGSVC3 for evaluation across four additional samples (HG001, HG003, HG004, and HG005). When evaluated with respect to the GIAB ground-truth call sets, all samples showed similar performance (Supplemental Fig. S3; Supplemental Table S1). F1-scores at 5× coverage ranged from 0.89–0.90, increasing to 0.95 at 20× and 30×. Consistent with our observations for HG002, the accuracy of imputed variant calls showed minimal improvement between 20× and 30× coverage for all samples.

#### Choices for downstream analysis

Based on all the above evaluations, we selected two configurations for our downstream analyses, which we call Imputefirst_c5 (BBBC5) and Imputefirst_c20 (BBBC20). These use Bowtie 2 + BCFtools-Beagle with the HGSVC3 reference panel, using 5× and 20× coverage, respectively, in the personalization phase. We chose two coverage levels to highlight a trade-off available to users: Using lower coverage (5×) reduces the computational overhead of the personalization step, whereas using higher coverage (20×) provides near-optimal accuracy at the cost of more personalization overhead.

### Impact of personalized references on downstream results

Having produced personalized references for HG001–HG005 with the Imputefirst_c5 and Imputefirst_c20 workflows, we next evaluated their impact on a downstream analysis consisting of read alignment and variant calling. We compared our workflows to standard linear and graph-pangenome workflows, as well as a pangenome-based personalized workflow. We evaluated both a graph-based alignment approach (VG Giraffe) and a linear alignment approach (BWA-MEM with LevioSAM2).

We evaluated in two ways. First, we examined the read alignments to the personalized index and measured allelic balance at all the HET sites. This serves as a direct measure of reference bias. Second, we used DeepVariant to call variants from the alignments to the personalized reference and then measured accuracy compared with the GIAB ground-truth call set.

#### Allelic balance at HETs

**Figure 5.**
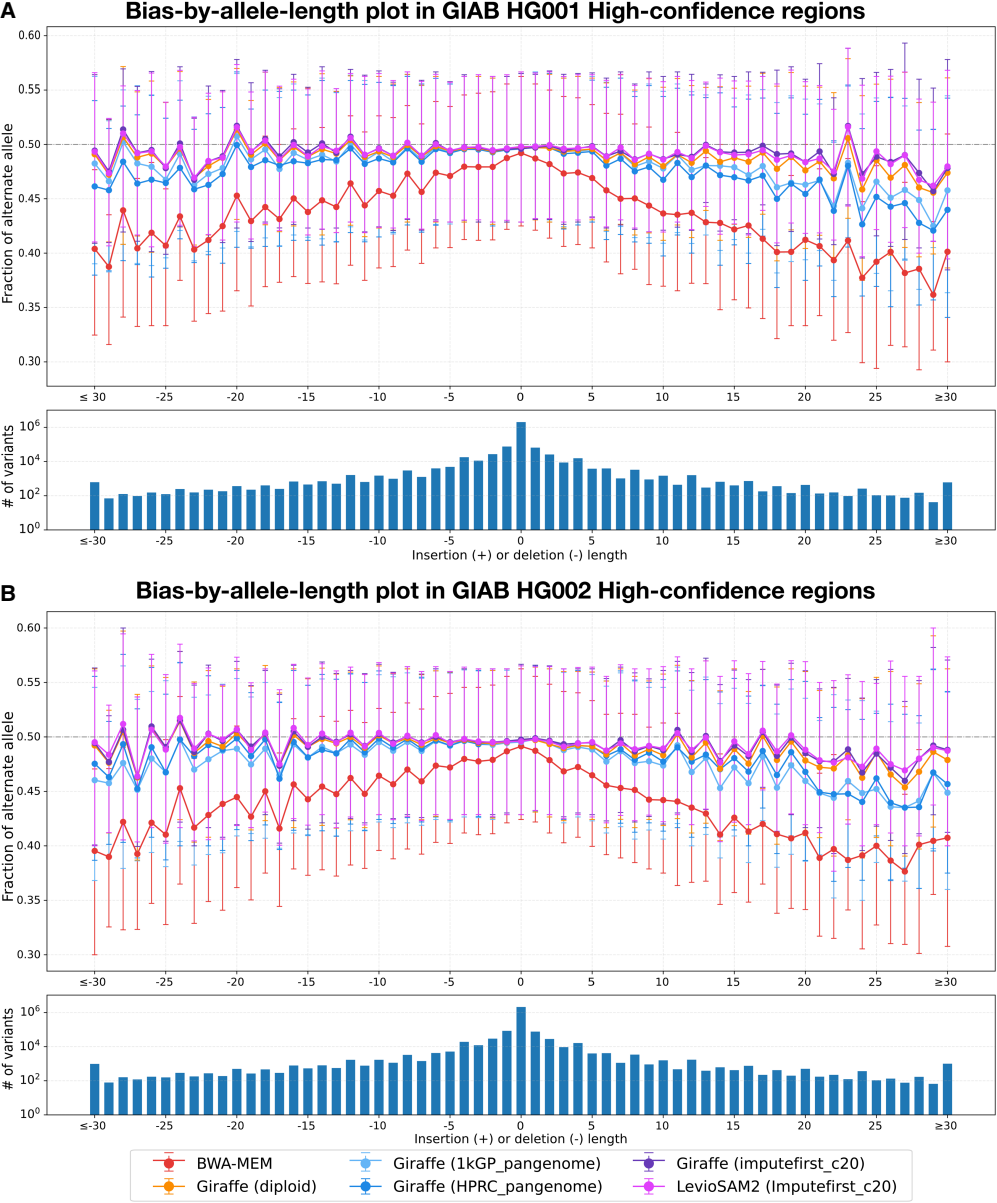
Bias-by-allele-length plots on HG001 and HG002 donor reads generated for different workflows under analysis. Variants are categorized by length: positive values for insertions, negative for deletions, and zero for SNVs at HET sites across the genome. (*A*) Allelic balance for indels and SNVs in GIAB HG001 high-confidence regions. (*B*) Allelic balance for indels and SNVs in GIAB HG002 high-confidence regions.

We identified HET variants using the GIAB truth VCF files for HG001 and HG002 and then used Biastools (Lin et al. 2024) to measure the allelic balance at these sites. We created bias-by-allele-length plots considering only the GIAB high-confidence regions for each sample (Fig. 5). The alignments obtained using personalized references consistently achieved more even (closer to 50%:50%) allelic balance at HET sites compared with other approaches. Our Imputefirst_c20 workflow demonstrated less reference bias than both the pangenome-based approaches and the linear reference, with the linear reference (BWA-MEM) exhibiting the most imbalance. This is particularly evident for the longer indels pictured on the left and right extremes of Figure 5. The LevioSAM2-based impute-first approach achieved the most balanced allelic representation (closest to 50%:50%) across all variant lengths. Both Giraffe and LevioSAM2 implementations of our Imputefirst_c20 approach significantly reduced reference bias compared with the standard references, demonstrating the effectiveness of our imputation-based personalization strategy regardless of the downstream alignment method used.

#### Variant-calling accuracy

**Figure 6.**
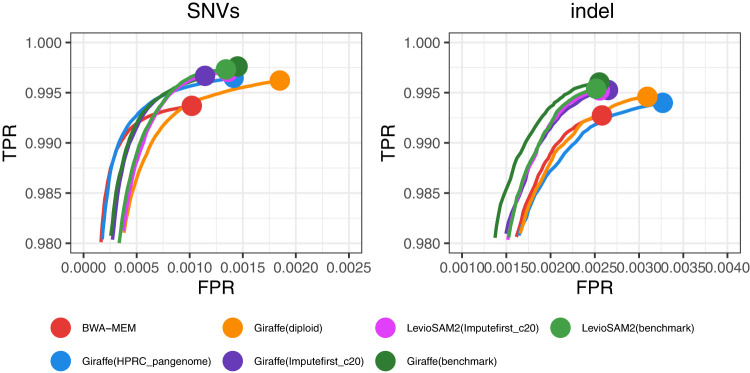
ROC curves for overall variants of HG002 sample, from Hap.py ROC data stratified on SNVs and indel variants. ROC curves are stratified by the default QQ threshold for ROC.

We evaluated variant-calling accuracy using Hap.py (v0.3.15) with the vcfeval engine against GIAB v4.2.1 truth sets for samples HG001–HG005, restricting analysis to GIAB-defined high-confidence regions. For each workflow, variants were called with DeepVariant v1.5.0 on alignments lifted to GRCh38 coordinates. Figure 6 illustrates the tradeoff between true and false positive rates for HG002.

**Figure 7.**
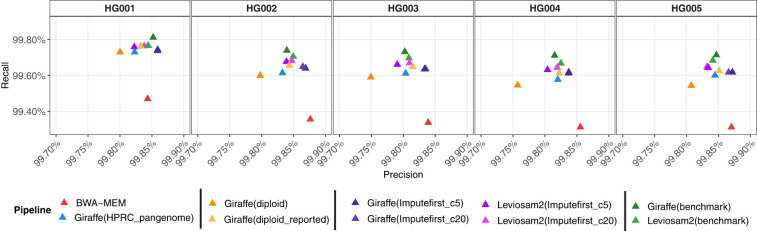
Precision-recall measures for overall variants of HG001–HG005 samples. “Giraffe” refers to the use of the standard VG Giraffe aligner. Workflows are indicated within the parentheses following “Giraffe” and “LevioSAM2.” The terms Imputefirst_c5 and Imputefirst_c20 in the parentheses indicate the chosen downstream analysis workflows of BBBC5 and BBBC20 for the sample. Benchmark in the parentheses refers to the workflow when run using the GIAB truth VCF file for the sample.

The impute-first workflows consistently achieved higher variant-calling F1-scores compared with other approaches across all five GIAB samples (Fig. 7; Supplemental Tables S5–S9). The impute-first methods reduced the number of variant-calling errors by 35.5%–41.9% relative to BWA-MEM, 6.1%–15.0% compared with pangenome approaches, and 1%–6% compared with a personalized-pangenome method. Of the impute-first methods, the LevioSAM2-based method tended to exhibit the highest F1-score.

To assess how closely our framework approaches the maximum achievable personalization performance, we compared it against an ideal benchmark configuration that constructs personalized references directly from GIAB v4.2.1 ground-truth call sets for each respective sample. We reasoned that because the GIAB call sets contain phased haplotypes constructed from high-quality sequencing data, including long reads, they can be considered as “ideal” references approaching the best that could be imputed from a large panel. Across all five GIAB samples, our impute-first pipelines consistently achieved the highest accuracy with the lowest total errors and came closest to this ceiling, with values shown as F1/total errors (impute-first vs. benchmark) for each sample, in which total errors are defined as the sum of false positives (FPs) and false negatives (FNs): HG001 (0.998017/14,780 vs. 0.998320/12,533), HG002 (0.997651/18,301 vs. 0.997895/16,408), HG003 (0.997394/20,003 vs. 0.997670/17,893), HG004 (0.997317/20,721 vs. 0.997634/18,289), and HG005 (0.997446/18,853 vs. 0.99780 /16,227). Although the GIAB data sets represent ideal targets for personalization, they can still yield errors, both because there may be errors in the GIAB data and because the reads we are using to evaluate the workflow are short reads, which can have ambiguous or incorrect alignments even to a flawless reference. So, although perfect correctness cannot be attained, it is notable that the impute-first workflows are performing closer to the ideal given by the GIAB references than are the pangenome or linear-reference workflows.

For HG002, we repeated this analysis but using the T2T consortium's new Q100 benchmark (Rhie et al. 2023), which benefits from improved sequencing data and assembly methods relative to the GIAB data sets.

We again observed consistent improvements with impute-first workflows compared with BWA-MEM, VG Giraffe with linear and pangenome references, and personalized-pangenome approaches (Supplemental Table S10).

Within the GRCh38 complex medically relevant gene (CMRG) regions tailored for HG002 (Wagner et al. 2022), the impute-first workflows had slightly lower F1-scores (0.9445–0.9524) compared with the Giraffe pangenome (0.9544), chiefly because of lower precision (Supplemental Table S11). We also evaluated performance across GIAB GRCh38 v3.1 genome stratifications (Dwarshuis et al. 2024), including low mappability (LowMap), extreme GC content (GC), segmental duplications (SegDups), tandem repeats (TRs), homopolymer repeats (HP), and other difficult regions (difficult), in which our impute-first workflows consistently demonstrated improved accuracy compared with other approaches (Supplemental Table S12). We further assessed variant-calling performance outside GIAB high-confidence regions, using v3.1 stratifications for HG002, in which accuracy was more variable across workflows; notably, BWA-MEM achieved the highest F1 (0.9166), narrowly exceeding the best graph configuration Giraffe(Imputefirst_c20) (0.9147), with other pipelines close behind (Supplemental Table S13).

To isolate the effect of imputed SVs on downstream accuracy, we repeated the HG002 analysis with the HGSVC3 panel after removing all SVs ≥50 bp; the resulting comparison (Supplemental Table S14) showed that retaining SVs consistently reduced errors, with Leviosam2(Imputefirst_c20) exhibiting ∼13% fewer FPs (8120→7050). Additionally, using the HPRC_filtered panel for HG002, we compared impute-first methods to the Giraffe(diploid) workflow, which relies on the HPRC pangenome, and Leviosam2(Imputefirst_c20) (20×) continued to outperform Giraffe(diploid) in overall accuracy (Supplemental Table S14). We also evaluated lower input coverages (0.5×–2×) for the personalization step, which still yielded effective imputation and competitive downstream accuracy, highlighting that impute-first remains robust even at low coverages (Supplemental Table S14).

### Computational efficiency

The impute-first workflow includes two main steps: (1) a personalization step, which outputs the diploid personalized genome, and (2) a downstream step for variant calling (Fig. 1). We benchmarked the computational requirements of both steps, as detailed below.

#### Personalization overhead

We evaluated the computational overhead of the personalization step, which involves alignment and genotyping with Bowtie 2 + BCFtools genotyper, followed by imputation with Beagle and construction of personalized diploid FASTA sequences (Supplemental Table S3). We define the total workflow cost as the combination of personalization and downstream steps (Supplemental Table S4) and calculated the percentage incurred during personalization. For the main analyses we focused on 5× and 20× input coverages, for which the overhead of personalization accounted for ∼16% (Giraffe(Imputefirst_c5), 149 min, 69,116 CPU sec) and 44% (Giraffe(Imputefirst_c20), 405 min, 287,855 CPU sec) of total CPU time in the graph-based workflows and 5% Leviosam2(Imputefirst_c5), 149 min, 69,116 CPU sec) and 19% (Leviosam2(Imputefirst_c20), 405 min, 287,855 CPU sec) in the nongraph workflows. Although we limited downstream variant-calling analyses to 5× and 20×, we additionally examined the computational overhead at 1×, at which the overhead of personalization accounted for 4.5% of total CPU time in the graph-based workflow (Giraffe(Imputefirst_c1), 59 min, 17,552 CPU sec) and 1.3% in the nongraph workflow (Leviosam2(Imputefirst_c1), 59 min, 17,552 CPU sec). Notably, Imputefirst_c1 workflows achieved downstream variant-calling accuracies comparable to higher coverages (Supplemental Table S14): for graph-based Giraffe workflows, F1-scores were 0.9974 (1×), 0.9975 (5×), and 0.9976 (20×); for nongraph LevioSAM2 workflows, F1-scores were 0.9969 (1×), 0.9975 (5×), and 0.9976 (20×). Using a lower coverage (1× or 5×) reduces the computational overhead of personalization, whereas using higher coverage (20×) provides near-optimal accuracy at the cost of greater personalization overhead.

#### Downstream computational efficiency

We compared the runtime and memory usage for 11 downstream variant-calling workflows on HG002, encompassing four reference representations as summarized in Table 2: linear (BWA-MEM, Giraffe(linear)), pangenome (Giraffe( 1kGP_pangenome), Giraffe(HPRC_pangenome)), personalized-pangenome (Giraffe(diploid)), and impute-first personalized references evaluated at 1×, 5×, and 20× input coverages and implemented using either graph-based alignment (Giraffe(Imputefirst_c5), Giraffe(Imputefirst_c20)) or linear alignment with support for lifting coordinates (Leviosam2(Imputefirst_c5), Leviosam2(Imputefirst_c20)). The Giraffe workflows use graph-based alignment against either a linear reference, a population-scale pangenome, or a sample-specific diploid graph, whereas LevioSAM2 performs coordinate lifting and alignment on impute-first personalized diploid references. For the Giraffe(diploid) workflow, the sample-specific diploid graph is derived from the offline-constructed Giraffe index. Specifically, it selects a subsample of haplotypes according to the number of *k*-mer matches they have to the sample reads (Sirén et al. 2024). We count this computation as part of the Giraffe(diploid) workflow's per-sample personalization/indexing step.

**Table 2.** Downstream workflows assessed

Approach	Workflow	Description
BWA-MEM	BWA-MEM	Standard linear reference genome (GRCh38)
VG Giraffe	Giraffe(linear)	Standard linear reference genome (GRCh38) with no call set
	Giraffe(HPRC_pangenome)	HPRC v1.1 frequency-filtered (GRCh38) pangenome including 44 sample assemblies + CHM13 haplotype
	Giraffe(1 kGP_pangenome)	GRCh38 phase3 1 kGP call set pangenome
	Giraffe(Imputefirst_c5)	Personalized diploid reference using Bowtie 2 + BCFtools + Beagle with HGSVC3 panel at 5× coverage (BBBC5)
	Giraffe(Imputefirst_c20)	Personalized diploid reference using Bowtie 2 + BCFtools + Beagle with HGSVC3 panel at 20× coverage (BBBC20)
	Giraffe(diploid)	Personalized pangenome using Giraffe's diploid-sampling
	Giraffe(benchmark)	Diploid reference created using GIAB truth set
BWA-MEM + LevioSAM2	Leviosam2(Imputefirst_c5)	Personalized diploid reference using Bowtie 2 + BCFtools + Beagle with HGSVC3 panel at 5× coverage
	Leviosam2(Imputefirst_c20)	Personalized diploid reference using Bowtie 2 + BCFtools + Beagle with HGSVC3 panel at 20× coverage
	Leviosam2(benchmark)	Diploid reference created using GIAB truth set

For analyses using the HPRC pangenome and 1 kGP pangenome, the sample under analysis and its family members were excluded, if present. The benchmark represents results using the GIAB ground-truth call set for the respective samples, indicating the best achievable performance with each workflow. Giraffe(diploid_reported) refers to variant-calling performance statistics directly reported by Sirén et al. (2024) for the corresponding samples.

**Figure 8.**
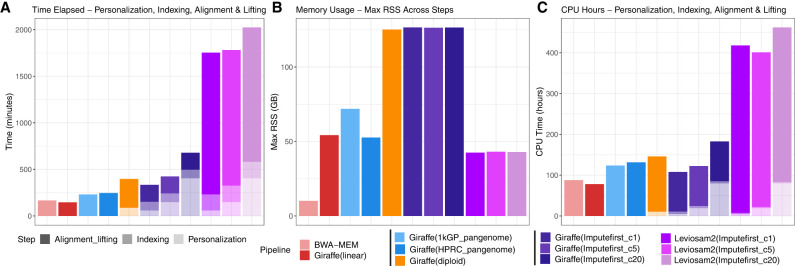
Computational efficiency of personalization, indexing, alignment, and lifting across the evaluated workflows. (*A*–*C*) Summarization of wall-clock runtime, peak memory usage, and CPU time for linear (BWA-MEM, Giraffe(linear)), pangenome (Giraffe(1 kGP_pangenome), Giraffe(HPRC_pangenome)), personalized-pangenome (Giraffe(diploid)), and impute-first strategies using either VG Giraffe (Giraffe(Imputefirst_c5), Giraffe(Imputefirst_c20)) or LevioSAM2 (Leviosam2(Imputefirst_c5), Leviosam2(Imputefirst_c20)). Bars are shaded to distinguish the types of computation. Note that only the workflow steps that depend on the input data are measured here. “Offline” index building steps that are input data independent are not counted. All workflows were executed using 32 threads, except for the BCFtools consensus and BWA-MEM indexing steps, which ran on a single thread.

Figure 8 summarizes runtime (panel A), memory usage (panel B), and CPU time (panel C). The shading of the bars distinguishes computation that can be attributed to the personalization, indexing, and alignment and lifting components of the computation. Computation in the “personalization” category is a function of the input reads; all the impute-first workflows as well as the Giraffe(diploid) include such computation. Computation in the “indexing” category relates to any index building computation that must occur after personalization, for example, the building of the final personalized Giraffe or BWA-MEM indexes in the impute-first workflows.

For the graph-based impute-first workflows, per-sample indexing required 92.88 min (20,780 CPU sec) for 1× coverage, 93.53 min (18,842 CPU sec) for 5× coverage, and 92.27 min (18,731 CPU sec) for 20 × coverage; each used 126 GB peak memory. Alignment and lifting required 181.33 min (349,694 CPU sec) for 1×, 182.09 min (352,221 CPU sec) for 5×, and 181.02 min (350,652 CPU sec) for 20×.

For the lifting-based impute-first workflows, the indexing step required 172–177 min (10,166—10,425 CPU sec) for 1×, 5×, and 20× coverage configurations. Reported indexing times exclude the one-time cost of constructing a BWA-MEM index of the T2T-CHM13 reference because this step does not depend on the input reads. Alignment and lifting required 1521.56 min (1,476,739 CPU sec) for 1×, 1455.60 min (1,364,193 CPU sec) for 5×, and 1441.91 min (1,365,112 CPU sec) for 20×. Compared to the graph-based impute-first workflows, these lifting-based workflows used substantially less memory but also more time.

The pangenome-based workflows required 230 min (444,301 CPU sec) and 71.89 GB for Giraffe(1 kGP_pangenome) and 245 min (472,920 CPU sec) and 52.63 GB for Giraffe(HPRC_pangenome).

Given these results, we see that the computational overhead of impute-first alignment is a function of the coverage used for personalization; this affects the time required for the personalization phase, whereas the indexing, alignment, and lifting phases are not strongly influenced by coverage. Although the lifting-based impute-first workflows incur high total runtime owing to the need to align separately to two personalized haplotypes, future implementations could reduce this cost with a reference-flow-like approach that strives to avoid duplicated alignment effort. Detailed stepwise and aggregate runtime, memory, and CPU usage measurements are provided in Supplemental Tables S4 and S15.

Overall, our impute-first framework can construct a diploid personalized reference at a comparable computational cost relative to approaches that use a pangenome. Further, we showed that these personalized references are accurate, having high variant-level precision and recall, as well as accurate overall phasing. Finally, we showed that a pangenome index built over a personalized genome can be as computationally efficient as a pangenome index, while being less biased and producing more accurate downstream variant calls, making the impute-first framework an appealing choice for whole-genome DNA sequencing data analyses.

## Discussion

We introduced a practical impute-first approach for genomic analysis with the goal of minimizing reference bias, even beyond what can be achieved with pangenome references. The workflow includes initial genotyping and genotype imputation steps, which produce a diploid personalized reference. This reference can then be indexed and used as a basis for downstream alignment and variant calling. The method yields improvements in all aspects measured: It yields higher alignment scores, improved allelic balance at HET sites, and enhanced downstream variant calls. VG Giraffe alignments using an impute-first personalized reference achieved greater variant-calling accuracy compared with Giraffe with a pangenome reference. Further, the Giraffe index built using the imputed personalized reference was smaller, reducing the computational burdens associated with building the index and aligning reads to it.

Although past methods have explored the use of personalized references for avoiding reference bias, this study is the first to demonstrate a practical workflow that (1) performs personalization and downstream analysis with the same data set, (2) leverages imputation tools that work with inputs derived from (possibly low-coverage) sequencing data, (3) outputs a diploid personalized reference genome for various downstream uses, and (4) works naturally with either linear or graph pangenome aligners.

Although we concentrated on certain tools here, including BWA-MEM, VG Giraffe, and Beagle, the framework we proposed is modular. For example, in our previous work, we assessed the trade-offs of alternative genotypers, including PanGenie (Ebler et al. 2022) and Rowbowt (Mun et al. 2023), noting that alignment-free, pangenome-based methods such as Rowbowt show strong promise in reducing reference bias, although at the cost of higher memory usage. Such alignment-free approaches could potentially improve results further and remain an interesting avenue for future research.

In the future, it will be important to further improve the computational steps in the workflow's personalization phase: genotyping and imputation. For instance, the recently described minimal positional substring cover (MPSC) framework (Sanaullah et al. 2023) can solve a version of the imputation problem in time that does not depend on the number of haplotypes in the imputation panel. It is not immediately applicable, however, as it does not currently handle phased diploid imputation. Further improvement could come from combining results from multiple imputation tools, as observed in ancestral DNA studies (Martiniano et al. 2020).

Although our method shows promising results with whole-genome DNA sequencing data, more work is required to establish its applicability to other assays like exome sequencing and RNA-seq. These assays exhibit uneven coverage across the genome, which complicates the initial genotyping and imputation steps. For exome sequencing, coverage is deep in exonic regions and shallow elsewhere, leading to higher genotyping and imputation accuracy in exons; accuracy will then presumably fall off in areas flanking the exons, and only low accuracy will be achieved between exons. RNA-seq poses an additional problem because variability in expression levels also complicates genotyping; lowly expressed genes may not provide sufficient data for genotyping and imputation.

Our results further show that impute-first workflows approach the maximum achievable personalization performance, as defined by constructing references directly from GIAB ground-truth haplotypes. Although these call sets represent ideal targets, they can still yield errors because of limitations in the call sets themselves and because short-read evaluation can introduce ambiguous or incorrect alignments. Thus, perfect correctness cannot be attained but impute-first workflows perform much closer to this GIAB-based ceiling than either linear or pangenome-based workflows, suggesting that more comprehensive haplotype panels could help narrow the gap further.

We opted to use the HGSVC reference panel in the personalization phase of our workflow because that panel was compiled using advanced long-read assembly methods that allowed it to survey complex SVs. However, more work is required to determine which are the most important features the imputation panel should have to minimize reference bias and maximize downstream accuracy. Although we opted for a panel with relatively few individuals but a relatively wider survey of SVs, it could also be advantageous to choose a panel with more individuals but a narrower range of SVs, such as that produced by 1 KGP. Notably, the recent Glimpse2 algorithm (Rubinacci et al. 2023) is able to handle BioBank-scale reference panels, as demonstrated using the 150,000 samples in the UK BioBank.

## Methods

Figure 1 illustrates the impute-first workflow. First, a set of reads is sampled from the full set of input reads. Next, the sampled read set is used to obtain rough genotypes for the donor. The rough genotypes are passed to an imputation tool, which imputes and phases both donor haplotypes, yielding a personalized diploid reference genome. This is the “personalization” portion of the workflow (Fig. 1A). In scenarios in which high-quality variant calls are desired, the workflow can be continued further downstream (Fig. 1B). In this case, the diploid personalized reference will be indexed and used to analyze the full input read set.

### Personalization

#### Sampling

Past work has shown that genotype imputation can be performed with excellent accuracy even when the input reads represent a low level of average coverage (Li et al. 2021; Martin et al. 2021). To study this in the context of impute-first alignment, we constructed subsampled data sets using seqtk, which subsamples randomly to achieve a configurable fraction of the original coverage. In this way, we generated read sets spanning very low (0.01 ×) to high (20×) coverage, starting from the original 30 × coverage data.

#### Genotyping

The next step obtains genotypes based on the subsampled reads. Given the subsampling, the genotypes obtained in this step may not be accurate by themselves. However, even rough genotypes can be sufficient input for a modern imputation tool, which can leverage large databases of high-quality phased genotypes to achieve accurate, phased genotype calls.

This step of the personalization workflow is modular; various tools could be inserted here to accomplish the rough genotyping task. In our experiments, we used a workflow based on Bowtie 2 + BCFtools.

The input reads are first aligned to the human GRCh38 reference genome using Bowtie 2 v2.4.2 (Langmead and Salzberg 2012) with default parameters and 32 threads. After alignment, a read pileup is generated for each genomic position using BCFtools v1.13 (BCFtools) (Danecek et al. 2021) with bcftools mpileup and using default settings. The pileup is then given as input to bcftools call (default parameters) to make rough variant calls.

To ensure the accuracy and reliability of identified variants, the low-quality attributes were filtered out by excluding VCF records with INFO/QUAL values below 20 and INFO/DP values exceeding 100. It is important to note that both pileup generation and variant-calling stages were conducted using single-thread configurations owing to the lack of multithreading support in the respective analysis modules used.

### Diploid genotype imputation

#### Beagle

We used Beagle v5.1 (Browning et al. 2018) to phase and impute genotypes generated with Bowtie 2 + BCFtools. We executed Beagle with default parameters using 32 simultaneous threads. We performed phasing and imputation on a per-chromosome basis. We evaluated three imputation reference panels: *HGSVC2* (35 samples), *HGSVC3* (65 samples), and *HPRC_filtered* (44 samples). The *HPRC_filtered* panel, used for imputation, corresponds to the original Minigraph-Cactus HPRC panel with the CHM13 reference haplotype excluded, and that was processed into a biallelic VCF using the prepare-vcf-MC workflow (https://github.com/eblerjana/genotyping-pipelines/tree/main/prepare-vcf-MC). Among the five GIAB samples (HG001–HG005), HG001 (NA12878), and HG002 (NA24385) are present in HGSVC2, whereas only HG002 is present in HGSVC3. The *HPRC_filtered* panel, used for imputation, does not include any of the five GIAB samples (HG001–HG005). To avoid imputation bias, any sample and its family members were excluded from the panel during its analysis. For HGSVC2, HG001 and HG002 were excluded when imputing those respective samples. For HGSVC3, HG002 was excluded when imputing HG002, HG003, and HG004, because these three form an Ashkenazim trio. HG001 and HG005, which are not present in HGSVC3, were analyzed using the full panel. An analysis of the relatedness of the panel individuals indicated there were no further family relations to consider (Supplemental Fig. S10). The HPRC panel was used in full across all samples. The genetic map files used for phasing and imputation were downloaded from the Beagle web resource (https://bochet.gcc.biostat.washington.edu/beagle/genetic_maps/).

#### GLIMPSE

We used Glimpse v1.0.0 (Rubinacci et al. 2021) together with the same reference panel and genetic map used in the Beagle experiments. We first converted the genetic map to Glimpse's format. We executed Glimpse with 32 simultaneous threads. We ran all of Glimpse's distinct modules—chunk, ligate, phase, and sample—with default parameters. As we did for Beagle, we conducted the imputation on a per-chromosome basis.

### Construction of personalized references and index

#### Impute-first personalized linear reference

We used bcftools consensus to construct diploid reference genomes in FASTA format for all five GIAB samples (HG001–HG005), using the phased, imputed VCFs generated by our selected configurations. For each sample and for each selected configuration, bcftools consensus generated two haplotype-specific genome sequences (Haplotype_1.fasta and Haplotype_2.fasta) by substituting ALT alleles into the GRCh38 reference. This process also produced chain files capturing coordinate shifts from GRCh38 to each personalized haplotype. These chain files were later used during variant calling to lift alignments from the personalized genome back to the GRCh38 coordinate space, ensuring compatibility with standard evaluation benchmarks. All personalized haplotypes, along with GRCh38, were indexed using bwa index with default parameters to support downstream alignment with BWA-MEM.

#### Impute-first personalized graph reference

We generated personalized reference graphs using vg autoindex ‐‐workflow Giraffe (vg version 1.55.0) for all five GIAB samples (HG001–HG005). We used default parameters and 32 simultaneous threads. As inputs, we provided both the GRCh38 reference FASTA and the phased, imputed diploid variant calls from the Imputefirst_c5 and Imputefirst_c20 VCFs for each sample. The resulting graphs each encode a single diploid individual; where there were the homozygous ALT calls, those were substituted in, and where there were HET calls, a “bubble” was created in the graph to accommodate both alleles.

#### Other references

We constructed VG Giraffe indexes for linear (without VCF), pangenome (1 kGP Phase 3 VCF), and benchmark (GIAB v4.2.1 truth VCF) variant sets. We did this using the GRCh38 reference FASTA and the vg autoindex workflow. To include HPRC pangenome-based evaluations, we used the HPRC v1.1 frequency-filtered GRCh38-based VG Giraffe indexes provided as part of the HPRC pangenome resources. To construct the VG Giraffe personalized pangenomes, we followed the VG Giraffe workflow for haplotype sampling, as described at GitHub (https://github.com/vgteam/vg_wdl/blob/master/workflows/giraffe.wdl), using the HPRC v1.1 default GRCh38-based giraffe index for sample-specific diploid sampling.

### Alignment and variant calling

#### Alignment with VG Giraffe

Using the personalized reference graph indexes created through the VG Giraffe autoindex workflow, we employed the VG Giraffe mapping module (vg giraffe) to align the full set of donor reads for all five GIAB samples (HG001–HG005). Alignments were performed using vg version 1.55.0, which infers read-specific parameters by default. The resulting alignments were surjected to GRCh38 coordinate space to produce standard BAM files for each reference combination, which were used as input for subsequent analyses. We followed the same alignment procedure for all Giraffe-based workflows.

#### Alignment with Leviosam2 + BWA-MEM

To use the personalized genomes, we first aligned the sample's reads to Haplotype_1 and Haplotype_2 separately with BWA-MEM. We then used leviosam2 lift to lift the resulting read alignment back to GRCh38 coordinates, guided by the chain files generated by bcftools consensus. In addition to the personalized genomes, we also incorporated the T2T-CHM13 reference genome (Nurk et al. 2022) into the workflow to rescue reads that misalign or fail to align owing to sequence missing from GRCh38 but present in T2T-CHM13. Reads aligned to T2T-CHM13 were similarly lifted to GRCh38 using leviosam2 lift, with a chain file generated by nf-LO (Talenti and Prendergast 2021) describing the mapping between GRCh38 and T2T-CHM13. After performing BWA-MEM alignment and LevioSAM2 lift for all three references (Haplotype_1, Haplotype_2, and T2T-CHM13), the resulting alignments were reconciled into a single GRCh38-coordinate BAM file using leviosam2 reconcile. For each read, the best alignment among the three candidate alignments was selected based on mapping quality and alignment score. This reconciled BAM file represents the final output of the BWA-MEM + LevioSAM2 workflow (Supplemental Fig. S4).

#### Variant calling with DeepVariant

We applied DeepVariant (v1.5.0) to all downstream workflows listed in Table 1, using the aligned BAM files generated from the full donor read sets for HG001–HG005. Prior to variant calling, all alignments were lifted to GRCh38 coordinates using the appropriate method from each workflow. For VG Giraffe-based workflows, we used the vg surject module to project graph-based alignments onto the linear reference. For workflows based on BWA-MEM and LevioSAM2, we used levioSAM2 lift and levioSAM2 reconcile to lift alignments from the personalized haplotypes and the T2T-CHM13 reference to GRCh38, producing a unified coordinate-consistent alignment. For workflows involving personalized references, we additionally performed local indel realignment to refine alignments around small indels. Specifically, we left-aligned indels using bamleftalign (from FreeBayes version 1.3.8) (Garrison and Marth 2012), identified realignment targets with GATK's RealignerTargetCreator (McKenna et al. 2010), extended boundaries with BEDTools slop, and then applied local realignment using ABRA (v2.19) (Mose et al. 2014), following the workflow described in previous studies (Liao et al. 2023; Sirén et al. 2024).

DeepVariant was configured with ‐‐model_type=WGS, a minimum mapping quality of one, and the flags keep_legacy_allele_ counter_behavior=true and normalize_reads=true to ensure compatibility across diverse reference contexts. This produced .vcf.gz variant call files for each sample and workflow, which were evaluated against GIAB truth sets using hap.py to measure precision, recall, and F1-score across variant types and benchmark regions.

### Evaluation

#### Measuring genotyping and imputation accuracy

We analyzed the accuracy of the personalization workflows’ diploid genotypes in two ways. First, we considered allele-by-allele precision and recall, considering the ALT allele calls from the sample-specific truth VCF to be the positive class. Specifically, every diploid genotype called by a method is considered as a pair of individual allele calls. If a given allele call is an ALT allele and there is at least one ALT allele present in the true diploid genotype at that site, it counts as a true positive (TP). If the given allele is a reference allele (REF) and there is at least one REF allele in the true diploid genotype, this is a true negative (TN). If the given allele is an ALT but the true genotype is homozygous REF, this is counted as one FP. Finally, if the given allele is REF but the true genotype is homozygous ALT, this is counted as one FN.

Second, we considered precision and recall with respect to sites that were either truly HET or called HET. If a HET call made by a method is truly HET, this was counted as a TP. FPs, FNs, and TNs are defined accordingly.

In both cases, precision and recall are computed as

$$\mathrm{Precision}=\frac{\mathrm{TP}}{\mathrm{TP}+\mathrm{FP}}\;\mathrm{Recall}=\frac{\mathrm{TP}}{\mathrm{TP}+\mathrm{FN}}.$$

#### Measuring window accuracy

Window accuracy is a measure of how frequently a window of genomic bases covering one or more polymorphic sites is correctly inferred in the personalized genome. For each polymorphic site (referred to as the “pivot”), we considered a 200 bp window containing the pivot and all other polymorphic sites to its left within 200 bp. For each such window, we determined whether all variant calls for all variants in the group were correctly genotyped and correctly phased.

We stratified windows by variant density: “1–5” for windows containing one to five polymorphic sites, “6–10” for six to 10 sites, and “11+” for windows with 11 or more sites. Window accuracy for each stratum was calculated as the fraction of windows in which all variants were correctly called and phased. This metric provides insight into how well each method can reconstruct local haplotype structure (for illustration, see Supplemental Fig. S11).

#### Measuring allelic balance at HETs

To quantify reference bias, we used biastools v0.3.0 with its context-aware assignment algorithm (Lin et al. 2024). biastools calculates allelic balance, defined as the number of ALT reads divided by the total number of ALT and REF reads mapped to HET variant sites in the benchmark VCF for each sample (HG001–HG005). To visualize the distribution of reference bias, biastools stratifies variants by their length. SNVs are considered to have a length of zero and appear in the center. Deletions appear to the left with larger deletions further left. Insertions appear to the right, larger insertions further right. Variants >30 bp are grouped into the same bin as those of length 30 (Fig. 5). We analyzed BAM files generated by six different workflows for HG001 (Fig. 5A) and HG002 (Fig. 5B) and by all workflows except the 1 kGP pangenome workflow for HG003–HG005 (Supplemental Figs. S5–S7). In the resulting plots, each bin represents variants of a specific length. For each bin, the mean allelic balance is shown as a central marker. The 25th and 75th percentiles are displayed as ticks to indicate the spread of the data. An allelic balance near 0.5 suggests minimal reference bias, whereas lower value implies a tendency to favor reference allele. The lower panels of Figure 5A and 5B show the number of variants per sample within each bin. Although longer variants tend to exhibit stronger reference bias, most variants are SNVs and short indels, in which the reference bias is less pronounced.

#### Assessing variant-call accuracy

To evaluate variant-calling accuracy, we used the hap.py tool (v0.3.15), the GA4GH-recommended benchmarking engine for haplotype-aware comparisons of small variants. For each sample, we compared the VCF output of each workflow to the corresponding GIAB truth set, restricting the evaluation to GIAB-defined high-confidence regions. The comparisons were conducted using the vcfeval engine within hap.py, which annotates variant calls as TPs, FPs, or FNs. These annotations were used to compute measures including precision, recall, and F1-score. The outputs of hap.py include a high-level summary file (.summary.csv) and an extended file (.extended.csv) containing measures stratified by variant type and region. In addition, we generated ROC curves (Fig. 6; Supplemental Fig. S9) using SNVs and indel ROC data files produced by hap.py, which summarize performance across varying QQ thresholds. These files contain variant performance metrics computed using the QUAL field from the VCF as the quality score. ROC curves were generated as plots of true-positive rate (TPR) versus false-positive rate (FPR), adapted from the original hap.py ROC plotting script (https://github.com/Illumina/hap.py/blob/master/src/R/rocplot.Rscript). Benchmarking was performed for samples HG001–HG005 using the GIAB v4.2.1 truth sets and high-confidence regions. For HG002, we additionally evaluated performance on the T2T-CHM13 Q100 truth set as well as across stratified GIAB-defined challenging regions, including LowMap, GC, SegDups, TRs, HPs, and other difficult regions (difficult) (Dwarshuis et al. 2024). We also assessed performance in the GIAB GRCh38 CMRG regions.

### Data sets

We used the GRCh38 reference genome (https://www.encodeproject.org/files/GRCh38_no_alt_analysis_set_GCA_000001405.15/) and whole-genome sequencing data for GIAB samples HG001–HG005 from https://registry.opendata.aws/google-brain-genomics-public. Full list of data sets used in this study are listed in Supplemental Table S16.

### Code availability

Scripts to reproduce the analyses and figures presented in this study are available at GitHub (https://github.com/kvaddad1/impute-first and https://github.com/kvaddad1/impute-first/tree/main/imputefirst_workflows) and as Supplemental Code.

## Supplemental Material

Supplement 1

Supplement 2
