# Supplementary material for: Minimizing reference bias with an imputed personalized reference
Source: Genome Res. 2026 Apr;36(4):740–53. doi: 10.1101/gr.280989.125 (PMC13138014; doi:10.1101/gr.280989.125)
Supplement: Supplement 1 [file Supplemental_Code.zip › imputefirst-main/plots_data_scripts/downstream_plots/downstream_plots.html]

Genomic Data Plots for Version 1.5.0


# Genomic Data Plots for Version 1.5.0

## Precision-recall plots and error analysis for genomic samples (Version 1.5.0 only)

```
library(ggplot2)
library(dplyr)
library(readr)
library(scales)
library(cowplot)
library(tidyr)
library(gridExtra)
library(grid)  # For rectGrob
library(ggpubr) # For computational overhead plots
library(forcats)
```

```
# Define pipeline colors
pipeline_colors <- c(
  # Original colors
  "BWA-MEM" = "#D32F2F", # Dark red for Giraffe linear
  
  "Giraffe(HPRC_pangenome)" = "#1E88E5",  # Blue for HPRC Pangenome
  "Giraffe(1kGP_pangenome)" = "#64B5F6",  # Lighter blue for 1kGP Pangenome

  "Giraffe(diploid)" = "#FB8C00",  # Bright Orange
  "Giraffe(diploid_reported)" = "#FFB74D",  # Lighter Orange
  
  "Giraffe(Imputefirst_c20)" = "#673AB7",  # Deep purple for Giraffe c20
  "Giraffe(Imputefirst_c5)" = "#4527A0",  # Dark purple for Giraffe c5
  
  "Leviosam2(Imputefirst_c5)" = "#AA00FF",  # Bright purple/pink for Leviosam2 c5
  "Leviosam2(Imputefirst_c20)" = "#E040FB",  # Pink/magenta for Leviosam2 c20
  
  "Giraffe(benchmark)" = "#2E7D32",  # Dark Green
  "Leviosam2(benchmark)" = "#43A047"  # Light Green
)

# Define pipeline colors
pipeline_colors_linear <- c(
  # Original colors
  "BWA-MEM" = "#EF9A9A",  # Red for BWA-MEM
  "Giraffe(linear)" = "#D32F2F", # Dark red for Giraffe linear
  
  "Giraffe(HPRC_pangenome)" = "#1E88E5",  # Blue for HPRC Pangenome
  "Giraffe(1kGP_pangenome)" = "#64B5F6",  # Lighter blue for 1kGP Pangenome

  "Giraffe(diploid)" = "#FB8C00",  # Bright Orange
  "Giraffe(diploid_reported)" = "#FFB74D",  # Lighter Orange
  
  "Giraffe(Imputefirst_c20)" = "#673AB7",  # Deep purple for Giraffe c20
  "Giraffe(Imputefirst_c5)" = "#4527A0",  # Dark purple for Giraffe c5
  
  "Leviosam2(Imputefirst_c5)" = "#AA00FF",  # Bright purple/pink for Leviosam2 c5
  "Leviosam2(Imputefirst_c20)" = "#E040FB",  # Pink/magenta for Leviosam2 c20
  
  "Giraffe(benchmark)" = "#2E7D32",  # Dark Green
  "Leviosam2(benchmark)" = "#43A047"  # Light Green
)

# Define pipeline order
pipeline_order <- c(
  "BWA-MEM",
  "Giraffe(linear)",
  "Giraffe(1kGP_pangenome)",
  "Giraffe(HPRC_pangenome)",
  "Giraffe(diploid)",
  "Giraffe(diploid_reported)",
  "Giraffe(Imputefirst_c5)",
  "Giraffe(Imputefirst_c20)",
  "Leviosam2(Imputefirst_c5)",
  "Leviosam2(Imputefirst_c20)",
  "Leviosam2(benchmark)",
  "Giraffe(benchmark)"
)
```

```
# Load the data file
data <- read_csv("overall_stats_v1.5.csv")

# Filter for version 1.5.0 only
#data <- data %>%
#  filter(Version == "1.5.0")

# Order samples properly
data$Sample <- factor(data$Sample, levels = c("HG001", "HG002", "HG003", "HG004", "HG005"))

# Remove any rows with missing/NA values for the key metrics
data <- data %>% 
  filter(!is.na(Precision), !is.na(Recall), !is.na(F1), !is.na(FP), !is.na(FN))
```

## Precision-Recall Plot with Improved Layout

```
# Create precision-recall plot for all samples excluding Giraffe(1kGP_pangenome)
create_pr_plot <- function(data) {
  # Filter out the 1kGP pangenome pipeline
  filtered_data <- data %>%
    filter(Pipeline != "Giraffe(1kGP_pangenome)")
  
  # Get all unique pipelines after filtering
  all_pipelines <- unique(filtered_data$Pipeline)
  filtered_colors <- pipeline_colors[names(pipeline_colors) %in% all_pipelines]
  
  # Calculate exact limits to minimize whitespace
  x_min <- floor(min(filtered_data$Precision, na.rm = TRUE) * 1000) / 1000
  x_max <- ceiling(max(filtered_data$Precision, na.rm = TRUE) * 1000) / 1000
  y_min <- floor(min(filtered_data$Recall, na.rm = TRUE) * 1000) / 1000
  y_max <- ceiling(max(filtered_data$Recall, na.rm = TRUE) * 1000) / 1000
  
  # Create faceted plot by Sample only with tighter layout
  pr_plot <- ggplot(filtered_data, aes(x = Precision, y = Recall, color = Pipeline)) +
    geom_point(size = 3, shape = 17) +
    scale_color_manual(values = filtered_colors) +
    facet_wrap(~ Sample, ncol = 5) +
    scale_x_continuous(
      labels = scales::percent_format(accuracy = 0.01),
      limits = c(x_min, x_max)
    ) +
    scale_y_continuous(
      labels = scales::percent_format(accuracy = 0.01),
      limits = c(y_min, y_max)
    ) +
    labs(
      x = "Precision",
      y = "Recall",
      color = "Pipeline"
    ) +
    theme_bw() +
    theme(
      legend.position = "bottom",
      legend.title = element_text(face = "bold", size = 10),
      strip.background = element_rect(fill = "white"),
      strip.text = element_text(face = "bold", size = 10),
      axis.text.x = element_text(angle = 45, hjust = 1, size = 7),
      axis.text.y = element_text(size = 7),
      axis.title = element_text(size = 10),
      panel.spacing = unit(0.1, "lines"),  # Minimal spacing between panels
      plot.margin = margin(2, 2, 2, 2),    # Minimal margins
      panel.grid.minor = element_blank()   # Remove minor grid lines
    )
  
  # Add optimal legend format
  pr_plot <- pr_plot + guides(color = guide_legend(nrow = 2, byrow = TRUE))
  
  return(pr_plot)
}

# Create and display the precision-recall plot
pr_plot <- create_pr_plot(data)
pr_plot
```

```
# Save the plot
ggsave("Figure_7.pdf", pr_plot, width = 12, height = 4, 
       units = "in", dpi = 300)
```

## Combined F1 Score and Total Errors Plot (Single Figure)

```
# Prepare data for both plots
filtered_data <- data %>%
  filter(Pipeline != "Giraffe(1kGP_pangenome)") %>%
  mutate(
    Total_Errors = FP + FN,
    F1_decimal = (F1 * 100) - 99,
    F1_from_base = F1_decimal - 0.40,
    # For Total Errors starting from 10000
    Total_Errors_from_base = Total_Errors - 10000
  ) %>%
  # Make Pipeline a factor with the defined order
  mutate(Pipeline = factor(Pipeline, levels = pipeline_order))

# Get unique pipelines for colors
all_pipelines <- unique(filtered_data$Pipeline)
filtered_colors <- pipeline_colors[names(pipeline_colors) %in% all_pipelines]

# Create the F1 Score plot
f1_plot <- ggplot(filtered_data, aes(x = Pipeline, y = F1_from_base, fill = Pipeline)) +
  # Add a baseline at 0 to represent 99.40%
  geom_hline(yintercept = 0, color = "gray80", linetype = "dashed") +
  geom_bar(stat = "identity", width = 1, position = position_dodge(width = 0)) +
  scale_fill_manual(values = filtered_colors) +
  facet_wrap(~ Sample, ncol = 5) +
  scale_y_continuous(
    labels = function(x) paste0(sprintf("%.2f", x + 99.40), "%"),
    breaks = seq(0, max(filtered_data$F1_from_base, na.rm = TRUE) + 0.05, by = 0.10),
    limits = c(0, max(filtered_data$F1_from_base, na.rm = TRUE) + 0.05),
    expand = c(0, 0)
  ) +
  labs(
    y = "F1 Score (%)",
    x = NULL
  ) +
  theme_bw() +
  theme(
    axis.text.x = element_blank(),
    axis.ticks.x = element_blank(),
    panel.grid.major.x = element_blank(),
    panel.grid.minor.x = element_blank(),
    panel.border = element_rect(color = "black", fill = NA),
    legend.position = "none",  # Remove legend from this plot
    strip.background = element_rect(fill = "white"),
    strip.text = element_text(face = "bold", size = 10),
    panel.spacing = unit(0.6, "lines"),
    plot.margin = margin(5, 5, 15, 5)
  )

# Create the Total Errors plot starting from 10000
errors_plot <- ggplot(filtered_data, aes(x = Pipeline, y = Total_Errors_from_base, fill = Pipeline)) +
  geom_hline(yintercept = 0, color = "gray80", linetype = "dashed") +
  geom_bar(stat = "identity", width = 1, position = position_dodge(width = 0)) +
  scale_fill_manual(values = filtered_colors) +
  facet_wrap(~ Sample, ncol = 5) +
  scale_y_continuous(
    labels = function(x) paste0(format(x + 10000, big.mark = ",")),
    expand = c(0, 0),
    limits = c(0, max(filtered_data$Total_Errors_from_base, na.rm = TRUE) + 1000)
  ) +
  labs(
    y = "Total Errors (FP + FN)",
    x = NULL
  ) +
  theme_bw() +
  theme(
    axis.text.x = element_blank(),
    axis.ticks.x = element_blank(),
    panel.grid.major.x = element_blank(),
    panel.grid.minor.x = element_blank(),
    panel.border = element_rect(color = "black", fill = NA),
    legend.position = "none",  # Remove legend from this plot
    strip.background = element_rect(fill = "white"),
    strip.text = element_text(face = "bold", size = 10),
    panel.spacing = unit(0.6, "lines"),
    plot.margin = margin(5, 5, 5, 5)
  )

# Create a separate legend that both plots can use
legend_plot <- ggplot(filtered_data, aes(x = Pipeline, y = 1, fill = Pipeline)) +
  geom_bar(stat = "identity") +
  scale_fill_manual(values = filtered_colors) +
  theme(legend.position = "bottom") +
  guides(fill = guide_legend(nrow = 2, byrow = TRUE, title = "Pipeline"))

# Extract the legend
legend <- cowplot::get_legend(legend_plot)

# Arrange the plots with the common legend and spacing
# Create empty plots for spacing
empty_plot1 <- ggplot() + theme_void()
empty_plot2 <- ggplot() + theme_void()

combined_plot <- gridExtra::grid.arrange(
  f1_plot,
  empty_plot1,  # Empty space between plots
  errors_plot,
  empty_plot2,  # Empty space before legend
  legend,
  heights = c(4.5, 0.5, 4.5, 0.5, 1.5),
  ncol = 1
)
```

```
# Display the combined plot
combined_plot
```

```
## TableGrob (5 x 1) "arrange": 5 grobs
##   z     cells    name              grob
## 1 1 (1-1,1-1) arrange    gtable[layout]
## 2 2 (2-2,1-1) arrange    gtable[layout]
## 3 3 (3-3,1-1) arrange    gtable[layout]
## 4 4 (4-4,1-1) arrange    gtable[layout]
## 5 5 (5-5,1-1) arrange gtable[guide-box]
```

```
# Save the combined plot
ggsave("Figure_S8.pdf", combined_plot, width = 16, height = 12, 
       units = "in", dpi = 300)
```

## Computational Overhead Plots

```
library(dplyr)
library(tidyr)
library(ggplot2)
library(ggpubr)
library(readr)

# -------------------------------------------------------------------
# Load updated long-format overhead file
# -------------------------------------------------------------------
perf <- read_csv("overhead_stats.csv", show_col_types = FALSE)

# Step ordering
perf$Step <- factor(
  perf$Step,
  levels = c("Alignment_lifting", "Indexing", "Personalization")
)

# Pipeline ordering (matching revision2_v2)
pipeline_levels <- c(
  "BWA-MEM",
  "Giraffe(linear)",
  "Giraffe(1kGP_pangenome)",
  "Giraffe(HPRC_pangenome)",
  "Giraffe(diploid)",
  "Giraffe(Imputefirst_c1)",
  "Giraffe(Imputefirst_c5)",
  "Giraffe(Imputefirst_c20)",
  "Leviosam2(Imputefirst_c1)",
  "Leviosam2(Imputefirst_c5)",
  "Leviosam2(Imputefirst_c20)"
)

perf$Pipeline <- factor(perf$Pipeline, levels = pipeline_levels)

# Pipeline color palette
pipeline_colors <- c(
  "BWA-MEM" = "#EF9A9A",
  "Giraffe(linear)" = "#D32F2F",
  "Giraffe(1kGP_pangenome)" = "#64B5F6",
  "Giraffe(HPRC_pangenome)" = "#1E88E5",
  "Giraffe(diploid)" = "#FB8C00",
  "Giraffe(Imputefirst_c1)" = "#4527A0",
  "Giraffe(Imputefirst_c5)" = "#673AB7",
  "Giraffe(Imputefirst_c20)" = "#311B92",
  "Leviosam2(Imputefirst_c1)" = "#AA00FF",
  "Leviosam2(Imputefirst_c5)" = "#E040FB",
  "Leviosam2(Imputefirst_c20)" = "#CE93D8"
)

# Step shading levels
step_alpha <- c(
  "Personalization" = 0.25,
  "Indexing" = 0.55,
  "Alignment_lifting" = 1.00
)

# -------------------------------------------------------------------
# PANEL A — Time (stacked + shaded)
# -------------------------------------------------------------------
time_plot <- ggplot(perf, aes(x = Pipeline, y = Time_min,
                              fill = Pipeline, alpha = Step)) +
  geom_bar(stat = "identity") +
  scale_fill_manual(values = pipeline_colors) +
  scale_alpha_manual(values = step_alpha, name = "Step") +
  labs(
    title = "Time Elapsed – Personalization, Indexing, Alignment & Lifting",
    y = "Time (minutes)", x = NULL
  ) +
  theme_bw() +
  theme(
    axis.text.x = element_blank(),
    axis.ticks.x = element_blank(),
    axis.text.y = element_text(size = 12),
    axis.title.y = element_text(size = 14),
    legend.position = "bottom",
    legend.title = element_text(size = 14),
    legend.text = element_text(size = 14),
    plot.title = element_text(size = 14)
  )

# -------------------------------------------------------------------
# PANEL B — Max RSS Memory
# -------------------------------------------------------------------
mem_data <- perf %>% group_by(Pipeline) %>% summarize(Memory_GB = max(Memory_GB))

mem_plot <- ggplot(mem_data, aes(x = Pipeline, y = Memory_GB, fill = Pipeline)) +
  geom_bar(stat = "identity") +
  scale_fill_manual(values = pipeline_colors) +
  labs(
    title = "Memory Usage – Max RSS Across Steps",
    y = "Max RSS (GB)", x = NULL
  ) +
  theme_bw() +
  theme(
    axis.text.x = element_blank(),
    axis.ticks.x = element_blank(),
    axis.text.y = element_text(size = 12),
    axis.title.y = element_text(size = 14),
    legend.position = "none",
    plot.title = element_text(size = 14)
  )

# -------------------------------------------------------------------
# PANEL C — CPU Hours (stacked + shaded)
# -------------------------------------------------------------------
cpu_plot <- ggplot(perf, aes(x = Pipeline, y = CPU_sec / 3600,
                             fill = Pipeline, alpha = Step)) +
  geom_bar(stat = "identity") +
  scale_fill_manual(values = pipeline_colors) +
  scale_alpha_manual(values = step_alpha, name = "Step") +
  labs(
    title = "CPU Hours – Personalization, Indexing, Alignment & Lifting",
    y = "CPU Time (hours)", x = NULL
  ) +
  theme_bw() +
  theme(
    axis.text.x = element_blank(),
    axis.ticks.x = element_blank(),
    axis.text.y = element_text(size = 12),
    axis.title.y = element_text(size = 14),
    legend.position = "none",
    plot.title = element_text(size = 14)
  )

# -------------------------------------------------------------------
# Combine A + B + C into one figure
# -------------------------------------------------------------------
combined_plot <- ggarrange(
  time_plot, mem_plot, cpu_plot,
  ncol = 3,
  common.legend = TRUE,
  legend = "bottom",
  labels = c("A","B","C")
)

combined_plot
```

```
ggsave(
  "Figure_8.pdf",
  combined_plot,
  width = 18, height = 6, dpi = 300
)
```
