# Supplementary material for: Minimizing reference bias with an imputed personalized reference
Source: Genome Res. 2026 Apr;36(4):740–53. doi: 10.1101/gr.280989.125 (PMC13138014; doi:10.1101/gr.280989.125)
Supplement: Supplement 1 [file Supplemental_Code.zip › imputefirst-main/plots_data_scripts/downstream_plots/Figure_S8.pdf]

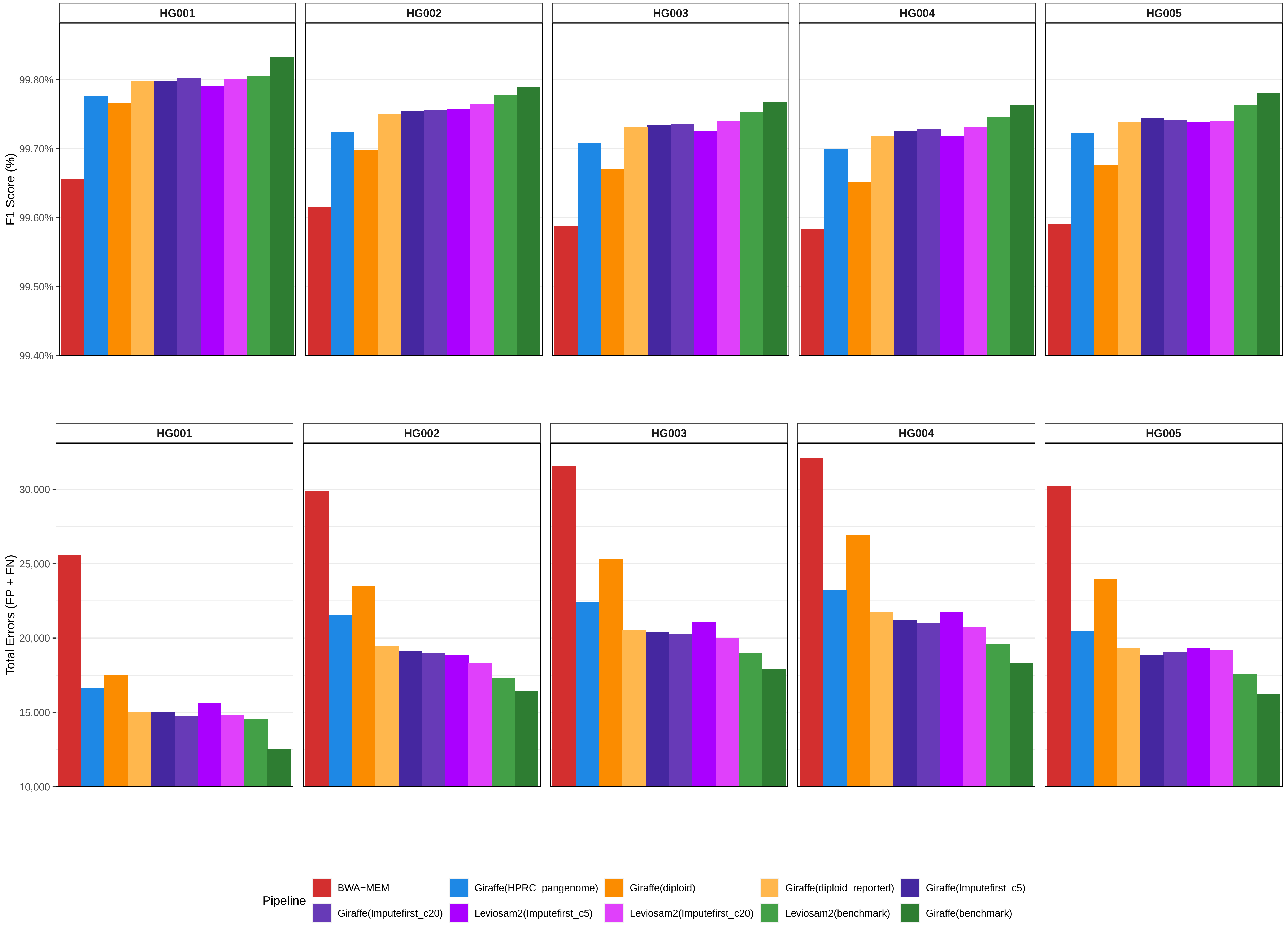

HG001
HG002
HG003
HG004
HG005
F1 Score (%)
99.80%
99.70%
99.60%
99.50%
99.40%
Total Errors (FP + FN)
30,000
25,000
20,000
15,000
10,000
Pipeline
BWA−MEM
Giraffe(HPRC_pangenome)
Giraffe(diploid)
Giraffe(diploid_reported)
Giraffe(Imputefirst_c5)
Giraffe(Imputefirst_c20)
Leviosam2(Imputefirst_c5)
Leviosam2(Imputefirst_c20)
Leviosam2(benchmark)
Giraffe(benchmark)
