# Supplementary material for: Minimizing reference bias with an imputed personalized reference
Source: Genome Res. 2026 Apr;36(4):740–53. doi: 10.1101/gr.280989.125 (PMC13138014; doi:10.1101/gr.280989.125)
Supplement: Supplement 1 [file Supplemental_Code.zip › imputefirst-main/plots_data_scripts/downstream_plots/biastools/Figure_5.pdf]

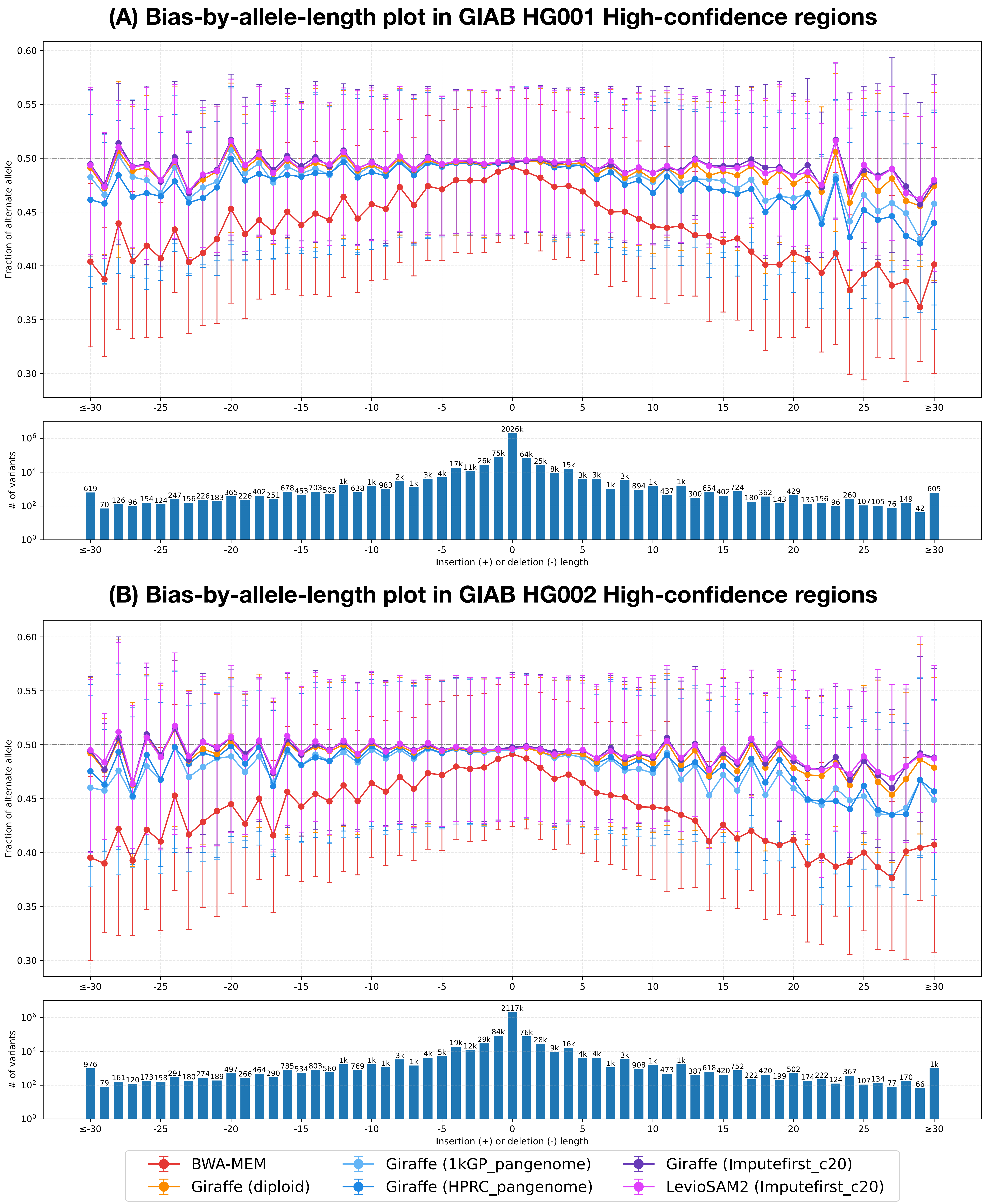
(A) Bias-by-allele-length plot in GIAB HG001 High-confidence regions
Fraction of alternate allele
# of variants
Insertion (+) or deletion (-) length
(B) Bias-by-allele-length plot in GIAB HG002 High-confidence regions
Fraction of alternate allele
# of variants
Insertion (+) or deletion (-) length
BWA-MEM
Giraffe (diploid)
Giraffe (1kGP_pangenome)
Giraffe (HPRC_pangenome)
Giraffe (Imputefirst_c20)
LevioSAM2 (Imputefirst_c20)
