# Supplementary material for: Minimizing reference bias with an imputed personalized reference
Source: Genome Res. 2026 Apr;36(4):740–53. doi: 10.1101/gr.280989.125 (PMC13138014; doi:10.1101/gr.280989.125)
Supplement: Supplement 1 [file Supplemental_Code.zip › imputefirst-main/plots_data_scripts/upstream_plots/upstream_plots.html]

plots revision1


# plots revision1

```
library(ggplot2)
library(ggh4x)
library(tidyr)
library(dplyr)
```

```
## 
## Attaching package: 'dplyr'
```

```
## The following objects are masked from 'package:stats':
## 
##     filter, lag
```

```
## The following objects are masked from 'package:base':
## 
##     intersect, setdiff, setequal, union
```

```
library(gridExtra)
```

```
## 
## Attaching package: 'gridExtra'
```

```
## The following object is masked from 'package:dplyr':
## 
##     combine
```

```
library(scales)
```

## Combined F1 Score Plot - Call Accuracy

```
# Load the data
callac <- read.csv('call_accuracy_filt_prep_f1.csv')
callac$method <- with(callac, reorder(method, altscore))

# Convert wide to long format
callac <- callac %>%
  gather(key = "score_type", value = "score_value", altscore, hetscore)

# Modify levels and labels
callac$imputetype <- factor(callac$imputetype,
    levels = c('calls(pre-impute)', 'beagle_impute', 'glimpse_impute'),
    labels = c('Genotyping','Beagle imputation','Glimpse imputation'))
callac <- callac %>% filter(method != 'pangenie(highGQ)')
callac$method <- factor(callac$method,
    levels = c('pangenie(highGQ)', 'bayestyper', 'bowtie2_bcftools', 'rowbowt'),
    labels = c('Pangenie (filtered)  ', 'Bayestyper  ', 'Bowtie 2 + BCFtools  ', 'rowbowt'))
callac <- callac[callac$fn %in% c('any'),] # keep only Any
callac$fn <- factor(callac$fn, levels = c('any'), labels = c('Any'))
callac$score_type <- factor(callac$score_type, levels = c('altscore', 'hetscore'), labels = c('ALT', 'HET'))
callac$variable <- as.character(callac$variable)
callac$variable <- factor(callac$variable, levels=c('0.01', '0.05', '0.1', '0.2', '0.5', '1', '2', '5', '10', '20'))

# Calculate global y-axis limits for consistent scaling
y_min_call <- floor(min(callac$score_value, na.rm = TRUE) * 1000) / 1000
y_max_call <- ceiling(max(callac$score_value, na.rm = TRUE) * 1000) / 1000

# Create grouped bar plot for call accuracy (2 rows × 1 column)
pdf("Figure_2.pdf", width=7, height=7)
pl <- ggplot(callac, aes(x = variable, y = score_value, fill = imputetype)) +
    geom_bar(stat = "identity", position = position_dodge(0.8), width = 0.7, alpha = 0.8, na.rm = TRUE) + 
    scale_fill_manual(values = c("Genotyping" = "#E69F00", "Beagle imputation" = "#56B4E9", "Glimpse imputation" = "#009E73")) +
    theme_bw() +
    theme(legend.direction = "horizontal", legend.box = "vertical",
          legend.position = "bottom", legend.margin = margin(t = -5),
          panel.border = element_rect(colour = "black", fill = NA)) +
    labs(x = "Coverage", y = "F1 score", fill="Method") +
    theme(axis.text.x = element_text(angle = 90, vjust = 0.5, hjust=1)) +
    scale_y_continuous(limits = c(y_min_call, y_max_call)) +
    ggh4x::facet_grid2(rows = vars(score_type))
pl
dev.off()
```

```
## quartz_off_screen 
##                 2
```

```
# Display the plot
pl
```

## window accuracy - beagle/glimpse - main plot

```
alldf.m <- read.csv("window_accuracy_filt_prep.csv")
mod_data <- gather(alldf.m,key="block",value="acc_value",5:7)
mod_data$variable <- as.character(alldf.m$variable)
mod_data$variable <- factor(mod_data$variable, levels=c('0.01', '0.05', '0.1', '0.2', '0.5', '1', '2', '5', '10', '20'))
mod_data$block = factor(mod_data$block, levels=c("m1_5","m6_10","m11_n"), labels=c("1 -- 5 (n = 17564978)","6 -- 10 (n = 1427164)","11+ (n = 371836)"))
mod_data$method <- factor(mod_data$method,
    levels = c('bayestyper', 'bowtie2_bcftools', 'rowbowt'),
    labels = c('Bayestyper  ', 'Bowtie 2 + BCFtools  ', 'rowbowt'))

# Create the data for the plot, filtering for the correct method and creating
# the full, descriptive labels for the legend.
plot_data <- mod_data %>%
  filter(grepl("Bowtie 2", method)) %>%
  mutate(
    imputation_method = ifelse(imputetype == "beagle", "Beagle", "Glimpse"),
    pipeline_label = paste(trimws(as.character(method)), imputation_method, sep = " + ")
  )

# Define the linetypes, using a clearer "longdash" for Beagle.
pipeline_linetypes <- c("Bowtie 2 + BCFtools + Glimpse" = "solid", "Bowtie 2 + BCFtools + Beagle" = "longdash")
# Define the single color for both lines.
pipeline_color <- "#F8766D" # Default ggplot red
pipeline_colors <- c("Bowtie 2 + BCFtools + Glimpse" = pipeline_color, "Bowtie 2 + BCFtools + Beagle" = pipeline_color)

window_plot <- ggplot(data = plot_data, aes(x = variable, y = acc_value, group = pipeline_label, colour = pipeline_label, linetype = pipeline_label)) +
  geom_line(size = 0.75) +
  # Use manual scales to control appearance and set name = NULL to remove the legend title.
  scale_linetype_manual(name = NULL, values = pipeline_linetypes) +
  scale_colour_manual(name = NULL, values = pipeline_colors) +
  theme_bw() +
  theme(
    legend.direction = "horizontal",
    legend.box = "vertical",
    legend.position = "bottom",
    legend.margin = margin(t = -5),
    panel.border = element_rect(colour = "black", fill = NA),
    text = element_text(size = 14, face = "plain"),
    axis.text = element_text(size = 8, face = "plain"),
    legend.text = element_text(size = 12, face = "plain"),
    axis.text.x = element_text(angle = 90, vjust = 0.5, hjust = 1),
    # Make the legend key wider to clearly show the dash pattern
    legend.key.width = unit(1.2, "cm")
  ) +
  labs(x = "Coverage", y = "% matching 200bp window") +
  scale_y_continuous(limits = NULL) +
  ggh4x::facet_grid2(~block, scales = "free_y", independent = "y")
```

```
## Warning: Using `size` aesthetic for lines was deprecated in ggplot2 3.4.0.
## ℹ Please use `linewidth` instead.
## This warning is displayed once every 8 hours.
## Call `lifecycle::last_lifecycle_warnings()` to see where this warning was generated.
```

```
ggsave("Figure_3.pdf", window_plot, device = "pdf", width = 7, height = 4)
window_plot
```

## Supplementary Figure: ALT/HET F1 Score Comparison

```
# --- Libraries ---
library(ggplot2)
library(dplyr)
library(tidyr)
library(ggh4x)

# --- Load + prep ---
alldf <- read.csv("call_accuracy_filt_prep_f1.csv")

# Labels & ordering
alldf$imputetype <- factor(
  alldf$imputetype,
  levels = c('calls(pre-impute)', 'beagle_impute', 'glimpse_impute'),
  labels = c('Genotyping','Beagle imputation','Glimpse imputation')
)
alldf$fn <- factor(
  alldf$fn,
  levels = c('snp','indel','sv','any'),
  labels = c('SNV','Indel','SV','Any')
)
alldf$variable <- factor(as.character(alldf$variable),
  levels = c('0.01','0.05','0.1','0.2','0.5','1','2','5','10','20')
)

# Drop pangenie
alldf <- alldf %>% filter(!grepl("pangenie", method, ignore.case = TRUE))

# Long format: ALT/HET
alldf_long <- alldf %>%
  pivot_longer(
    cols = c(altscore, hetscore),
    names_to = "score_type",
    values_to = "score_value"
  )
alldf_long$score_type <- factor(
  alldf_long$score_type,
  levels = c("altscore","hetscore"),
  labels = c("ALT","HET")
)

# --- Per-row (by variant type) y-limits shared across ALT & HET in that row ---
row_lims <- alldf_long %>%
  group_by(fn) %>%
  summarise(
    ymin = floor(min(score_value, na.rm = TRUE) * 1000) / 1000,
    ymax = ceiling(max(score_value, na.rm = TRUE) * 1000) / 1000,
    .groups = "drop"
  )

# Build scales list in row-major order (SNV, Indel, SV, Any) × (ALT, HET)
fn_levels <- levels(alldf_long$fn)  # c("SNV","Indel","SV","Any")
y_scales <- unlist(lapply(fn_levels, function(f) {
  lims <- with(row_lims[row_lims$fn == f, ], c(ymin, ymax))
  list(
    scale_y_continuous(limits = lims),  # ALT for this row
    scale_y_continuous(limits = lims)   # HET for this row
  )
}), recursive = FALSE)

# --- Plot: 4 rows (variant types) × 2 cols (ALT/HET), with per-row matched y-lims ---
suppl_alt_het_4x2 <- ggplot(
  alldf_long,
  aes(x = variable, y = score_value, fill = imputetype)
) +
  geom_bar(
    stat = "identity",
    position = position_dodge(width = 0.8),
    width = 0.7,
    alpha = 0.8,
    na.rm = TRUE
  ) +
  scale_fill_manual(
    values = c(
      "Genotyping" = "#E69F00",
      "Beagle imputation" = "#56B4E9",
      "Glimpse imputation" = "#009E73"
    )
  ) +
  theme_bw() +
  theme(
    legend.direction = "horizontal",
    legend.box = "vertical",
    legend.position = "bottom",
    legend.margin = margin(t = -5),
    panel.border = element_rect(colour = "black", fill = NA),
    text = element_text(size = 12, face = "plain"),
    axis.text = element_text(size = 8, face = "plain"),
    axis.text.x = element_text(angle = 90, vjust = 0.5, hjust = 1)
  ) +
  labs(x = "Coverage", y = "F1 score", fill = "Method") +
  ggh4x::facet_grid2(
    rows = vars(fn),           # SNV, Indel, SV, Any
    cols = vars(score_type),   # ALT | HET
    scales = "free_y",         # allow different y across rows
    independent = "y"          # but ALT/HET share per-row limits via facetted_pos_scales
  ) +
  ggh4x::facetted_pos_scales(
    y = y_scales
  )

# Save + show
ggsave("Figure_S1.pdf", suppl_alt_het_4x2,
       #device = "pdf", width = 12, height = 10)
       device = "pdf", width = 7, height = 7)
suppl_alt_het_4x2
```

## Supplementary Figure: Computation Time and Memory Usage

```
# --- Libraries ---
library(ggplot2)
library(dplyr)
library(tidyr)
library(ggh4x)

# --- Read + join time/mem stats ---
p1_t <- read.csv('phase1_time_stats.csv')   # preimpute time
p1_m <- read.csv('phase1_mem_stats.csv')    # preimpute mem
p1    <- inner_join(p1_t, p1_m)
```

```
## Joining with `by = join_by(bcategory, method, variable)`
```

```
imp_t <- read.csv('impute_time_stats.csv')  # imputation time
imp_m <- read.csv('impute_mem_stats.csv')   # imputation mem
impstat <- inner_join(imp_t, imp_m)
```

```
## Joining with `by = join_by(bcategory, method, variable)`
```

```
uber <- full_join(impstat, p1)
```

```
## Joining with `by = join_by(bcategory, method, variable, timeval, memval)`
```

```
# --- Clean / relabel ---
uber <- uber %>%
  filter(method != 'pangenie',
         bcategory != 'glimpse_unphased',
         bcategory != 'total')

uber$variable <- factor(as.character(uber$variable),
                        levels = c('0.01','0.05','0.1','0.2','0.5','1','2','5'))

uber$bcategory <- factor(uber$bcategory,
                         levels = c("preimpute","glimpse_phased","beagle"),
                         labels = c("Genotyping","Glimpse imputation","Beagle imputation"))

uber$method <- factor(uber$method,
    levels = c('pangenie(highGQ)', 'bayestyper', 'bowtie2_bcftools', 'rowbowt'),
    labels = c('Pangenie (filtered)  ', 'Bayestyper  ', 'Bowtie 2 + BCFtools  ', 'rowbowt'))

# --- Keep a single pipeline (to match your screenshot) ---
# Change this regex if you want a different 'method'
method_regex <- "Bowtie 2 \\+ BCFtools"
uber <- uber %>% filter(grepl(method_regex, method))

# --- Long format: time + mem ---
uber_long <- uber %>%
  pivot_longer(
    cols = c(timeval, memval),
    names_to = "measurement",
    values_to = "value"
  ) %>%
  mutate(
    measurement = factor(measurement,
                         levels = c("timeval","memval"),
                         labels = c("Time (minutes)", "Memory (GB)"))
  )

# --- Colors for stages (match your earlier scheme) ---
stage_cols <- c(
  "Genotyping" = "#E69F00",
  "Glimpse imputation" = "#009E73",
  "Beagle imputation" = "#56B4E9"
)

# --- 2-row grouped-bar plot (Time on top, Memory on bottom) ---
compute_2x1 <- ggplot(
  uber_long,
  aes(x = variable, y = value, fill = bcategory)
) +
  geom_bar(
    stat = "identity",
    position = position_dodge2(width = 0.8, preserve = "single"),
    width = 0.7,
    alpha = 0.9,
    na.rm = TRUE
  ) +
  scale_fill_manual(values = stage_cols, name = "Method") +
  theme_bw() +
  theme(
    legend.position = "bottom",
    legend.margin = margin(t = -5),
    panel.border = element_rect(colour = "black", fill = NA),
    text = element_text(size = 12),
    axis.text = element_text(size = 9),
    axis.text.x = element_text(angle = 90, vjust = 0.5, hjust = 1),
    strip.background = element_rect(fill = "grey90", colour = "grey60")
  ) +
  labs(x = "Coverage", y = NULL) +
  ggh4x::facet_grid2(
    rows = vars(measurement),  # Row 1: Time, Row 2: Memory
    scales = "free_y",         # different units → free y-scale per row
    independent = "y"
  )

# Save + show
#ggsave("personalized_compute_grouped_2x1.pdf", compute_2x1, width = 10, height = 6)
ggsave("Figure_S2.pdf", compute_2x1, width = 7, height = 7)
compute_2x1
```

## panels comparison plot for HG002

```
# Load the data from the CSV file
data <- read.csv("panels_accuracy_happy.csv")

# Filter the data for mode 'all' 
data_all <- subset(data, Mode == "all")

# Factor levels for pipelines and panels for clarity in the plot
data_all$Pipeline <- factor(data_all$Pipeline, levels = c('BBBC5', 'BBBC20', 'BBBC30'))

# Panel order 
data_all$Panel <- factor(data_all$Panel, 
                         levels = c('HPRC_original', 'HPRC_filtered', 'HGSVC2', 'HGSVC3'))

# Color palette - more distinct but professional
#panel_colors <- c("#4575B4", "#74ADD1", "#D73027", "#FC8D59")  # Blue, Light blue, Red, Light red/orange
panel_colors <- c("#4575B4","#FC8D59","#D73027")  # Blue, Light blue, Red, Light red/orange

# Calculate F-measure if not already in the data
if(!"F_measure" %in% colnames(data_all)) {
  data_all$F_measure <- 2 * (data_all$Precision * data_all$Sensitivity) / (data_all$Precision + data_all$Sensitivity)
}

# Create the combined plot with shared legend
combined_plot <- function() {
  # Calculate exact limits to minimize whitespace
  x_min <- floor(min(data_all$Precision) * 1000) / 1000
  x_max <- ceiling(max(data_all$Precision) * 1000) / 1000
  y_min <- floor(min(data_all$Sensitivity) * 1000) / 1000
  y_max <- ceiling(max(data_all$Sensitivity) * 1000) / 1000

  # Precision-Recall Plot
  p1 <- ggplot(data_all, aes(x = Precision, y = Sensitivity, color = Panel, shape = Pipeline)) +
    geom_point(size = 2.5) +
    scale_shape_manual(values = c(17, 15, 16)) + # Different shapes for Pipelines
    scale_color_manual(values = panel_colors) +
    labs(y = "Recall", x = "Precision") +
    # Format axes with percentage
    scale_y_continuous(
      labels = scales::percent_format(accuracy = 0.01),
      limits = c(y_min, y_max)
    ) +
    scale_x_continuous(
      labels = scales::percent_format(accuracy = 0.01),
      limits = c(x_min, x_max)
    ) +
    theme_bw() +
    theme(
      legend.position = "none",
      axis.text.x = element_text(angle = 90, vjust = 0.5, hjust = 1, size = 8, face = "plain"),
      axis.text.y = element_text(size = 8, face = "plain"),
      axis.title.x = element_text(size = 12, face = "plain"),
      axis.title.y = element_text(size = 12, face = "plain"),
      panel.border = element_rect(color = "black", fill = NA),
      panel.grid.minor = element_blank(),
      plot.margin = margin(2, 5, 2, 2)
    )

  # F-measure Bar Plot
  p2 <- ggplot(data_all, aes(x = Pipeline, y = F_measure, fill = Panel)) +
    geom_bar(stat = "identity", position = position_dodge(width = 0.6), width = 0.5) +
    scale_fill_manual(values = panel_colors) +
    labs(y = "F1 score", x = "Pipeline") +
    coord_cartesian(ylim = c(0.85, max(data_all$F_measure) * 1.01)) +
    scale_y_continuous(
      labels = scales::number_format(accuracy = 0.001),
      breaks = seq(0.85, 0.96, by = 0.01)
    ) +
    theme_bw() +
    theme(
      legend.position = "none",
      axis.text.x = element_text(angle = 0, size = 8, face = "plain"),
      axis.text.y = element_text(size = 8, face = "plain"),
      axis.title.x = element_text(size = 12, face = "plain"),
      axis.title.y = element_text(size = 12, face = "plain"),
      panel.border = element_rect(color = "black", fill = NA),
      panel.grid.minor = element_blank(),
      plot.margin = margin(2, 2, 2, 5)
    )

  # Get the legend
  legend_data <- ggplot(data_all, aes(x = Precision, y = Sensitivity, color = Panel, shape = Pipeline)) +
    geom_point() +
    scale_shape_manual(values = c(17, 15, 16)) +
    scale_color_manual(values = panel_colors) +
    labs(color = "Panel", shape = "Pipeline") +
    theme_bw() +
    theme(legend.position = "bottom",
          legend.direction = "horizontal",
          legend.box = "vertical",
          legend.margin = margin(t = -1),
          legend.text = element_text(size = 10, face = "plain"),
          legend.title = element_text(size = 10, face = "plain"))

  # Extract legend
  tmp <- ggplot_gtable(ggplot_build(legend_data))
  leg <- which(sapply(tmp$grobs, function(x) x$name) == "guide-box")
  legend <- tmp$grobs[[leg]]

  # Arrange the plots with the legend
  grid.arrange(
    arrangeGrob(p1, p2, ncol = 2, widths = c(1, 1)),
    legend,
    heights = c(5, 2), 
    nrow = 2
  )
}

# Generate and display the plot
plot_result <- combined_plot()
```

```
print(plot_result)
```

```
## TableGrob (2 x 1) "arrange": 2 grobs
##   z     cells    name              grob
## 1 1 (1-1,1-1) arrange   gtable[arrange]
## 2 2 (2-2,1-1) arrange gtable[guide-box]
```

```
# Save the plot
ggsave("Figure_4.pdf", plot_result, width = 7, height = 5, units = "in", dpi = 300)
```

## suppl plot for rest of samples HG001-HG005 on HGSVC3 accuracy for 5x,20x,30x wrt GT.

## Per-sample accuracy comparison (final compact layout)

```
data <- read.csv("samples_panel_accuracy_happy.csv")
data_all <- subset(data, Mode == "all")
data_all$Pipeline <- factor(data_all$Pipeline, levels = c("BBBC5", "BBBC20", "BBBC30"))
data_all$Sample <- factor(data_all$Sample, levels = c("HG001", "HG003", "HG004", "HG005"))
sample_colors <- c("HG001" = "#1b9e77", "HG003" = "#d95f02", "HG004" = "#7570b3", "HG005" = "#e7298a")
pipeline_shapes <- c("BBBC5" = 17, "BBBC20" = 15, "BBBC30" = 16)

# Precision-Recall Plot (all samples together)
p_pr <- ggplot(data_all, aes(x = Precision, y = Sensitivity, shape = Pipeline, color = Sample)) +
  geom_point(size = 2.5) +
  scale_color_manual(values = sample_colors) +
  scale_shape_manual(values = pipeline_shapes) +
  labs(x = "Precision", y = "Recall") +
  scale_x_continuous(labels = scales::percent_format(accuracy = 0.01)) +
  scale_y_continuous(labels = scales::percent_format(accuracy = 0.01)) +
  theme_bw() +
  theme(
    legend.position = "none",
    axis.text = element_text(size = 8),
    axis.title = element_text(size = 10),
    panel.border = element_rect(color = "black"),
    panel.grid.minor = element_blank(),
    plot.margin = margin(4, 4, 4, 4)
  )

# F1 Score Bar Plot (compact bars)
p_f1 <- ggplot(data_all, aes(x = Pipeline, y = F_measure, fill = Sample)) +
  geom_bar(stat = "identity", position = position_dodge(width = 0.4), width = 0.35) +
  scale_fill_manual(values = sample_colors) +
  labs(x = "Pipeline", y = "F1 Score") +
  coord_cartesian(ylim = c(0.85, 0.96)) +
  scale_y_continuous(labels = scales::number_format(accuracy = 0.001)) +
  theme_bw() +
  theme(
    legend.position = "none",
    axis.text = element_text(size = 8),
    axis.title = element_text(size = 10),
    panel.border = element_rect(color = "black"),
    panel.grid.minor = element_blank(),
    plot.margin = margin(4, 4, 4, 4)
  )

# Shared Legend
legend_plot <- ggplot(data_all, aes(x = Precision, y = Sensitivity, color = Sample, shape = Pipeline)) +
  geom_point() +
  scale_color_manual(values = sample_colors) +
  scale_shape_manual(values = pipeline_shapes) +
  labs(color = "Sample", shape = "Pipeline") +
  theme_bw() +
  theme(
    legend.position = "bottom",
    legend.spacing.x = unit(0.2, 'cm'),
    legend.key.width = unit(0.3, 'cm'),
    legend.text = element_text(size = 9),
    legend.title = element_text(size = 10)
  )

legend_grob <- ggplotGrob(legend_plot)
legend <- legend_grob$grobs[[which(sapply(legend_grob$grobs, function(x) x$name) == "guide-box")]]

# Combine
combined_sample_plot <- grid.arrange(
  arrangeGrob(p_pr, p_f1, ncol = 2, widths = c(1, 1.05)),
  legend,
  heights = c(4.1, 0.8)
)
```

```
# Save compact version
ggsave("Figure_S3.pdf", combined_sample_plot, width = 6.8, height = 4.4, units = "in")

combined_sample_plot
```

```
## TableGrob (2 x 1) "arrange": 2 grobs
##   z     cells    name              grob
## 1 1 (1-1,1-1) arrange   gtable[arrange]
## 2 2 (2-2,1-1) arrange gtable[guide-box]
```
