# Supplementary figures and images for: Minimizing reference bias with an imputed personalized reference
Source: Genome Res. 2026 Apr;36(4):740–53. doi: 10.1101/gr.280989.125 (PMC13138014; doi:10.1101/gr.280989.125)

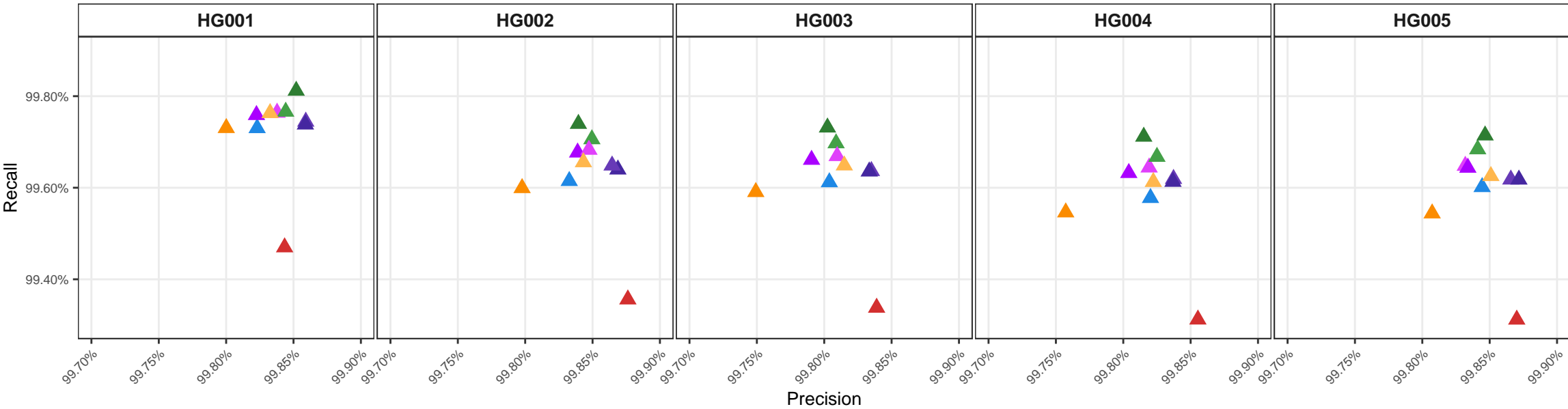

Supplement: Supplement 1 [file Supplemental_Code.zip › imputefirst-main/plots_data_scripts/downstream_plots/Figure_7.pdf]

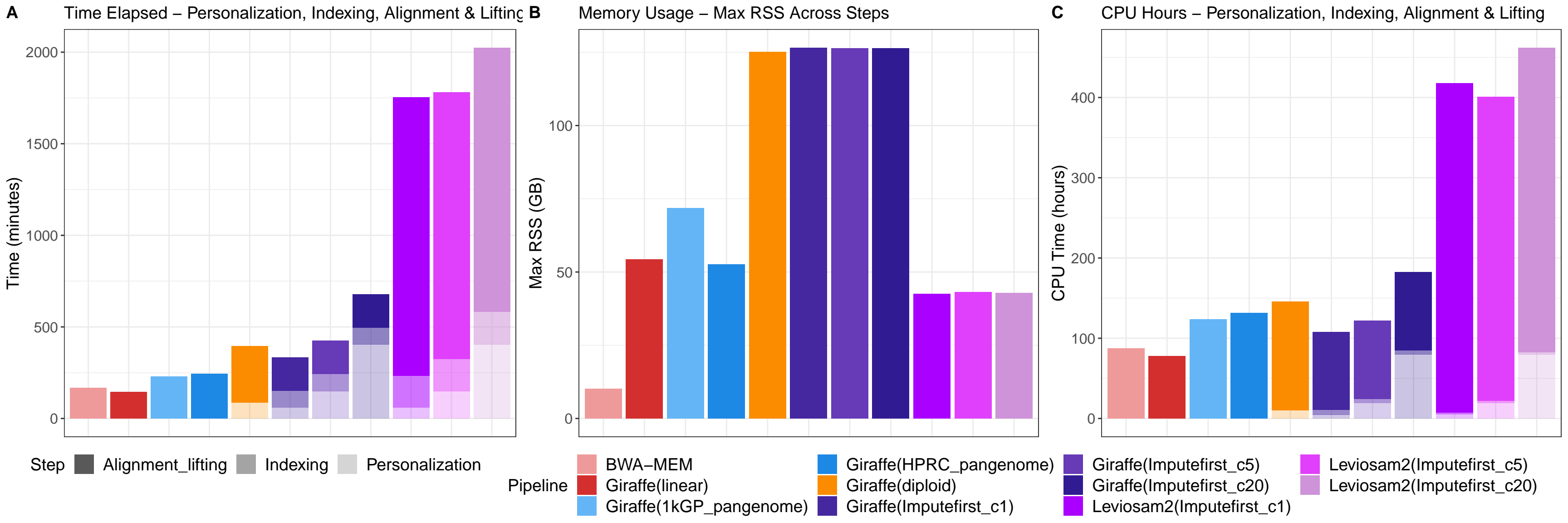

Supplement: Supplement 1 [file Supplemental_Code.zip › imputefirst-main/plots_data_scripts/downstream_plots/Figure_8.pdf]

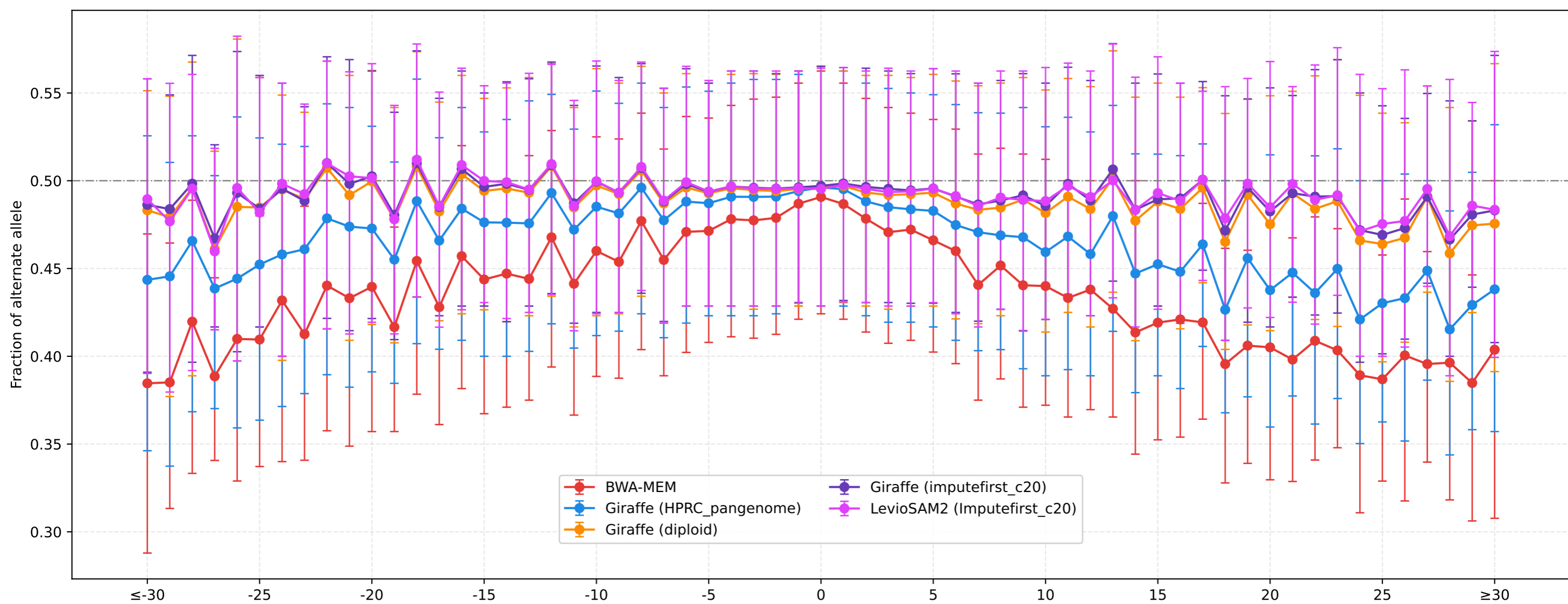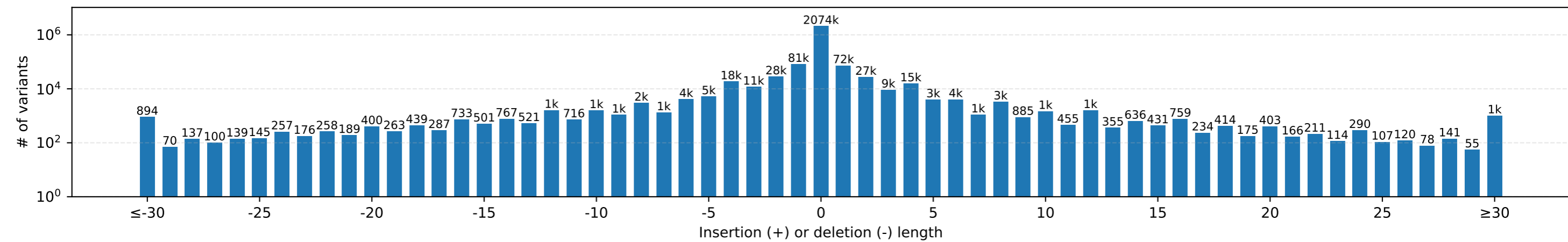

Supplement: Supplement 1 [file Supplemental_Code.zip › imputefirst-main/plots_data_scripts/downstream_plots/biastools/Figure_S5.pdf]

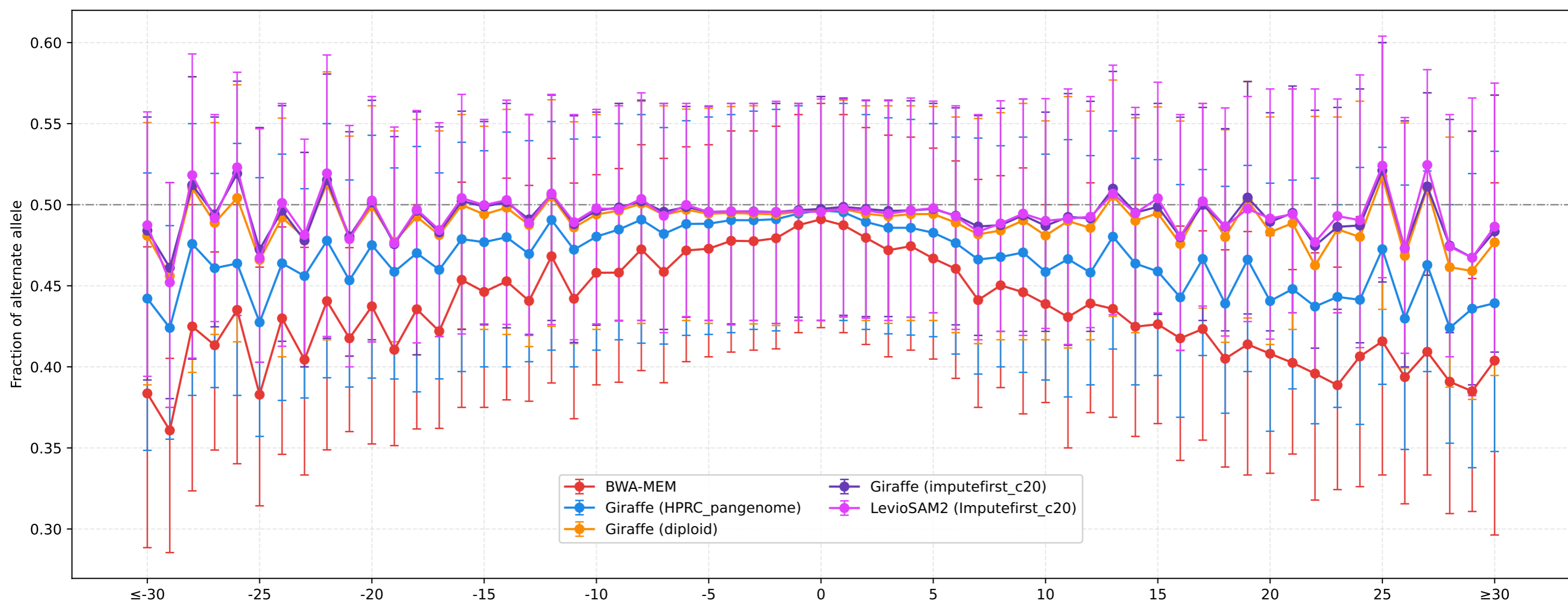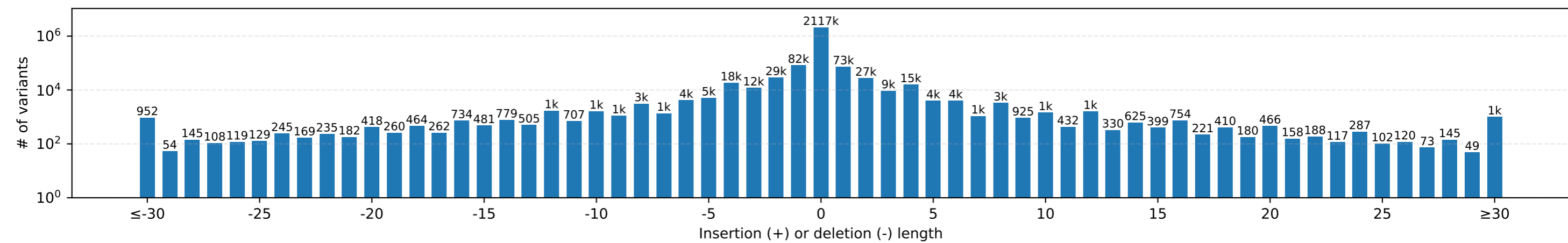

Supplement: Supplement 1 [file Supplemental_Code.zip › imputefirst-main/plots_data_scripts/downstream_plots/biastools/Figure_S6.pdf]

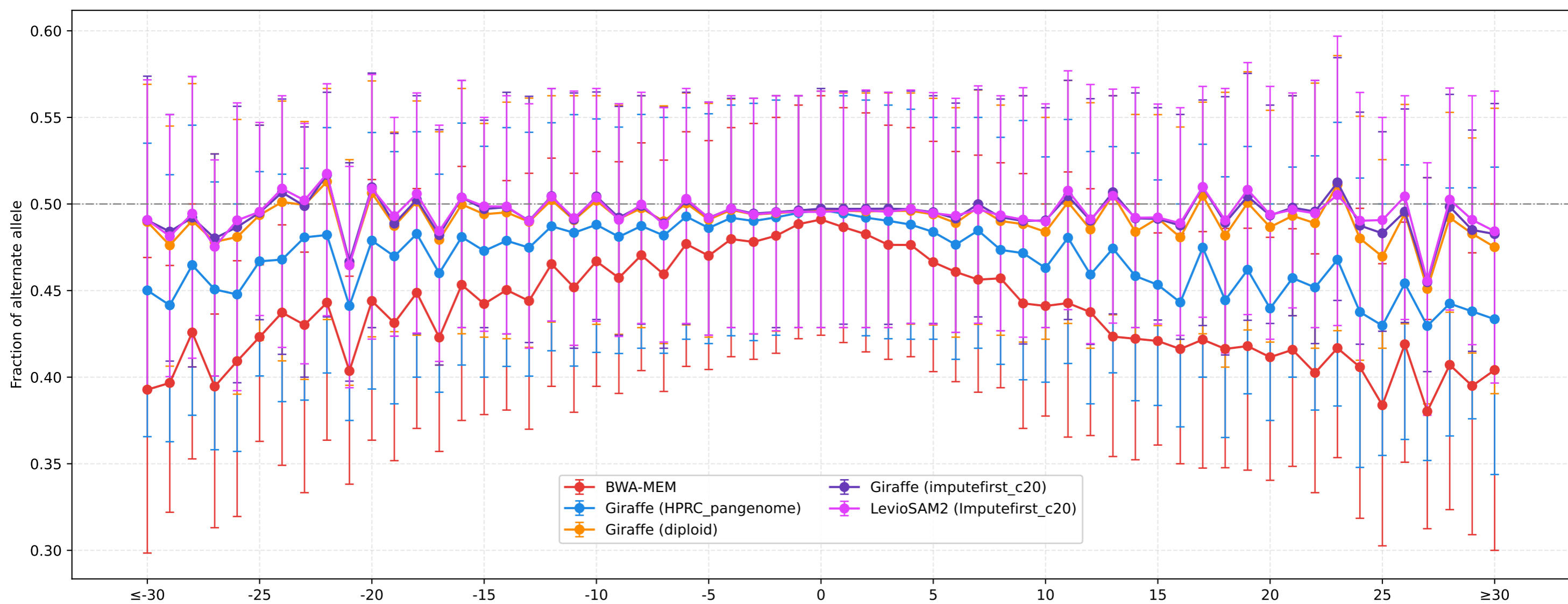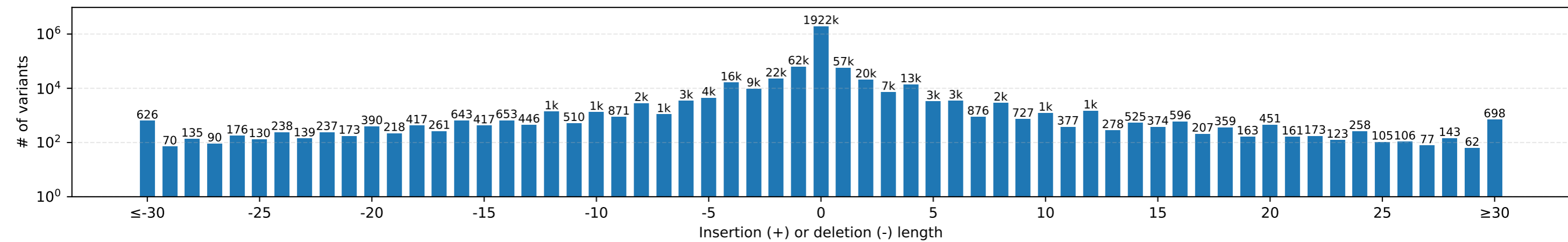

Supplement: Supplement 1 [file Supplemental_Code.zip › imputefirst-main/plots_data_scripts/downstream_plots/biastools/Figure_S7.pdf]

SNVs

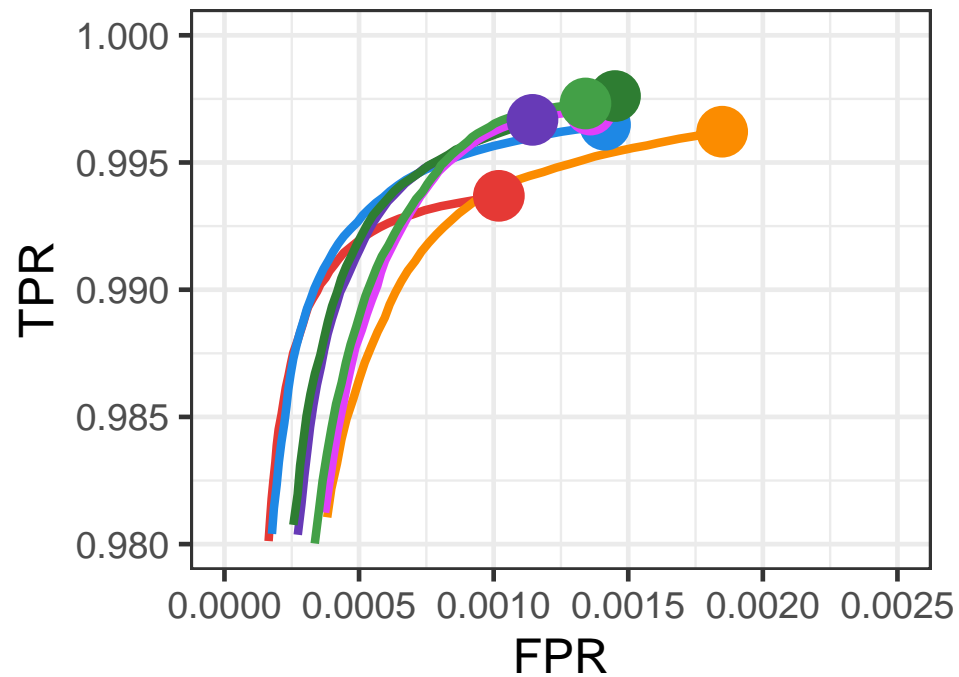

indel

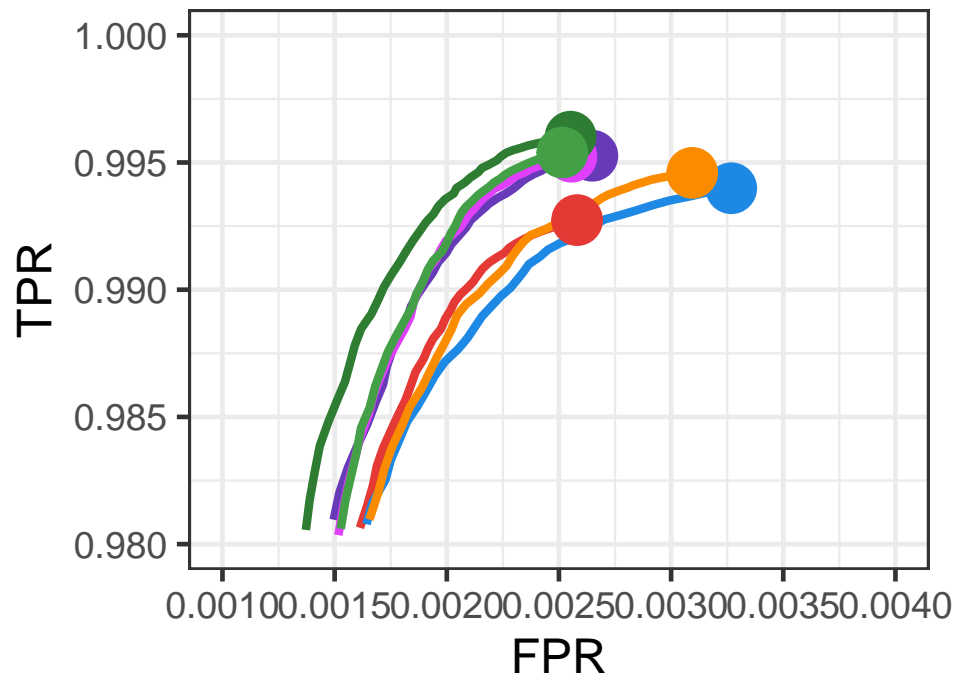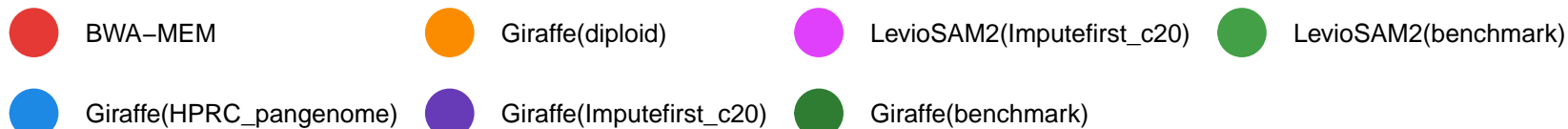

Supplement: Supplement 1 [file Supplemental_Code.zip › imputefirst-main/plots_data_scripts/downstream_plots/ROC_plots/Figure_6.pdf]

HG001 – SNP

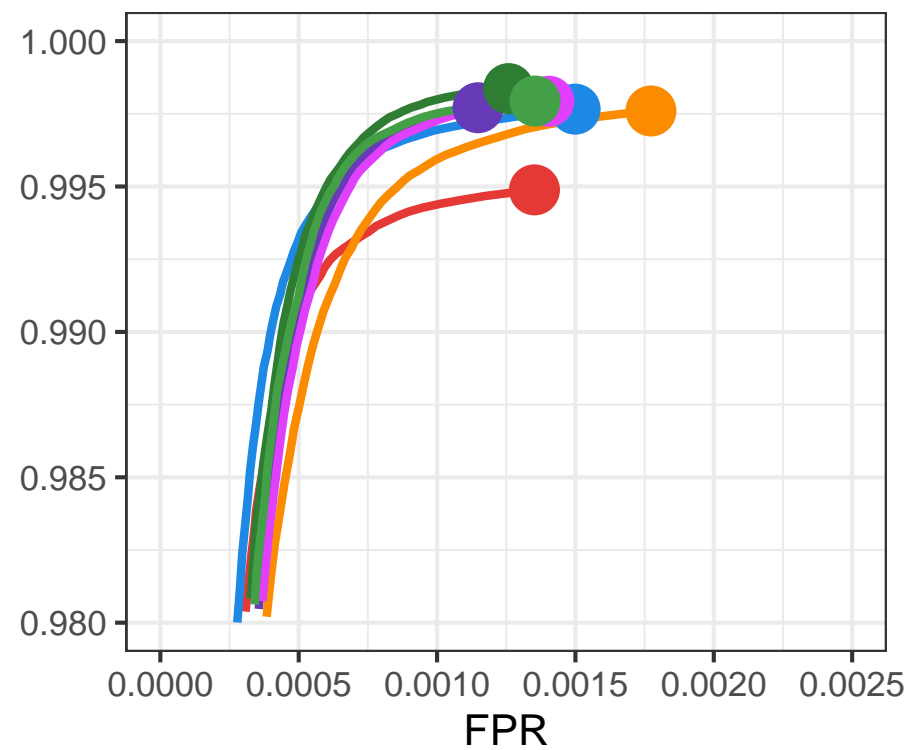

HG003 – SNP

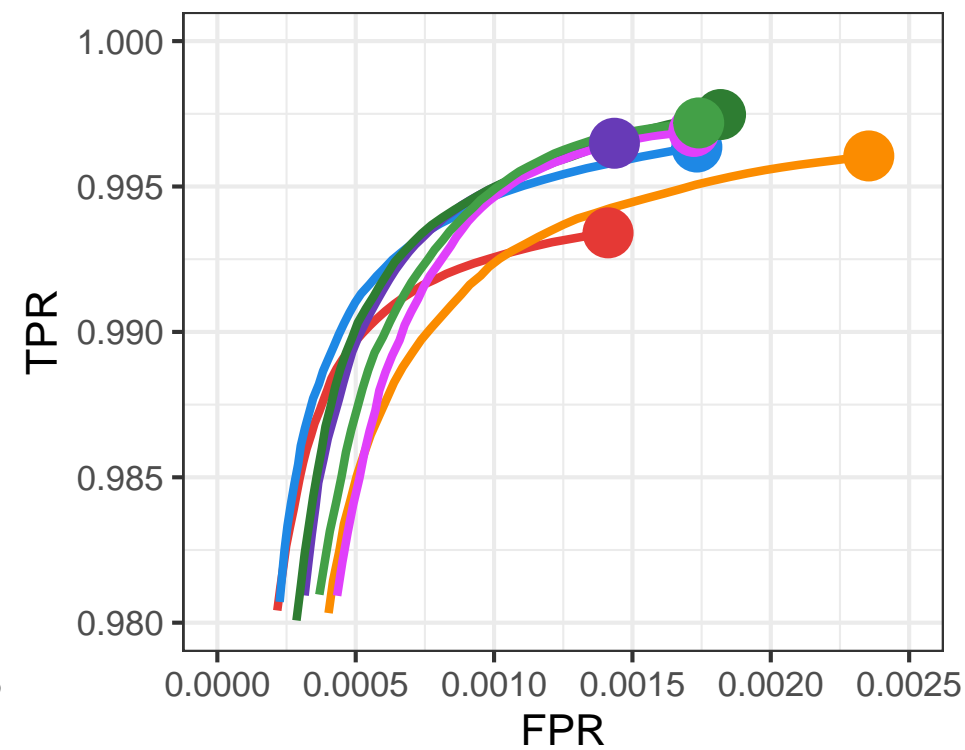

HG004 – SNP

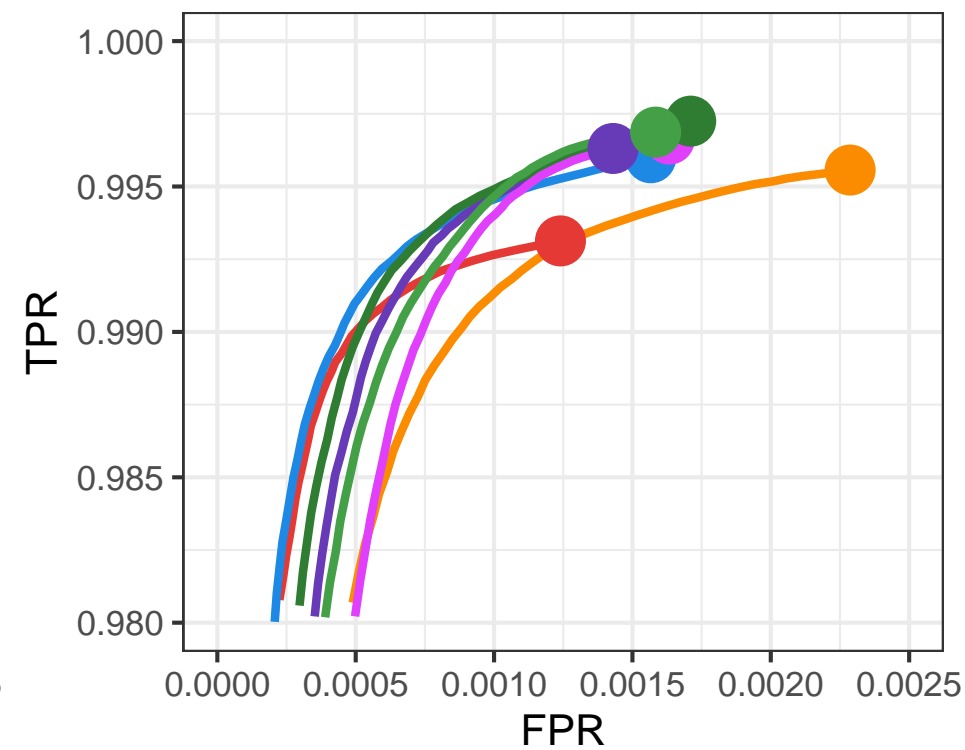

HG005 – SNP

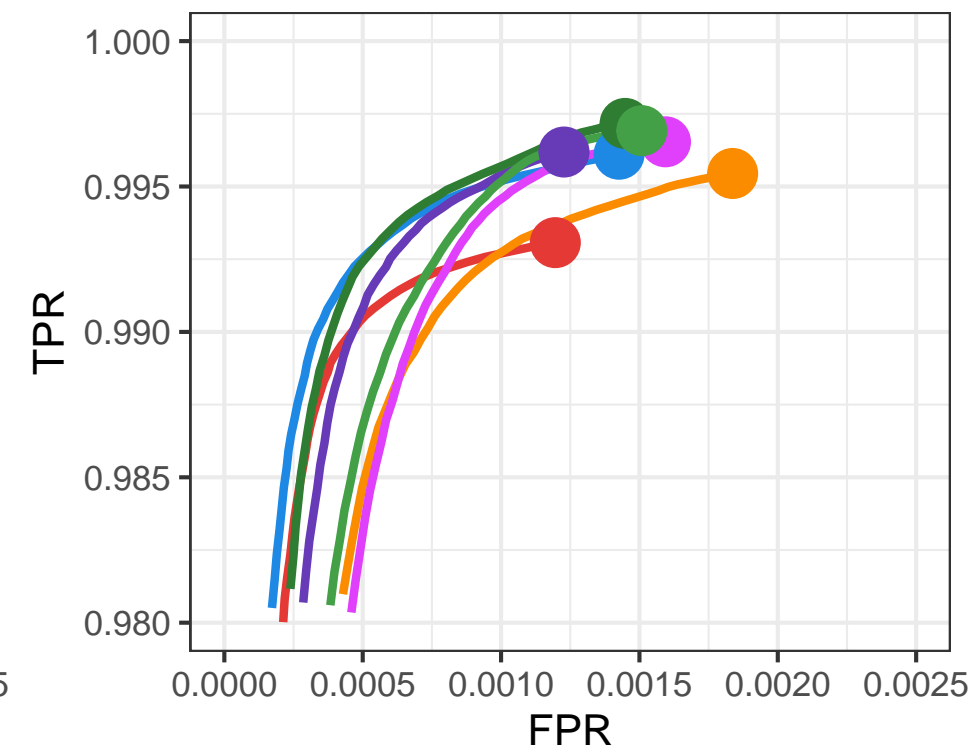

HG001 – INDEL

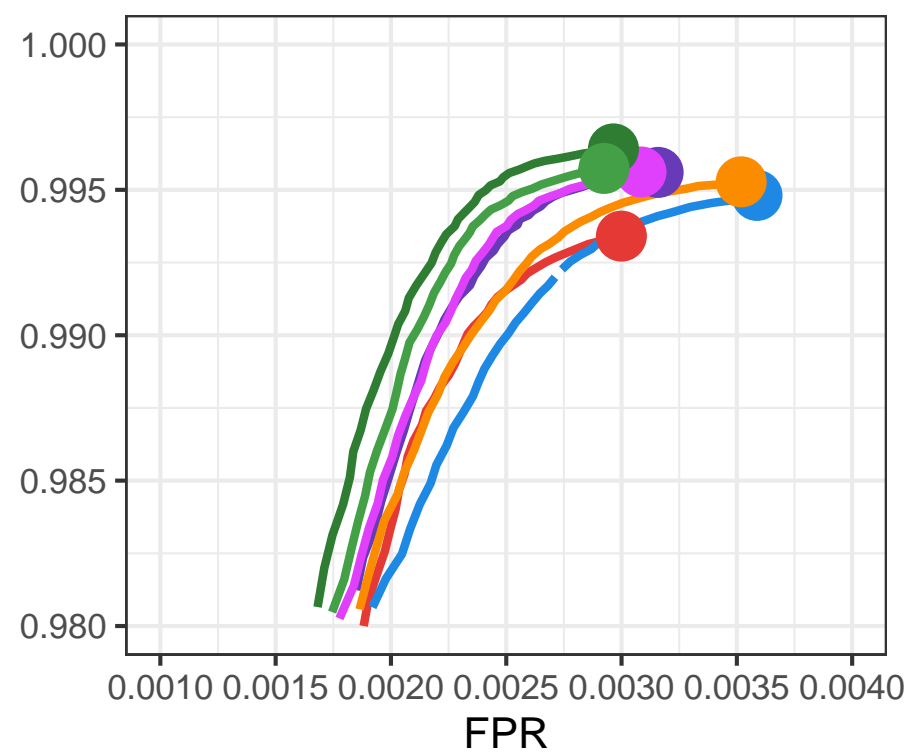

HG003 – INDEL

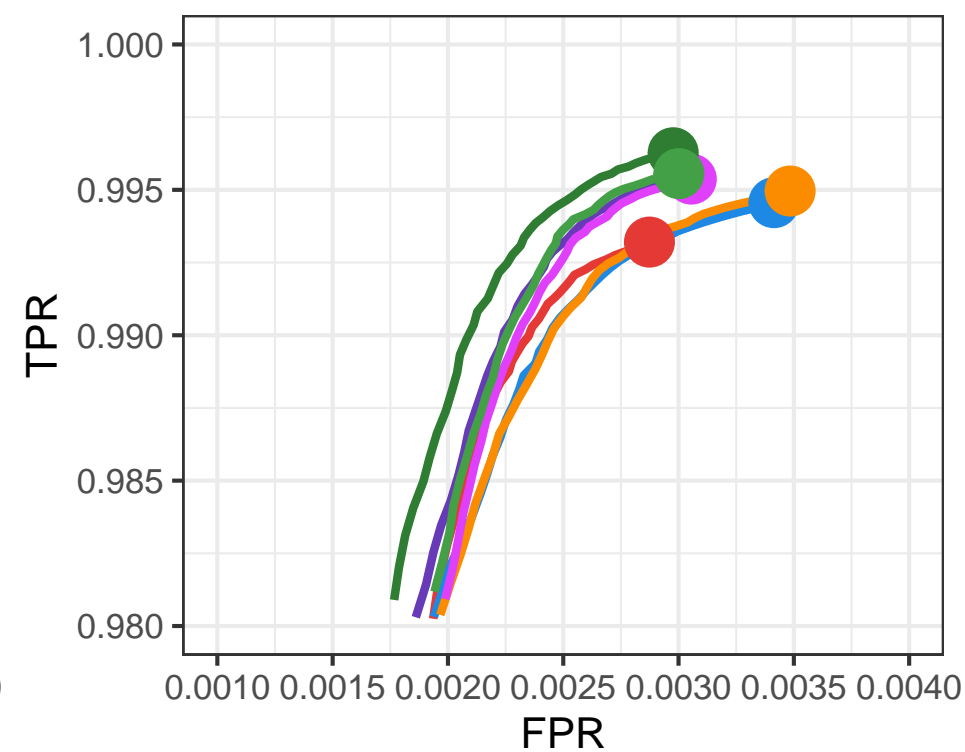

HG004 – INDEL

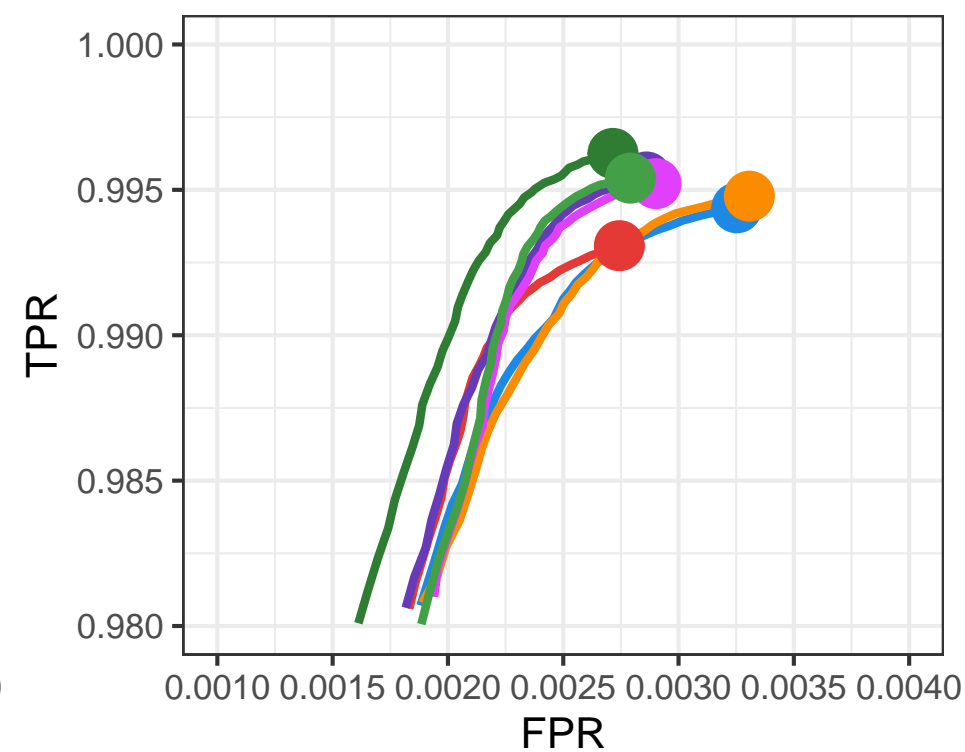

HG005 – INDEL

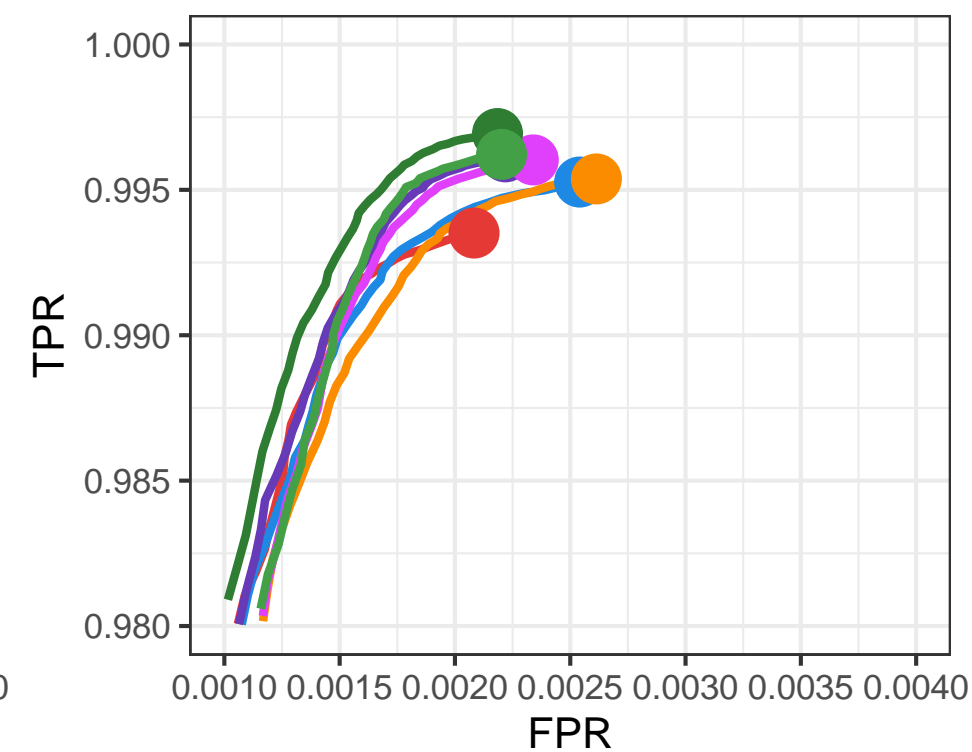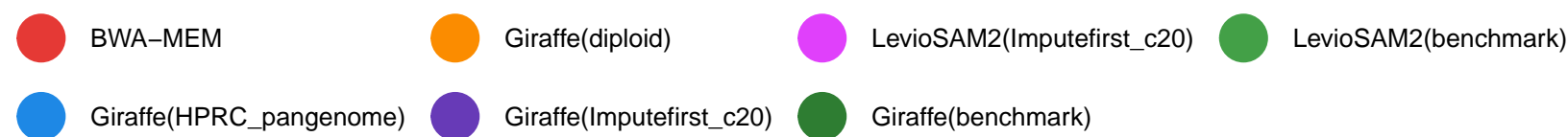

Supplement: Supplement 1 [file Supplemental_Code.zip › imputefirst-main/plots_data_scripts/downstream_plots/ROC_plots/Figure_S9.pdf]

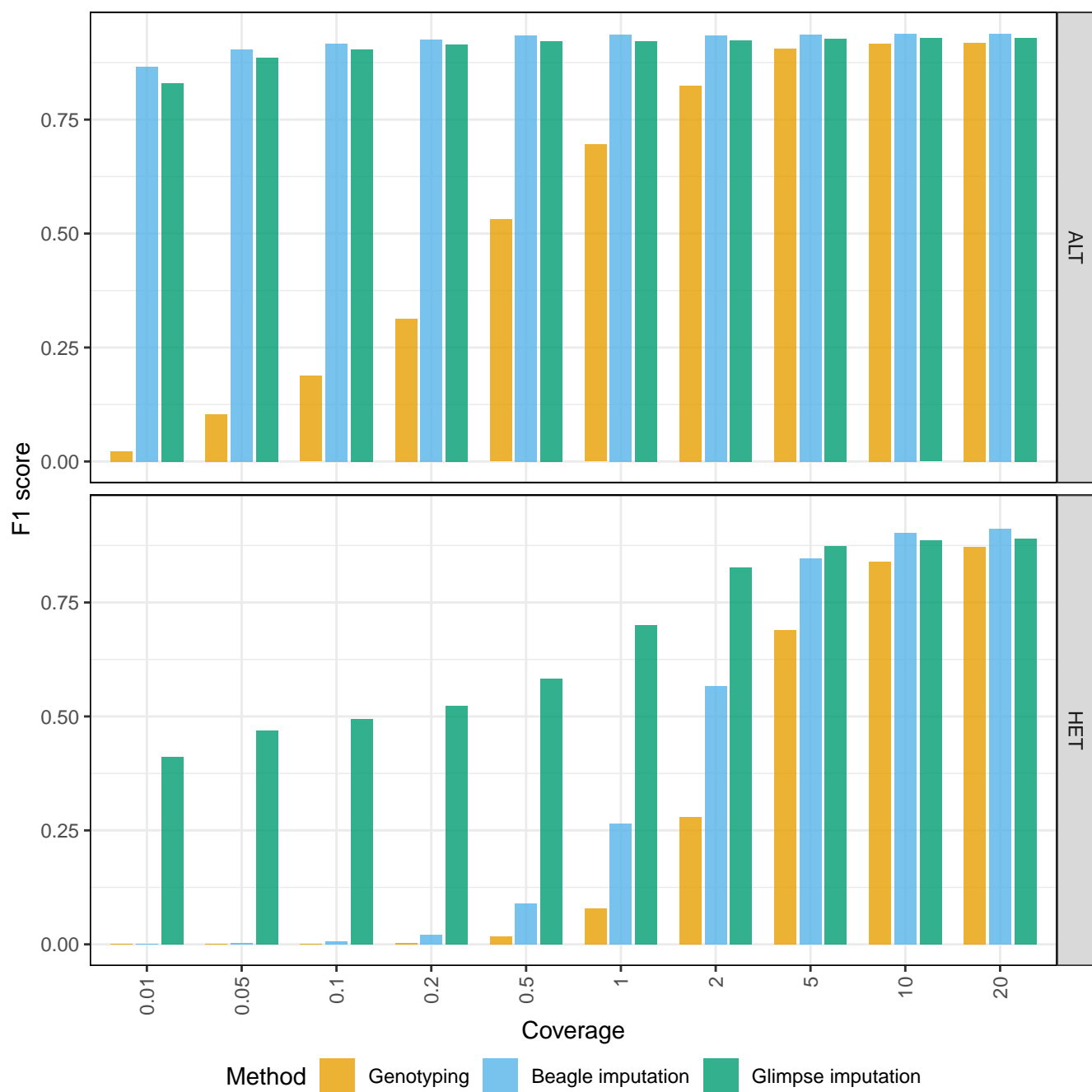

Supplement: Supplement 1 [file Supplemental_Code.zip › imputefirst-main/plots_data_scripts/upstream_plots/Figure_2.pdf]

% matching 200bp window

1 -- 5 (n = 17564978)

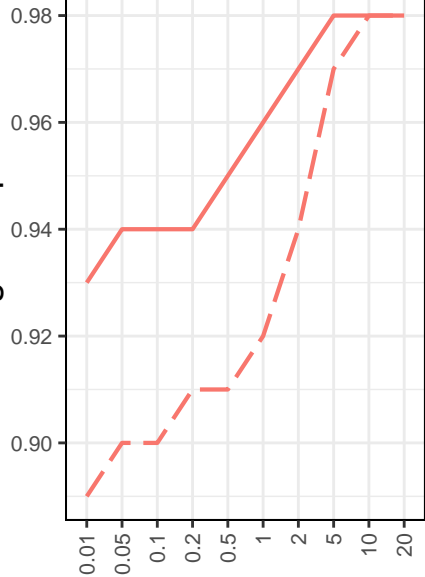

6 -- 10 (n = 1427164)

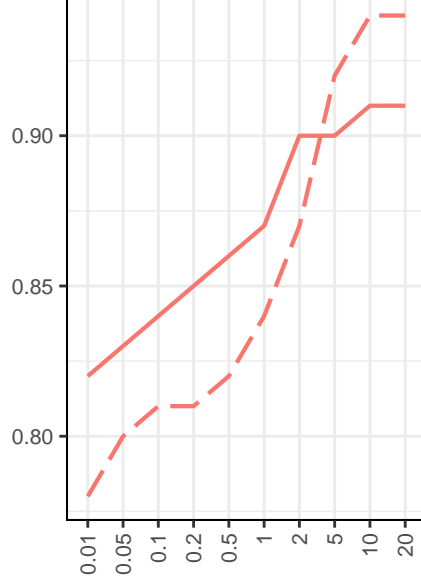

11+ (n = 371836)

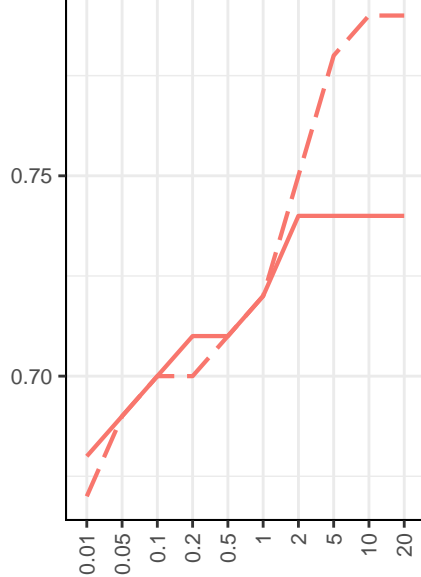

--- Bowtie 2 + BCFtools + Beagle    — Bowtie 2 + BCFtools + Glimpse

Supplement: Supplement 1 [file Supplemental_Code.zip › imputefirst-main/plots_data_scripts/upstream_plots/Figure_3.pdf]

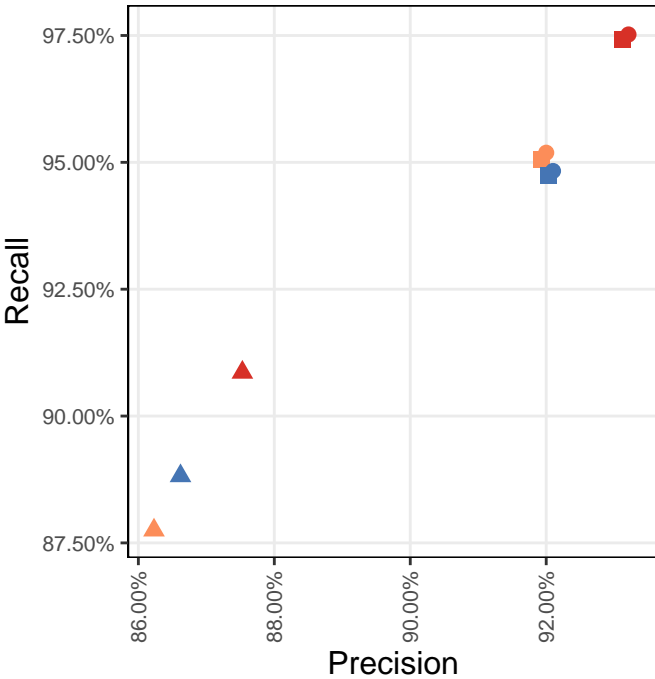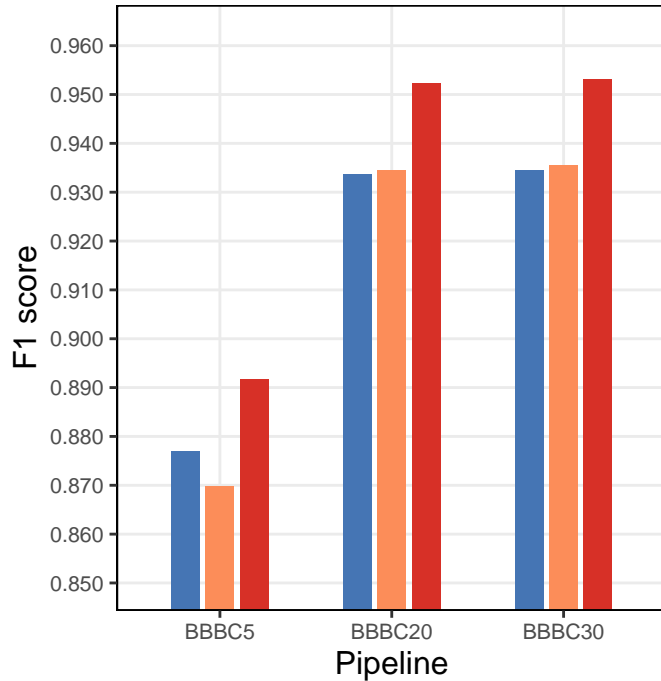

Pipeline ▲ BBBC5 ■ BBBC20 ● BBBC30

Panel ● HPRC\_filtered ● HGSVC2 ● HGSVC3

Supplement: Supplement 1 [file Supplemental_Code.zip › imputefirst-main/plots_data_scripts/upstream_plots/Figure_4.pdf]

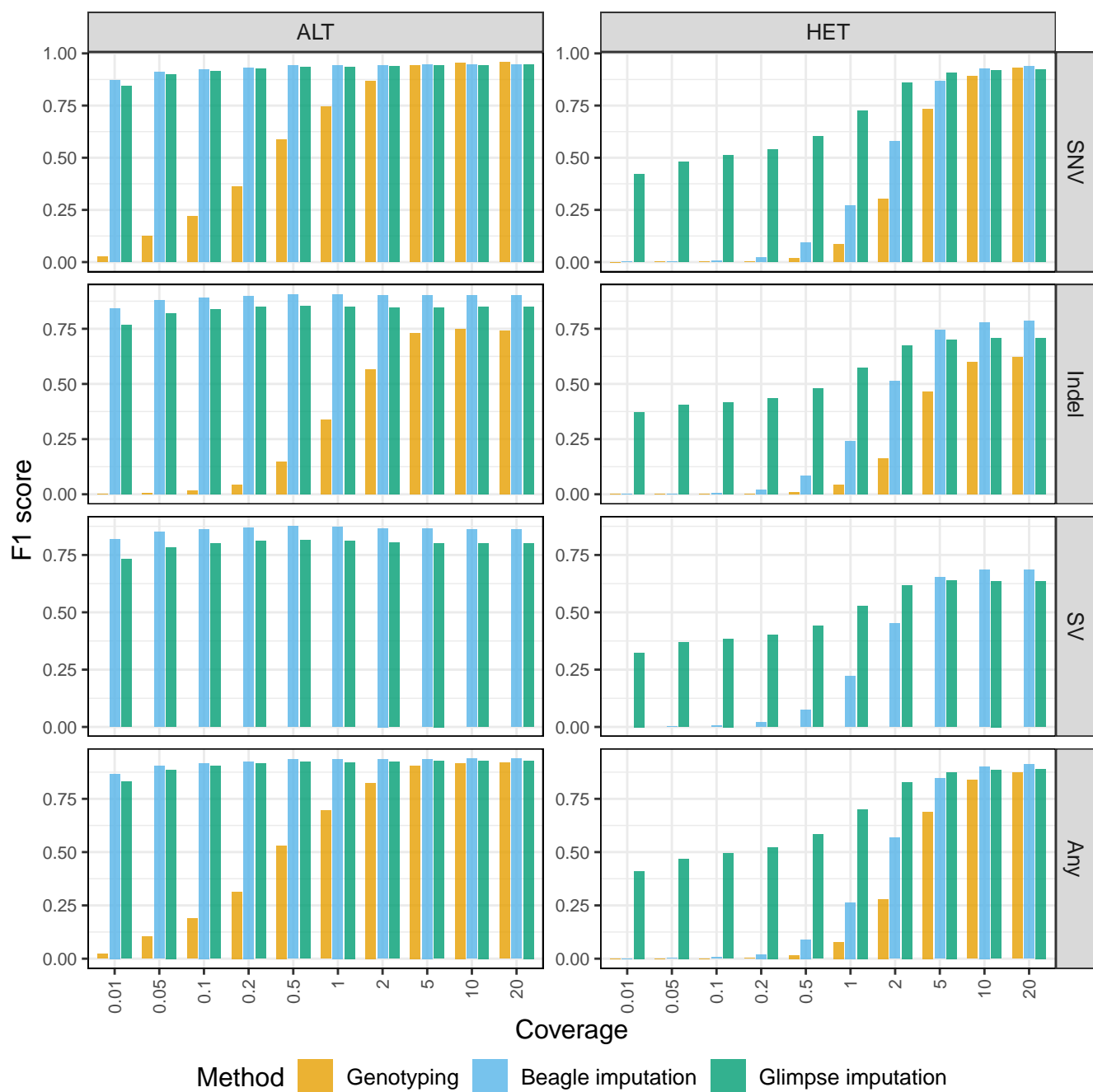

Supplement: Supplement 1 [file Supplemental_Code.zip › imputefirst-main/plots_data_scripts/upstream_plots/Figure_S1.pdf]

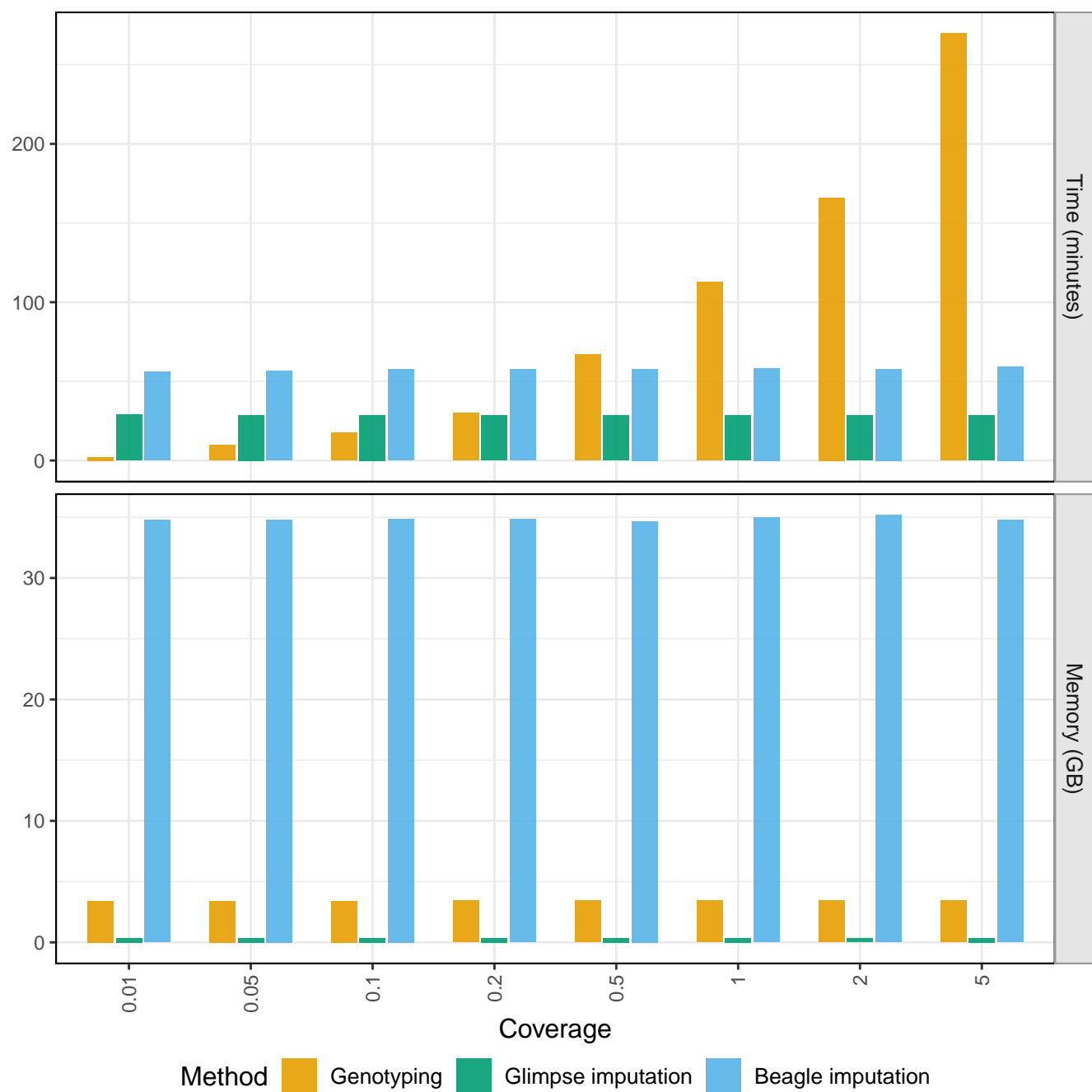

Supplement: Supplement 1 [file Supplemental_Code.zip › imputefirst-main/plots_data_scripts/upstream_plots/Figure_S2.pdf]

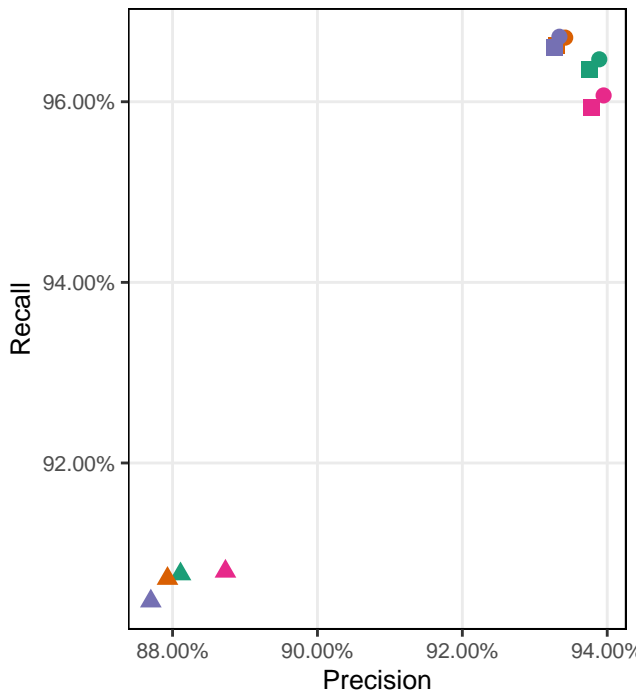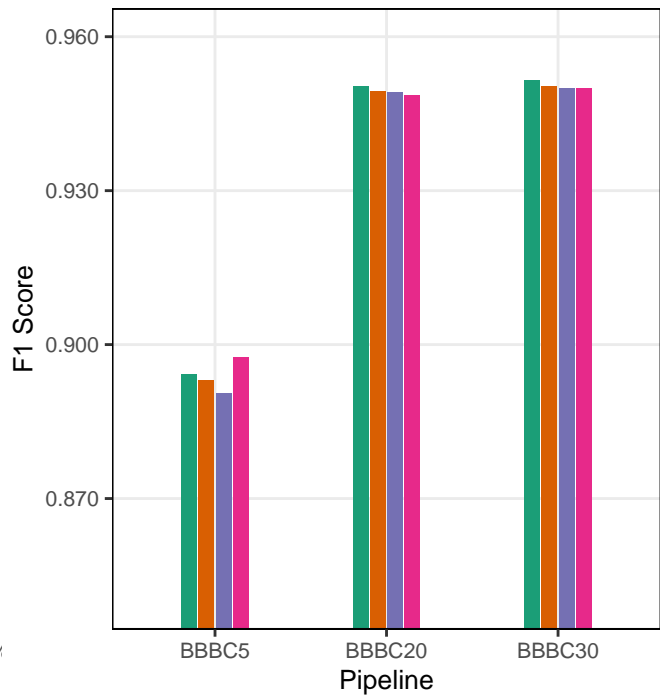

Sample ● HG001 ● HG003 ● HG004 ● HG005 Pipeline ▲ BBBC5 ■ BBBC20 ● BBBC30

Supplement: Supplement 1 [file Supplemental_Code.zip › imputefirst-main/plots_data_scripts/upstream_plots/Figure_S3.pdf]
