# Supplementary material for: Minimizing reference bias with an imputed personalized reference
Source: Genome Res. 2026 Apr;36(4):740–53. doi: 10.1101/gr.280989.125 (PMC13138014; doi:10.1101/gr.280989.125)
Supplement: Supplement 2 [file Supplemental_Material.pdf]

Supplementary Tables

Table S1: Variant calling performance metrics for different reference panels and pipelines. BBBCn indicates pipeline configuration: **B**owtie 2 + **B**CFtools + **B**eagle at **C**overage n (n = 5, 20, 30).

Group	Sample	Panel	Pipeline	FP	FN	Precision	Sensitivity	F-measure
Panel Comparison	HG002	HGSVC2	BBBC5	539,523	476,511	0.8623	0.8775	0.8697
			BBBC20	317,373	192,261	0.9193	0.9505	0.9345
			BBBC30	314,685	186,980	0.9200	0.9519	0.9355
		HGSVC3	BBBC5	505,627	355,348	0.8753	0.9086	0.8916
			BBBC20	280,622	100,043	0.9312	0.9742	0.9522
			BBBC30	277,354	96,345	0.9321	0.9752	0.9531
		HPRC_filtered*	BBBC5	534,689	434,932	0.8662	0.8882	0.8770
			BBBC20	318,868	204,566	0.9204	0.9474	0.9337
			BBBC30	316,533	200,963	0.9210	0.9483	0.9344
Sample Comparison	HG001	HGSVC3	BBBC5	458,402	343,610	0.8811	0.9077	0.8942
			BBBC20	240,538	135,422	0.9376	0.9636	0.9504
			BBBC30	235,497	131,333	0.9389	0.9647	0.9516
	HG003	HGSVC3	BBBC5	479,452	355,686	0.8793	0.9072	0.8930
			BBBC20	267,619	129,695	0.9330	0.9662	0.9493
			BBBC30	262,768	125,880	0.9342	0.9671	0.9504
	HG004	HGSVC3	BBBC5	491,967	367,682	0.8770	0.9047	0.8906
			BBBC20	270,502	130,971	0.9327	0.9660	0.9491
			BBBC30	268,129	126,518	0.9334	0.9672	0.9500
	HG005	HGSVC3	BBBC5	427,052	339,859	0.8873	0.9080	0.8975
			BBBC20	235,957	149,809	0.9378	0.9594	0.9485
			BBBC30	229,327	145,144	0.9395	0.9607	0.9500

*HPRC_filtered panel uses the bi-allelic file `cactus_filtered_ids_biallelic.vcf.gz`, generated from `cactus_filtered_ids.vcf.gz` (derived from `hprc-v1.0-mc-grch38.vcf.gz`) by decomposing nested variants into single-ID records using the PanGenie conversion script `convert-to-biallelic.py` as documented in the associated Zenodo record description link.

Table S2: Window accuracy for HG002 (HGSVC3 panel) using Bowtie 2 and BWA-MEM as the personalization aligner. Accuracy was computed at downsampling coverages of 1×, 2×, 5×, and 20×, stratified by variant density (number of polymorphic sites per 200 bp window; 1–5, 6–10, 11+). Results indicate only modest differences between aligners across all coverage levels.

Coverage	Personalization aligner	1–5	6–10	11+
1×	Bowtie 2	0.94	0.87	0.75
	BWA-MEM	0.94	0.87	0.75
2×	Bowtie 2	0.95	0.90	0.78
	BWA-MEM	0.95	0.90	0.77
5×	Bowtie 2	0.98	0.95	0.82
	BWA-MEM	0.98	0.95	0.82
20×	Bowtie 2	0.99	0.96	0.83
	BWA-MEM	0.99	0.96	0.83

Table S3: Computational overhead for the personalization step on HG002 across three impute-first configurations (`Imputefirst_c1`, `Imputefirst_c5`, `Imputefirst_c20`). We report wall-clock elapsed time in seconds, peak memory in MB, and raw CPU time in seconds. All pipelines used 32 threads (except `bcftools consensus` for fasta_construction, which is single-threaded).

Pipeline	Step	Elapsed (s)	Memory (MB)	CPU_time (s)
`Imputefirst_c1`: Personalization	bowtie2_align	462.38	7,894.06	10,309.51
	bcftools_call	2,545.17	17,963.97	3,161.32
	beagle_imputation	301.65	42,492.53	3,865.91
	fasta_construction	252.52	6,608.10	215.23
	Total	3,561.72	42,492.53	17,551.97
`Imputefirst_c5`: Personalization	bowtie2_align	2,462.64	7,962.16	59,278.57
	bcftools_call	5,194.06	17,964.09	6,137.28
	beagle_imputation	563.98	42,684.21	3,660.77
	fasta_construction	80.29	4,858.10	39.44
	Total	8,299.00	42,684.21	69,115.99
`Imputefirst_c20`: Personalization	bowtie2_align	10,592.43	7,962.19	266,266.71
	bcftools_call	12,409.79	17,964.14	13,482.56
	beagle_imputation	738.93	42,876.21	8,061.07
	fasta_construction	77.57	4,991.67	44.90
	Total	23,718.70	42,876.21	287,855.24

Table S4: Computational overhead for the impute-first workflows on HG002 across three coverage configurations. We report wall-clock elapsed time in minutes, peak memory in GB, and raw CPU time in seconds. For reference, (P) = Personalization, (D) = Downstream, (P+D) = Overall. The last column reports the fraction of total CPU time spent in personalization. All pipelines used 32 threads (except `bcftools consensus` for fasta_construction and `bwa index`, which are single-threaded).

**Workflow**	**Step**	**Time (min)**	**Mem (GB)**	**CPU time (s)**	**P CPU %**
Giraffe (Imputefirst_c1)	(P)	59.36	42.49	17,551.97	
	(D)	274.22	126.57	370,473.32	
	(P+D)	333.58	126.57	388,025.29	4.5
Giraffe (Imputefirst_c5)	(P)	148.80	43.13	69,115.99	
	(D)	275.62	126.36	371,062.92	
	(P+D)	424.42	126.36	440,178.91	15.7
Giraffe (Imputefirst_c20)	(P)	405.22	42.88	287,855.24	
	(D)	273.29	126.46	369,382.82	
	(P+D)	678.51	126.46	657,238.06	43.8
LevioSAM2 (Imputefirst_c1)	(P)	59.36	42.49	17,551.97	
	(D)	1,694.27	37.06	1,486,904.53	
	(P+D)	1,753.63	42.49	1,504,456.50	1.2
LevioSAM2 (Imputefirst_c5)	(P)	148.80	43.13	69,115.99	
	(D)	1,632.83	37.06	1,374,587.63	
	(P+D)	1,781.63	43.13	1,443,703.62	4.8
LevioSAM2 (Imputefirst_c20)	(P)	405.22	42.88	287,855.24	
	(D)	1,619.14	37.06	1,375,536.57	
	(P+D)	2,024.36	42.88	1,663,391.81	17.3

Table S5: Variant calling performance metrics for HG001 real donor reads (DeepVariant v1.5.0), stratified by SNVs, indels across different pipelines within GIAB HG001 high-confidence regions.

Type	Pipeline	TRUTH.TP	QUERY.FP	TRUTH.FN	METRIC.Recall	METRIC.Precision
INDEL	BWA-MEM	464,619	1,452	3,083	0.993408	0.997001
	Giraffe(HPRC_pangenome)	465,276	1,741	2,427	0.9948110000000001	0.996413
	Giraffe(Imputefirst_c20)	465,643	1,533	2,058	0.9956	0.996841
	Giraffe(Imputefirst_c5)	465,631	1,533	2,070	0.995574	0.996841
	Giraffe(benchmark)	466,022	1,440	1,680	0.996408	0.997035
	Giraffe(diploid)	465,492	1,708	2,210	0.995275	0.996481
	Giraffe(diploid_reported)	465,407	1,744	2,296	0.995091	0.996267
	LevioSAM2(Imputefirst_c20)	465,651	1,497	2,051	0.995615	0.996916
	LevioSAM2(Imputefirst_c5)	465,637	1,522	2,065	0.995585	0.996864
	LevioSAM2(benchmark)	465,710	1,419	1,993	0.995739	0.997076
SNP	BWA-MEM	3,237,737	4,385	16,649	0.994884	0.998648
	Giraffe(HPRC_pangenome)	3,246,773	4,876	7,613	0.997661	0.998501
	Giraffe(Imputefirst_c20)	3,246,931	3,734	7,455	0.997709	0.998852
	Giraffe(Imputefirst_c5)	3,246,713	3,743	7,673	0.997642	0.998849
	Giraffe(benchmark)	3,249,069	4,096	5,317	0.998366	0.998741
	Giraffe(diploid)	3,246,566	5,768	7,820	0.997597	0.998227
	Giraffe(diploid_reported)	3,247,868	4,484	6,518	0.997997	0.998621
	LevioSAM2(Imputefirst_c20)	3,247,652	4,568	6,734	0.997931	0.998596
	LevioSAM2(Imputefirst_c5)	3,247,478	5,115	6,908	0.997877	0.998428
	LevioSAM2(benchmark)	3,247,675	4,405	6,711	0.997938	0.998646

Table S6: Variant calling performance metrics for HG002 real donor reads (DeepVariant v1.5.0), stratified by SNVs, indels across different pipelines within GIAB HG002 high-confidence regions.

Type	Pipeline	TRUTH.TP	QUERY.FP	TRUTH.FN	METRIC.Recall	METRIC.Precision
INDEL	BWA-MEM	521,652	1,407	3,817	0.992736	0.997419
	Giraffe(HPRC_pangenome)	522,311	1,786	3,158	0.993990	0.996732
	Giraffe(Imputefirst_c20)	522,981	1,448	2,488	0.995265	0.997351
	Giraffe(Imputefirst_c5)	522,940	1,422	2,529	0.995187	0.997398
	Giraffe(benchmark)	523,375	1,396	2,094	0.996015	0.997448
	Giraffe(diploid)	522,629	1,691	2,840	0.994595	0.996906
	Giraffe(diploid_reported)	522,539	1,760	2,930	0.994424	0.996643
	LevioSAM2(Imputefirst_c20)	522,961	1,397	2,508	0.995227	0.997444
	LevioSAM2(Imputefirst_c5)	522,953	1,426	2,516	0.995212	0.997391
	LevioSAM2(benchmark)	523,048	1,375	2,421	0.995393	0.997485
SNP	BWA-MEM	3,343,893	3,414	21,234	0.99369	0.998981
	Giraffe(HPRC_pangenome)	3,353,297	4,750	11,830	0.9964850	0.998586
	Giraffe(Imputefirst_c20)	3,353,942	3,844	11,185	0.996676	0.998856
	Giraffe(Imputefirst_c5)	3,353,645	3,703	11,482	0.996588	0.998898
	Giraffe(benchmark)	3,357,089	4,880	8,038	0.997611	0.998549
	Giraffe(diploid)	3,352,376	6,213	12,751	0.996211	0.998151
	Giraffe(diploid_reported)	3,354,666	4,329	10,461	0.996891	0.998711
	LevioSAM2(Imputefirst_c20)	3,355,300	4,569	9,827	0.99708	0.998641
	LevioSAM2(Imputefirst_c5)	3,355,075	4,870	10,052	0.997013	0.998551
	LevioSAM2(benchmark)	3,356,108	4,509	9,019	0.99732	0.998659

Table S7: Variant calling performance metrics for HG003 real donor reads (DeepVariant v1.5.0), stratified by SNVs, indels across different pipelines within GIAB HG003 high-confidence regions.

Type	Pipeline	TRUTH.TP	QUERY.FP	TRUTH.FN	METRIC.Recall	METRIC.Precision
INDEL	BWA-MEM	501,072	1,505	3,429	0.993203	0.997126
	Giraffe(HPRC_pangenome)	501,757	1,792	2,744	0.994561	0.996586
	Giraffe(Imputefirst_c20)	502,264	1,581	2,237	0.995566	0.996989
	Giraffe(Imputefirst_c5)	502,249	1,579	2,252	0.995536	0.996992
	Giraffe(benchmark)	502,622	1,564	1,879	0.996276	0.997023
	Giraffe(diploid)	501,958	1,829	2,543	0.994959	0.996516
	Giraffe(diploid_reported)	501,934	1,789	2,567	0.994912	0.996448
	LevioSAM2(Imputefirst_c20)	502,165	1,604	2,336	0.99537	0.996945
	LevioSAM2(Imputefirst_c5)	502,157	1,629	2,344	0.995354	0.996897
	LevioSAM2(benchmark)	502,261	1,576	2,241	0.995558	0.996999
SNP	BWA-MEM	3,305,557	4,676	21,939	0.993407	0.998588
	Giraffe(HPRC_pangenome)	3,315,376	5,758	12,120	0.996358	0.998267
	Giraffe(Imputefirst_c20)	3,315,809	4,767	11,687	0.996488	0.998565
	Giraffe(Imputefirst_c5)	3,315,782	4,830	11,714	0.99648	0.998546
	Giraffe(benchmark)	3,319,095	6,049	8,401	0.997475	0.998182
	Giraffe(diploid)	3,314,354	7,825	13,142	0.99605	0.997646
	Giraffe(diploid_reported)	3,316,604	5,293	10,892	0.996727	0.998407
	LevioSAM2(Imputefirst_c20)	3,317,158	5,725	10,338	0.996893	0.998278
	LevioSAM2(Imputefirst_c5)	3,316,852	6,434	10,644	0.996801	0.998065
	LevioSAM2(benchmark)	3,318,133	5,785	9,363	0.997186	0.99826

Table S8: Variant calling performance metrics for HG004 real donor reads (Deepvariant v1.5.0), stratified by SNVs, indels across different pipelines within GIAB HG004 high-confidence regions.

Type	Pipeline	TRUTH.TP	QUERY.FP	TRUTH.FN	METRIC.Recall	METRIC.Precision
INDEL	BWA-MEM	506,986	1,455	3,533	0.99308	0.997257
	Giraffe(HPRC_pangenome)	507,652	1,729	2,867	0.994384	0.996747
	Giraffe(Imputefirst_c20)	508,192	1,521	2,327	0.995442	0.997139
	Giraffe(Imputefirst_c5)	508,186	1,512	2,333	0.99543	0.997156
	Giraffe(benchmark)	508,606	1,445	1,913	0.996253	0.997285
	Giraffe(diploid)	507,856	1,758	2,663	0.994784	0.996693
	Giraffe(diploid_reported)	507,851	1,742	2,668	0.994774	0.996582
	LevioSAM2(Imputefirst_c20)	508,075	1,543	2,444	0.995213	0.997098
	LevioSAM2(Imputefirst_c5)	508,067	1,577	2,452	0.995197	0.997034
	LevioSAM2(benchmark)	508,172	1,484	2,347	0.995403	0.997209
SNP	BWA-MEM	3,323,615	4,128	22,995	0.993129	0.99876
	Giraffe(HPRC_pangenome)	3,333,187	5,231	13,423	0.995989	0.998434
	Giraffe(Imputefirst_c20)	3,334,254	4,778	12,356	0.996308	0.99857
	Giraffe(Imputefirst_c5)	3,334,014	4,804	12,596	0.996236	0.998562
	Giraffe(benchmark)	3,337,400	5,721	9,210	0.997248	0.998289
	Giraffe(diploid)	3,331,770	7,640	14,840	0.995566	0.997713
	Giraffe(diploid_reported)	3,334,341	5,099	12,269	0.996334	0.998473
	LevioSAM2(Imputefirst_c20)	3,335,336	5,460	11,274	0.996631	0.998366
	LevioSAM2(Imputefirst_c5)	3,334,881	6,014	11,729	0.996495	0.998201
	LevioSAM2(benchmark)	3,336,137	5,295	10,473	0.996871	0.998416

Table S9: Variant calling performance metrics for HG005 real donor reads (Deepvariant v1.5.0), stratified by SNVs, indels across different pipelines within GIAB HG005 high-confidence regions.

Type	Pipeline	TRUTH.TP	QUERY.FP	TRUTH.FN	METRIC.Recall	METRIC.Precision
INDEL	BWA-MEM	414,074	892	2,703	0.993515	0.997917
	Giraffe(HPRC_pangenome)	414,804	1,091	1,972	0.995268	0.997459
	Giraffe(Imputefirst_c20)	415,165	953	1,611	0.996135	0.997781
	Giraffe(Imputefirst_c5)	415,170	944	1,606	0.996147	0.997802
	Giraffe(benchmark)	415,499	939	1,277	0.996936	0.997815
	Giraffe(diploid)	414,848	1,122	1,928	0.995374	0.997387
	Giraffe(diploid_reported)	414,909	1,109	1,867	0.99552	0.997334
	LevioSAM2(Imputefirst_c20)	415,121	1,005	1,655	0.996029	0.99766
	LevioSAM2(Imputefirst_c5)	415,093	1,024	1,683	0.995962	0.997616
	LevioSAM2(benchmark)	415,200	946	1,576	0.996219	0.997798
SNP	BWA-MEM	3,252,931	3,897	22,700	0.99307	0.998804
	Giraffe(HPRC_pangenome)	3,262,890	4,663	12,741	0.99611	0.998574
	Giraffe(Imputefirst_c20)	3,263,138	4,012	12,493	0.996186	0.998772
	Giraffe(Imputefirst_c5)	3,263,129	3,801	12,502	0.996183	0.998837
	Giraffe(benchmark)	3,266,359	4,739	9,272	0.997169	0.998552
	Giraffe(diploid)	3,260,721	6,003	14,910	0.995448	0.998163
	Giraffe(diploid_reported)	3,263,672	4,384	11,959	0.996349	0.998659
	LevioSAM2(Imputefirst_c20)	3,264,287	5,214	11,344	0.996537	0.998406
	LevioSAM2(Imputefirst_c5)	3,264,160	5,127	11,471	0.996498	0.998432
	LevioSAM2(benchmark)	3,265,544	4,936	10,087	0.996921	0.998491

Table S10: Variant calling performance metrics for HG002 T2TQ100 v1.0 ground truth, evaluated within T2TQ100 v1.0 declared high-confidence regions (DeepVariant v1.5.0).

Pipeline	TRUTH.TP	QUERY.FP	TRUTH.FN	Total Errors	METRIC.Recall	METRIC.Precision	METRIC.F1
BWA-MEM	4,529,709	37,600	124,852	162,452	0.973176	0.991722	0.982362
Giraffe(HPRC_pangenome)	4,548,830	40,030	105,732	145,762	0.977284	0.991234	0.98421
Giraffe(Imputefirst_c20)	4,548,833	37,256	105,735	142,991	0.977284	0.99183	0.984503
Giraffe(Imputefirst_c5)	4,548,883	36,839	105,677	142,516	0.977296	0.991921	0.984554
Giraffe(benchmark)	4,550,958	37,645	103,596	141,241	0.977743	0.991748	0.984696
Giraffe(diploid)	4,555,631	37,512	98,928	136,440	0.978746	0.991787	0.985223
LevioSAM2(Imputefirst_c20)	4,559,611	39,855	94,926	134,781	0.979606	0.991285	0.985411
LevioSAM2(Imputefirst_c5)	4,559,275	39,770	95,275	135,045	0.979531	0.991303	0.985382
LevioSAM2(benchmark)	4,554,369	38,724	100,182	138,906	0.978477	0.99152	0.984955

Table S11: Variant calling performance metrics for HG002 GIAB CMRG ground truth, evaluated within CMRG high-confidence regions (DeepVariant v1.5.0).

Pipeline	TRUTH.TP	QUERY.FP	TRUTH.FN	Total Errors	METRIC.Recall	METRIC.Precision	METRIC.F1
BWA-MEM	20,322	286	904	1,190	0.957411	0.986252	0.971617
Giraffe(HPRC_pangenome)	20,657	256	575	831	0.972918	0.987868	0.980336
Giraffe(Imputefirst_c20)	20,212	306	1,014	1,320	0.952228	0.98522	0.968443
Giraffe(Imputefirst_c5)	20,182	300	1,044	1,344	0.950815	0.985489	0.967842
Giraffe(benchmark)	20,456	239	770	1,009	0.963724	0.988547	0.975977
Giraffe(diploid)	20,536	276	690	966	0.967493	0.986853	0.977077
LevioSAM2(Imputefirst_c20)	20,621	295	605	900	0.971497	0.986028	0.978708
LevioSAM2(Imputefirst_c5)	20,612	298	614	912	0.971073	0.985881	0.978421
LevioSAM2(benchmark)	20,527	263	699	962	0.967069	0.987465	0.977161

Table S12: Variant calling performance metrics for HG002 across different genomic regions (DeepVariant v1.5.0).

Region	Pipeline	TP	FP	FN	Total Errors	Recall	Precision	F1
**Tandem Repeats (TR)**	BWA-MEM	193,329	1,116	2,145	3,261	0.989027	0.994605	0.991808
	Giraffe(HPRC_pangenome)	193,137	1,535	2,338	3,873	0.988039	0.992597	0.990313
	Giraffe(Imputefirst_c20)	193,870	1,048	1,604	2,652	0.991794	0.994947	0.993368
	Giraffe(Imputefirst_c5)	193,854	1,027	1,620	2,647	0.991712	0.995047	0.993377
	Giraffe(benchmark)	194,147	918	1,327	2,245	0.993211	0.995577	0.994393
	Giraffe(diploid)	193,527	1,306	1,947	3,253	0.990040	0.993701	0.991867
	Leviosam2(Imputefirst_c20)	193,917	1,027	1,557	2,584	0.992035	0.995049	0.993540
	Leviosam2(Imputefirst_c5)	193,924	1,031	1,550	2,581	0.992071	0.995030	0.993548
	Leviosam2(benchmark)	193,975	984	1,499	2,483	0.992331	0.995257	0.993792
**Homopolymer Repeats (HP)**	BWA-MEM	182,770	187	465	652	0.997462	0.998986	0.998224
	Giraffe(HPRC_pangenome)	182,897	241	339	580	0.998150	0.998696	0.998423
	Giraffe(Imputefirst_c20)	182,911	183	324	507	0.998232	0.999009	0.998620
	Giraffe(Imputefirst_c5)	182,907	188	328	516	0.998210	0.998982	0.998596
	Giraffe(benchmark)	182,953	183	282	465	0.998461	0.999009	0.998735
	Giraffe(diploid)	182,898	263	338	601	0.998155	0.998576	0.998366
	Leviosam2(Imputefirst_c20)	182,927	192	309	501	0.998314	0.998960	0.998637
	Leviosam2(Imputefirst_c5)	182,920	202	316	518	0.998275	0.998906	0.998591
	Leviosam2(benchmark)	182,927	182	308	490	0.998319	0.999014	0.998667
**Low Mappability (LowMap)**	BWA-MEM	182,531	2,960	20,522	23,482	0.898933	0.984053	0.939569
	Giraffe(HPRC_pangenome)	192,610	4,139	10,444	14,583	0.948565	0.978977	0.963531
	Giraffe(Imputefirst_c20)	192,924	3,404	10,128	13,532	0.950121	0.982672	0.966123
	Giraffe(Imputefirst_c5)	192,605	3,257	10,447	13,704	0.948550	0.983381	0.965651
	Giraffe(benchmark)	195,519	4,540	7,533	12,073	0.962901	0.977320	0.970057
	Giraffe(diploid)	191,895	5,288	11,158	16,446	0.945049	0.973198	0.958917
	Leviosam2(Imputefirst_c20)	194,084	4,173	8,969	13,142	0.955829	0.978964	0.967258
	Leviosam2(Imputefirst_c5)	193,838	4,480	9,215	13,695	0.954618	0.977423	0.965886
	Leviosam2(benchmark)	194,891	4,172	8,163	12,335	0.959799	0.979054	0.969331
**Segmental Duplications (SegDup)**	BWA-MEM	102,511	2,016	11,027	13,043	0.902878	0.980752	0.940205
	Giraffe(HPRC_pangenome)	105,304	3,489	8,234	11,723	0.927478	0.967996	0.947304
	Giraffe(Imputefirst_c20)	105,938	2,537	7,600	10,137	0.933062	0.976657	0.954362
	Giraffe(Imputefirst_c5)	105,764	2,348	7,774	10,122	0.931530	0.978323	0.954353
	Giraffe(benchmark)	108,314	3,492	5,224	8,716	0.953989	0.968823	0.961349
	Giraffe(diploid)	104,453	4,880	9,085	13,965	0.919983	0.955450	0.937381
	Leviosam2(Imputefirst_c20)	107,266	3,441	6,272	9,713	0.944759	0.968977	0.956714
	Leviosam2(Imputefirst_c5)	107,121	3,679	6,417	10,096	0.943481	0.966858	0.955027
	Leviosam2(benchmark)	108,236	3,391	5,302	8,693	0.953302	0.969678	0.961420
**Extreme GC Content (GC)**	BWA-MEM	262,630	406	1,448	1,854	0.994517	0.998467	0.996488
	Giraffe(HPRC_pangenome)	262,801	653	1,277	1,930	0.995164	0.997538	0.996350
	Giraffe(Imputefirst_c20)	263,066	466	1,012	1,478	0.996168	0.998244	0.997205
	Giraffe(Imputefirst_c5)	263,022	468	1,056	1,524	0.996001	0.998236	0.997117
	Giraffe(benchmark)	263,335	466	743	1,209	0.997186	0.998245	0.997716
	Giraffe(diploid)	262,810	652	1,268	1,920	0.995198	0.997542	0.996369
	Leviosam2(Imputefirst_c20)	263,234	493	844	1,337	0.996804	0.998143	0.997473
	Leviosam2(Imputefirst_c5)	263,189	524	889	1,413	0.996634	0.998026	0.997329
	Leviosam2(benchmark)	263,268	457	810	1,267	0.996933	0.998279	0.997605
**Difficult Regions (Difficult)**	BWA-MEM	60,439	1,547	5,153	6,700	0.921439	0.975262	0.947587
	Giraffe(HPRC_pangenome)	62,141	1,269	3,452	4,721	0.947372	0.980175	0.963495
	Giraffe(Imputefirst_c20)	62,336	1,269	3,256	4,525	0.950360	0.980210	0.965054
	Giraffe(Imputefirst_c5)	62,290	1,254	3,302	4,556	0.949658	0.980423	0.964795
	Giraffe(benchmark)	63,173	1,739	2,419	4,158	0.963121	0.973422	0.968244
	Giraffe(diploid)	61,981	1,672	3,611	5,283	0.944948	0.973954	0.959232
	Leviosam2(Imputefirst_c20)	63,280	1,434	2,312	3,746	0.964752	0.978022	0.971342
	Leviosam2(Imputefirst_c5)	63,237	1,587	2,355	3,942	0.964096	0.975717	0.969872
	Leviosam2(benchmark)	63,466	1,493	2,126	3,619	0.967588	0.977201	0.972371

Table S13: HG002 variant-calling performance across GIAB v3.1 stratified genomic regions outside GIAB high-confidence regions. (The GIAB high-confidence regions covers $\sim 82\%$ of the GRCh38 fasta for HG001–HG005 samples (HG001: 81.35%, HG002: 82.32%, HG003: 81.88%, HG004: 81.74%, and HG005: 81.07%.)

Pipeline	TP	FP	FN	Total Errors	Precision	Recall	F1
BWA-MEM	4,010,469	697,094	37,779	734,873	0.852895	0.990668	0.916633
Giraffe(HPRC_pangenome)	4,022,618	747,598	25,627	773,225	0.844326	0.993670	0.912931
Giraffe(Imputefirst_c5)	4,024,784	733,176	23,461	756,637	0.846911	0.994205	0.914666
Giraffe(Imputefirst_c20)	4,025,182	733,172	23,066	756,238	0.846925	0.994302	0.914715
Giraffe(diploid)	4,022,972	779,471	25,273	804,744	0.838745	0.993757	0.909695
Leviosam2(Imputefirst_c5)	4,026,059	771,925	22,186	794,111	0.840158	0.994520	0.910845
Leviosam2(Imputefirst_c20)	4,026,388	773,059	21,857	794,916	0.839972	0.994601	0.910770

Table S14: Variant calling performance metrics for HG002 real donor reads (DeepVariant v1.5.0), aggregated overall SNVs and indels within GIAB HG002 high-confidence regions. *Note: The HGSVC3 (no SVs) panel is designed to isolate the effect of large structural variants on downstream analysis, so we regenerated the personalized VCF for HG002 using the HGSVC3 panel after removing all structural variants with length greater than 50 bp that were present in the panel. This removed 176,231 SVs in total. All other steps were identical, enabling a direct comparison between SV-inclusive and no-SV panel based pipelines.*

Pipeline	TP	FP	FN	Recall	Precision	F1
BWA-MEM	3,865,545	4,821	25,051	0.993561	0.998762	0.996155
Giraffe(HPRC_pangenome)	3,875,608	6,536	14,988	0.996148	0.998327	0.997236
Giraffe(diploid)	3,875,005	7,904	15,591	0.995993	0.997977	0.996984
Giraffe(diploid_reported)	3,877,205	6,089	13,391	0.996558	0.998432	0.997494
Leviosam2(benchmark)	3,879,156	5,884	11,440	0.997060	0.998495	0.997777
Giraffe(benchmark)	3,880,464	6,276	10,132	0.997396	0.998395	0.997895
**HGSVC3 panel**						
Giraffe(Imputefirst_c1)	3,875,483	5,104	15,113	0.996116	0.998693	0.997402
Giraffe(Imputefirst_c2)	3,875,923	5,061	14,673	0.996229	0.998704	0.997465
Giraffe(Imputefirst_c5)	3,876,585	5,125	14,011	0.996399	0.998688	0.997542
Giraffe(Imputefirst_c20)	3,876,923	5,292	13,673	0.996486	0.998645	0.997565
Leviosam2(Imputefirst_c0.5)	3,875,650	10,001	14,946	0.996158	0.997442	0.996800
Leviosam2(Imputefirst_c1)	3,876,129	9,614	14,467	0.996282	0.997541	0.996911
Leviosam2(Imputefirst_c2)	3,876,835	8,137	13,761	0.996463	0.997918	0.997190
Leviosam2(Imputefirst_c5)	3,878,028	6,296	12,568	0.996770	0.998389	0.997579
Leviosam2(Imputefirst_c10)	3,878,074	6,013	12,522	0.996781	0.998461	0.997620
Leviosam2(Imputefirst_c20)	3,878,261	5,966	12,335	0.996830	0.998473	0.997652
**HGSVC3 (no SVs) panel**						
Giraffe(Imputefirst_c5)	3,876,570	5,304	14,026	0.996395	0.998642	0.997517
Giraffe(Imputefirst_c20)	3,876,897	5,479	13,699	0.996479	0.998597	0.997536
Leviosam2(Imputefirst_c5)	3,877,946	6,295	12,650	0.996749	0.998389	0.997568
Leviosam2(Imputefirst_c20)	3,878,118	6,002	12,478	0.996793	0.998464	0.997628
**HPRC_filtered panel***						
Giraffe(Imputefirst_c5)	3,875,863	5,672	14,733	0.996213	0.998548	0.997379
Giraffe(Imputefirst_c20)	3,876,191	5,719	14,405	0.996297	0.998536	0.997415
Leviosam2(Imputefirst_c5)	3,877,580	7,050	13,016	0.996654	0.998196	0.997425
Leviosam2(Imputefirst_c20)	3,877,850	6,635	12,746	0.996724	0.998302	0.997513

*HPRC_filtered panel uses the bi-allelic file `cactus_filtered_ids_biallelic.vcf.gz`, generated from `cactus_filtered_ids.vcf.gz` (derived from `hprc-v1.0-mc-grch38.vcf.gz`) by decomposing nested variants into single-ID records using the PanGenie conversion script `convert-to-biallelic.py` as documented in the associated Zenodo record description link.

Table S15: Step-wise computational costs for each downstream workflow. Times are wall-clock minutes; memory is reported as peak resident set size (RSS) in gigabytes; CPU time is user + system seconds. All workflows were executed using 32 threads, except for the BWA-MEM indexing step which is single-threaded. Indexing steps that are sample-independent (e.g., BWA-MEM, Giraffe(linear), Giraffe(1kGP_pangenome), Giraffe(HPRC_pangenome), and Giraffe(diploid)) are reported here for completeness but are not included in the runtime plots, as these constitute one-time offline reference builds per dataset.

Pipeline	Step	Time (min)	Max RSS (GB)	CPU time (s)
BWA-MEM	Indexing	68.12	4.55	4,059.79
	Alignment & Lifting	166.12	10.12	315,563.56
Giraffe(linear)	Indexing	52.20	88.69	8,964.01
	Alignment & Lifting	145.60	54.26	280,694.18
Giraffe(1kGP_pangenome)	Indexing	1,513.92	401.54	749,299.52
	Alignment & Lifting	230.45	71.89	444,300.74
Giraffe(HPRC_pangenome)	Indexing	0.00	—	0
	Alignment & Lifting	245.37	52.63	472,920.17
Giraffe(Imputefirst_c1)	Indexing	92.88	126.57	20,779.55
	Alignment & Lifting	181.33	60.29	349,693.77
Giraffe(Imputefirst_c5)	Indexing	93.53	126.36	18,841.63
	Alignment & Lifting	182.09	60.38	352,221.29
Giraffe(Imputefirst_c20)	Indexing	92.27	126.46	18,730.61
	Alignment & Lifting	181.02	60.40	350,652.21
Giraffe(diploid)	Personalization/Indexing	87.56	125.16	37,632.47
	Alignment & Lifting	309.17	64.25	487,703.54
Leviosam2(Imputefirst_c1)	Indexing*	172.71	4.35	10,165.95
	Alignment & Lifting	1521.56	37.06	1,476,738.58
Leviosam2(Imputefirst_c5)	Indexing*	177.23	4.35	10,394.66
	Alignment & Lifting	1455.60	37.06	1,364,192.97
Leviosam2(Imputefirst_c20)	Indexing*	177.23	4.35	10,424.89
	Alignment & Lifting	1441.91	37.06	1,365,111.68

* Leviosam2(Imputefirst_c*) Indexing step excludes T2T-CHM13 reference indexing, which is a one-time offline operation analogous to the sample-independent indexing steps of other workflows.

Table S16: Summary of external datasets used in this study. Each GIAB sample (HG001–HG005) is listed with its paired-end 30× Illumina Novaseq PCR-free fastq files. Fastqs, reference panels, truth sets, and other resources are provided as hyperlinks.

Category	Dataset (hyperlinks)
Sample fastqs	HG001 R1, HG001 R2
	HG002 R1, HG002 R2
	HG003 R1, HG003 R2
	HG004 R1, HG004 R2
	HG005 R1, HG005 R2
Reference panels	HGSVC2
	HGSVC3
	HPRC_filtered*
Truth sets	GIAB HG001 v4.2.1
	GIAB HG002 v4.2.1
	GIAB HG003 v4.2.1
	GIAB HG004 v4.2.1
	GIAB HG005 v4.2.1
	GIAB HG002 CMRG v1.0
	GIAB genome stratifications v3.1
	GIAB CMRG regions
	HG002 T2TQ100 v1.0
Other resources	Genetic maps (plink)
	1kGP Phase3 GRCh38 callset
	HPRC v1.1 default pangenome index (.gbz)
	HPRC v1.1 frequency-filtered index (.gbz)
	T2T CHM13 v2.0 FASTA

*HPRC_filtered panel uses the bi-allelic file `cactus_filtered_ids_biallelic.vcf.gz`, generated from `cactus_filtered_ids.vcf.gz` (derived from `hprc-v1.0-mc-grch38.vcf.gz`) by decomposing nested variants into single-ID records using the PanGenie conversion script `convert-to-biallelic.py` as documented in the associated Zenodo record description link.

Supplementary Figures

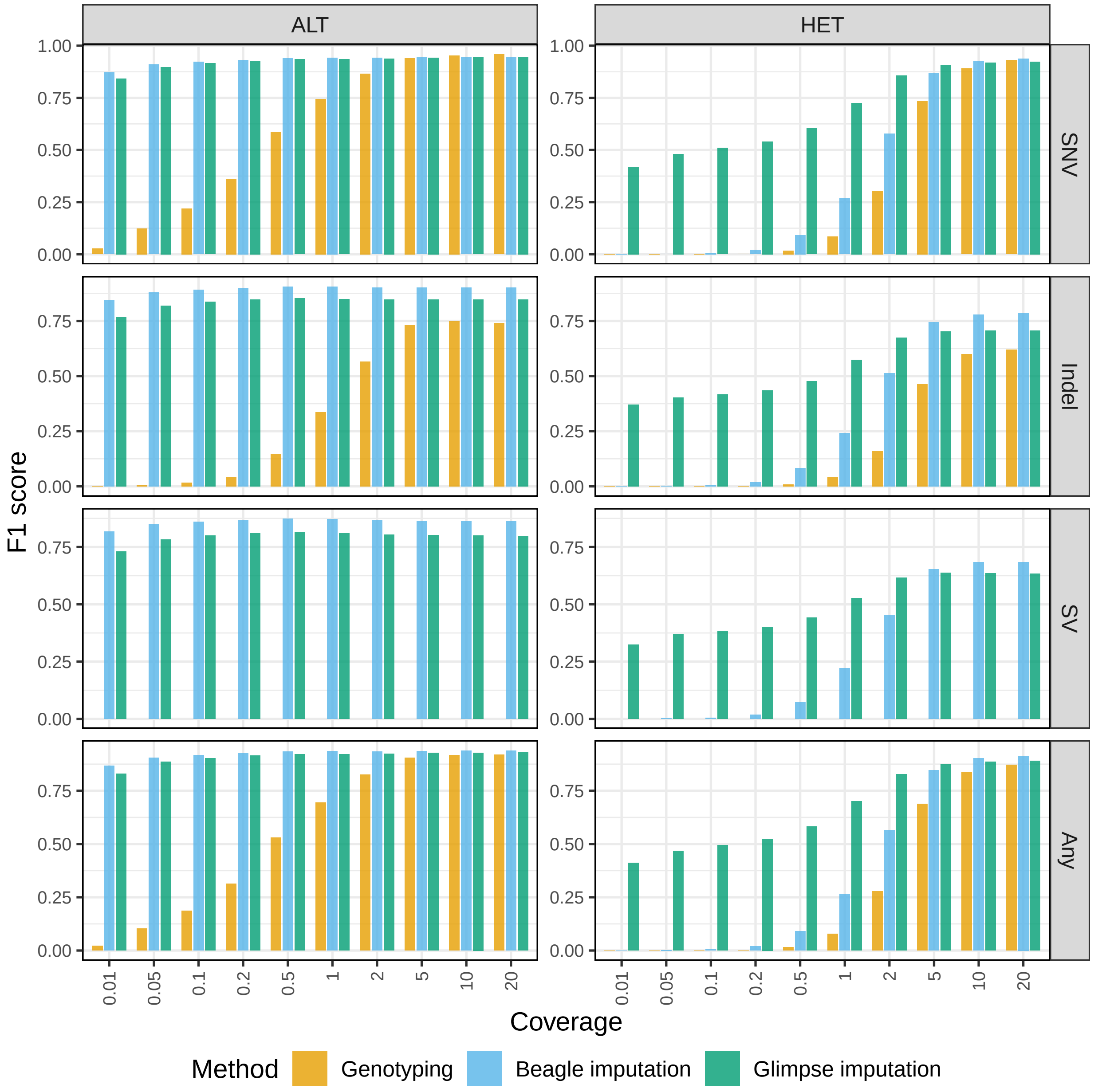

Figure S1: F1 score of Alternate allele calls (ALT) and heterozygous calls (HET), stratified by variant type, for the HG002 personalized genomes, imputed using HGSVC2 reference panel.

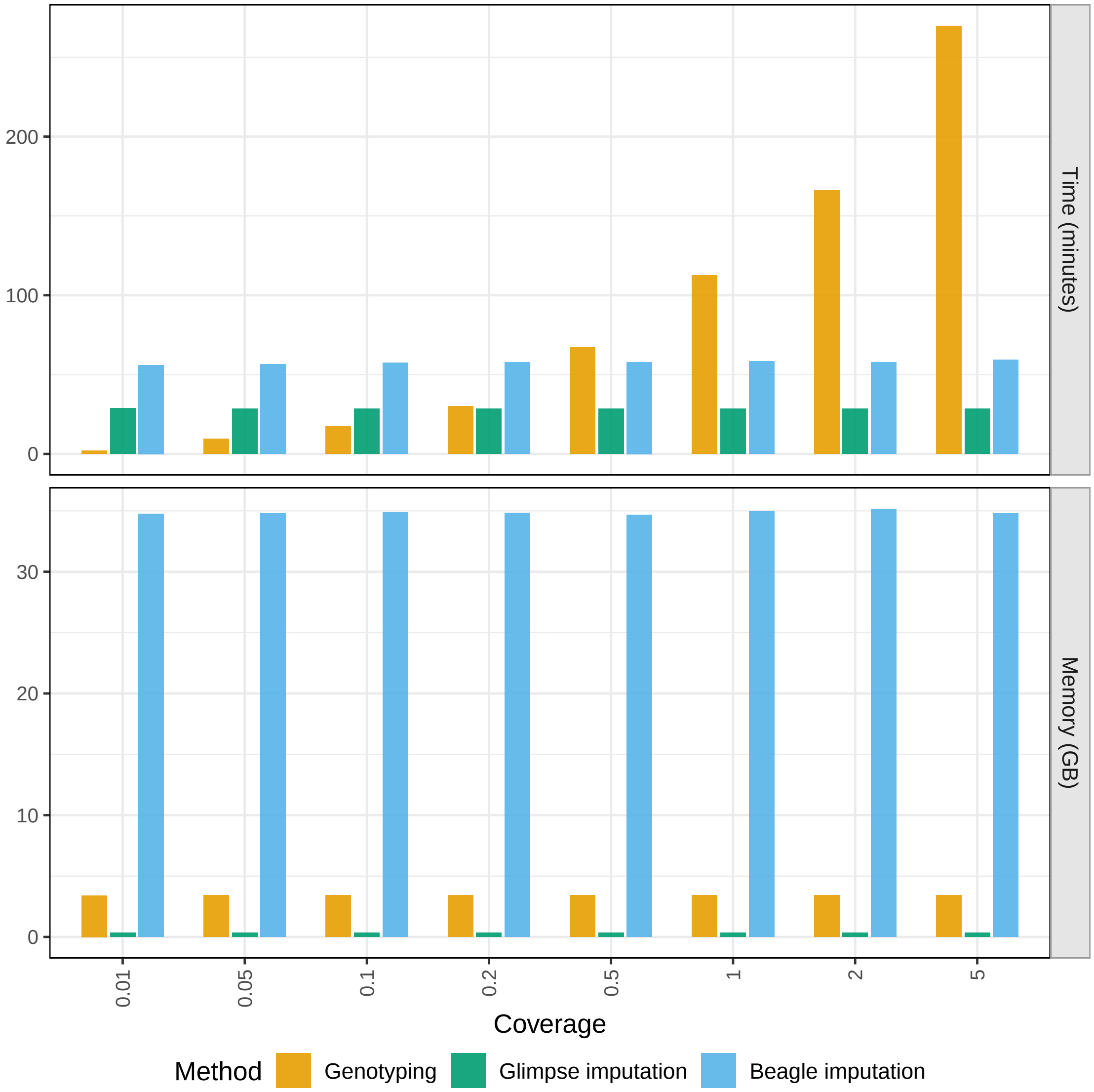

Figure S2: Computational overhead of genotyping (left) and imputation (middle: Glimpse, right: Beagle) in the personalization component of the impute-first workflow on HG002.

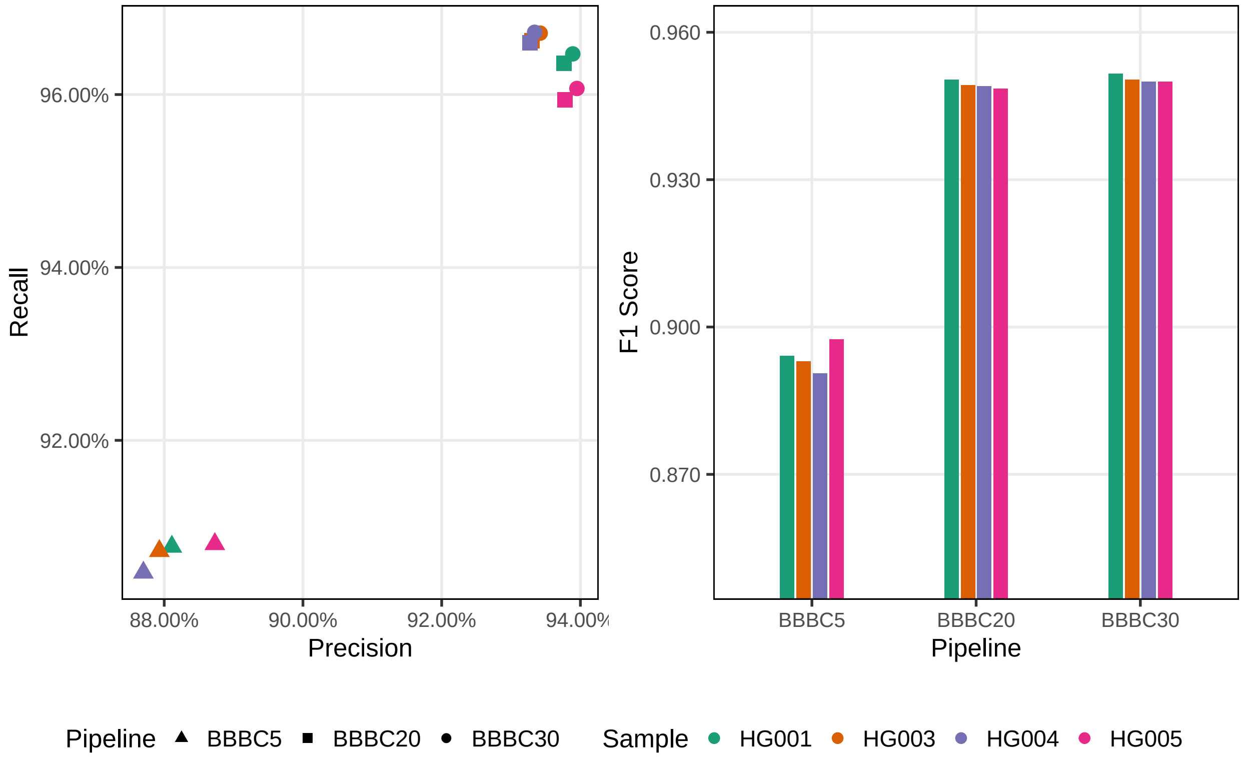

Figure S3: Accuracy comparison using the HGSVC3 reference panel on HG001, HG003, HG004, and HG005 samples. BBBC5, BBBC20, and BBBC30 denote the Bowtie 2(B)+BCFtools(B)+Beagle(B) pipeline evaluated at 5×, 20×, and 30× coverage (C), respectively.

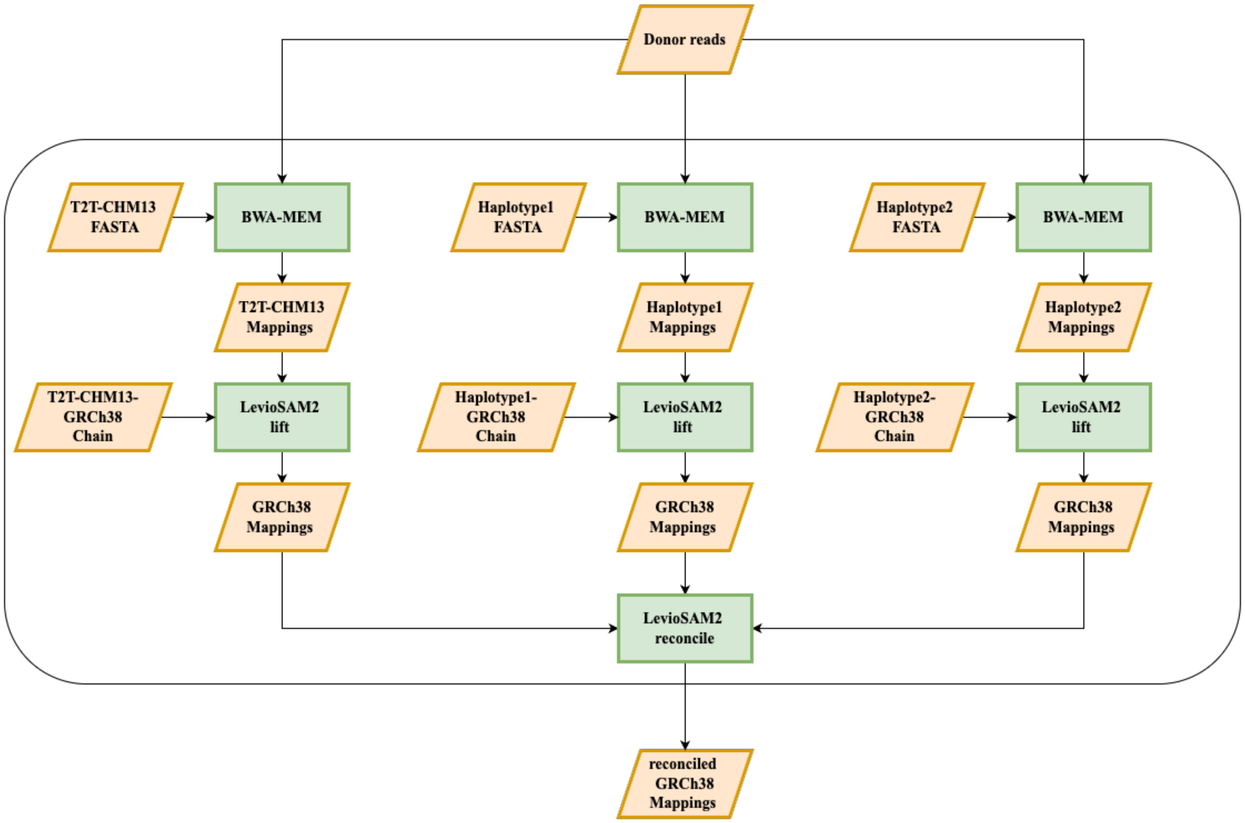

Figure S4: Workflow of alignment with LevioSAM2 + BWA-MEM. The three sub-pipelines include alignment to two personalized haplotypes and alignment to T2T-CHM13. These are run sequentially, and the resulting alignments are reconciled, merged, and lifted over to GRCh38 coordinates prior to evaluation.

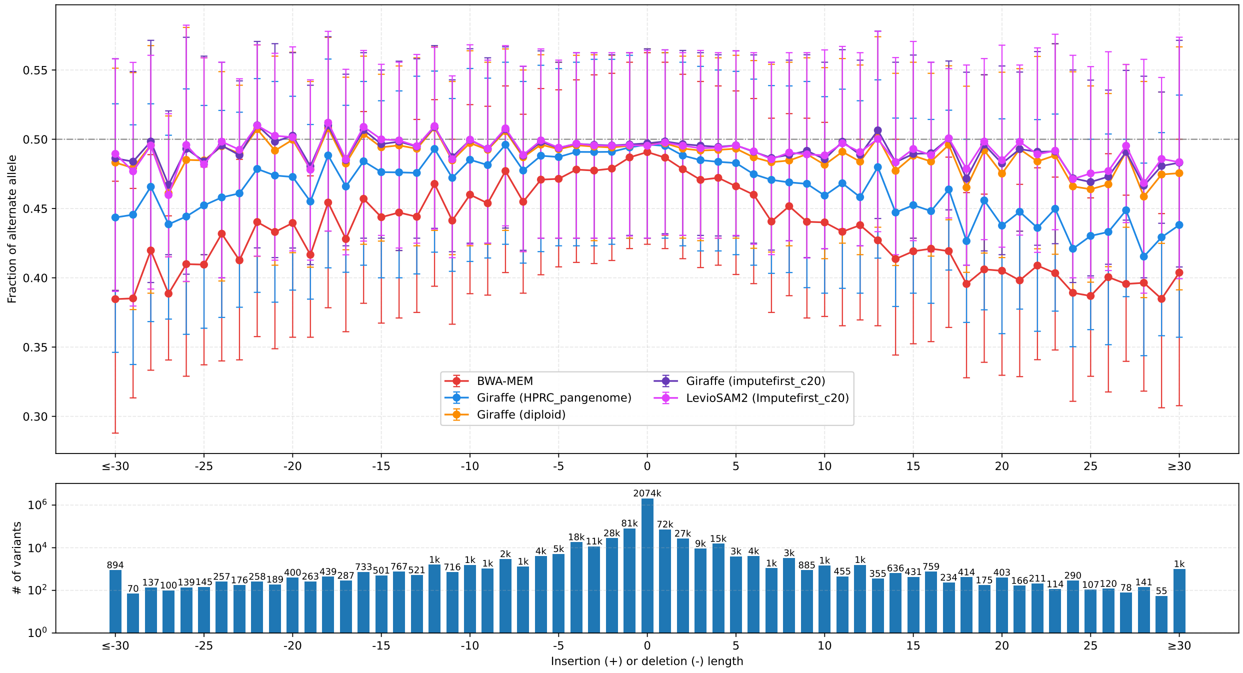

Figure S5: Bias-by-allele-length plots on HG003 donor reads generated for different workflows under analysis. Variants are stratified by length: positive values for insertions, negative for deletions, and zero for SNVs at HET sites across the genome.

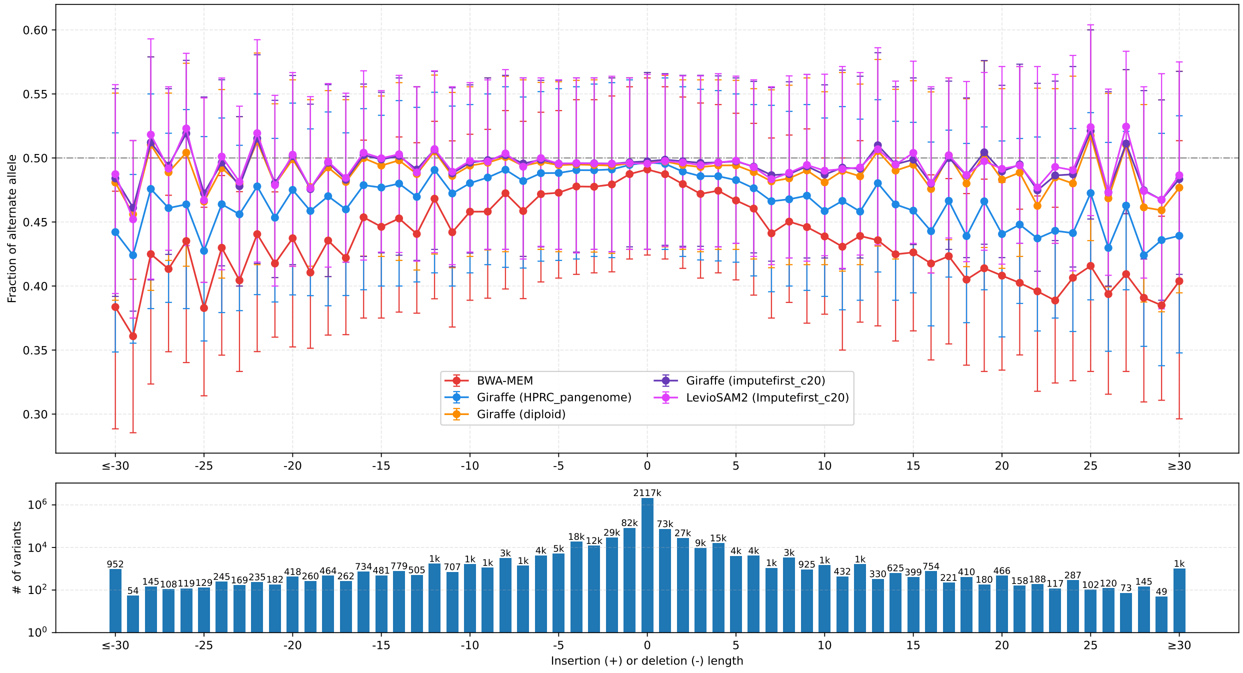

Figure S6: Bias-by-allele-length plots on HG004 donor reads generated for different workflows under analysis. Variants are stratified by length: positive values for insertions, negative for deletions, and zero for SNVs at HET sites across the genome.

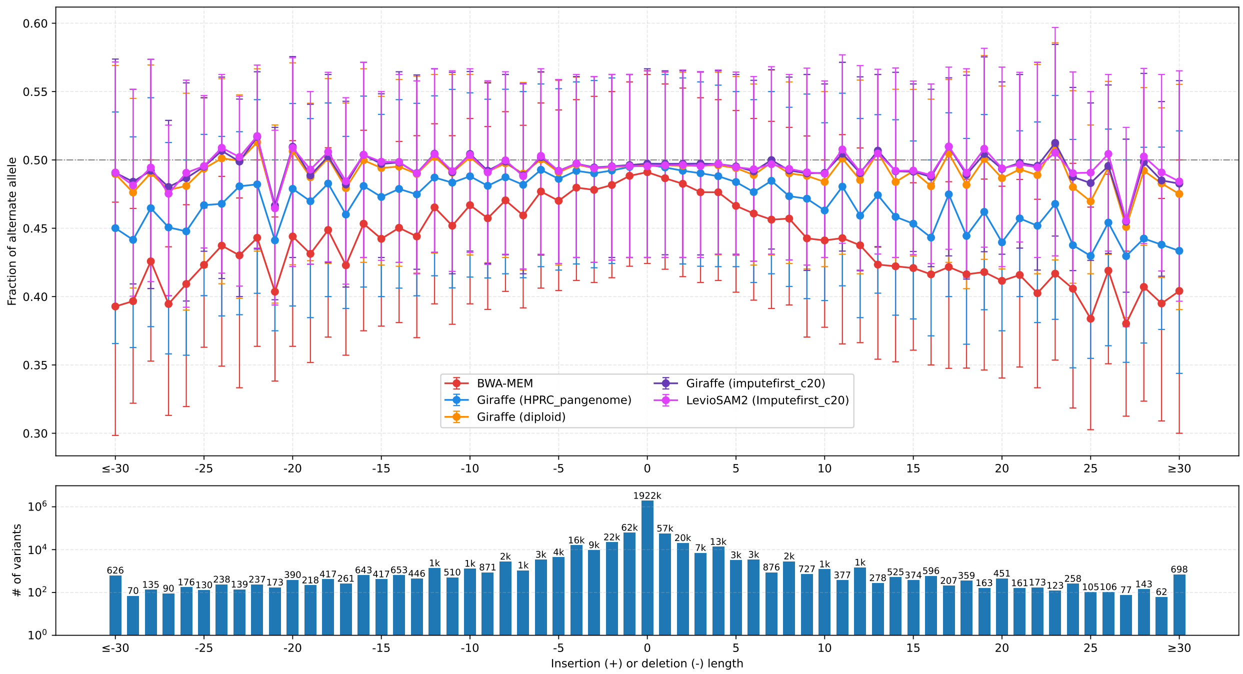

Figure S7: Bias-by-allele-length plots on HG005 donor reads generated for different workflows under analysis. Variants are stratified by length: positive values for insertions, negative for deletions, and zero for SNVs at HET sites across the genome.

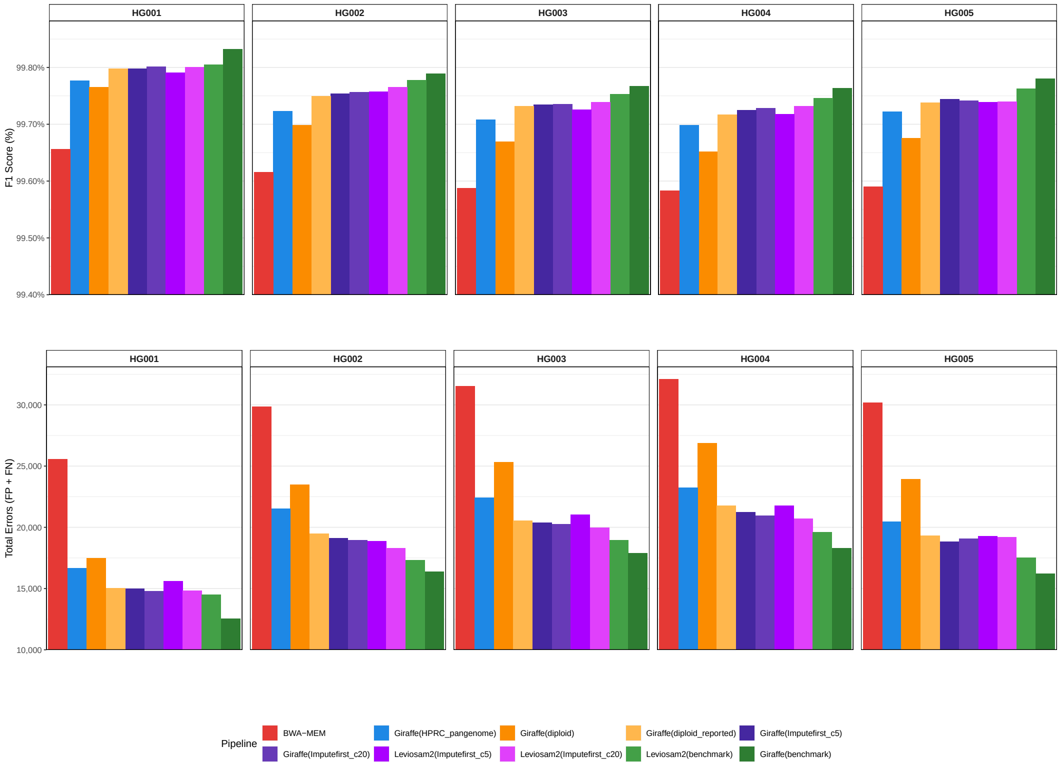

Figure S8: Overall Variant calling F1 score & Total error counts (sum of FP & FN counts) for HG001–HG005 samples. Variants were called with DeepVariant 1.5.

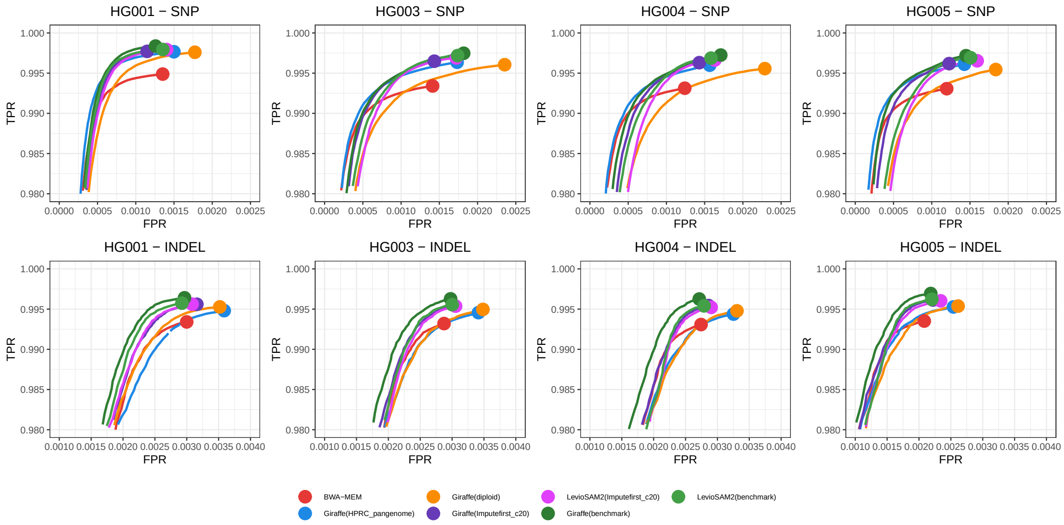

Figure S9: Hap.py ROC curves for samples HG001, HG003, HG004 and HG005

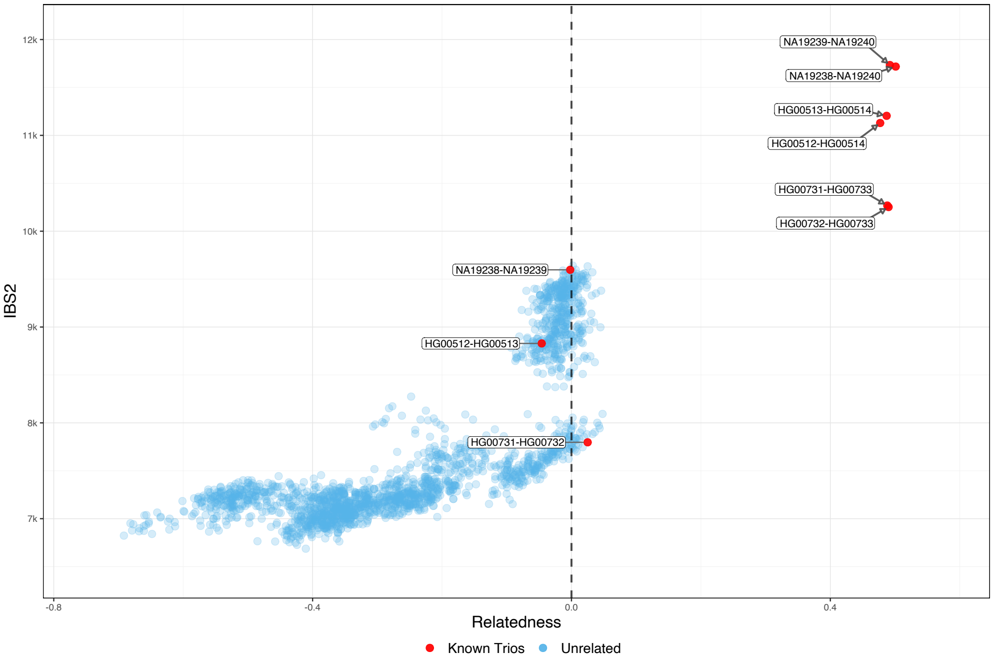

Figure S10: Somalier [67] relatedness analysis for HGSVC3 65 samples. X-axis: Relatedness score. Y-axis: IBS2 - shared genotype sites. Somalier analysis revealed that 99.7% of sample pairs (2,074/2,080) show no significant relatedness, while only 0.3% (6/2,080) represent known family relationships among the HGSVC3 samples. Known trios include: Yoruba trio (NA19238, NA19239, NA19240), Puerto Rican trio (HG00731, HG00732, HG00733), and Southern Han Chinese trio (HG00512, HG00513, HG00514). These familial relationships are documented and do not represent random relatedness that could inflate imputation accuracy. *Related sample pairs: HG00512–HG00514, HG00513–HG00514, HG00731–HG00733, HG00732–HG00733, NA19238–NA19240, NA19239–NA19240.*

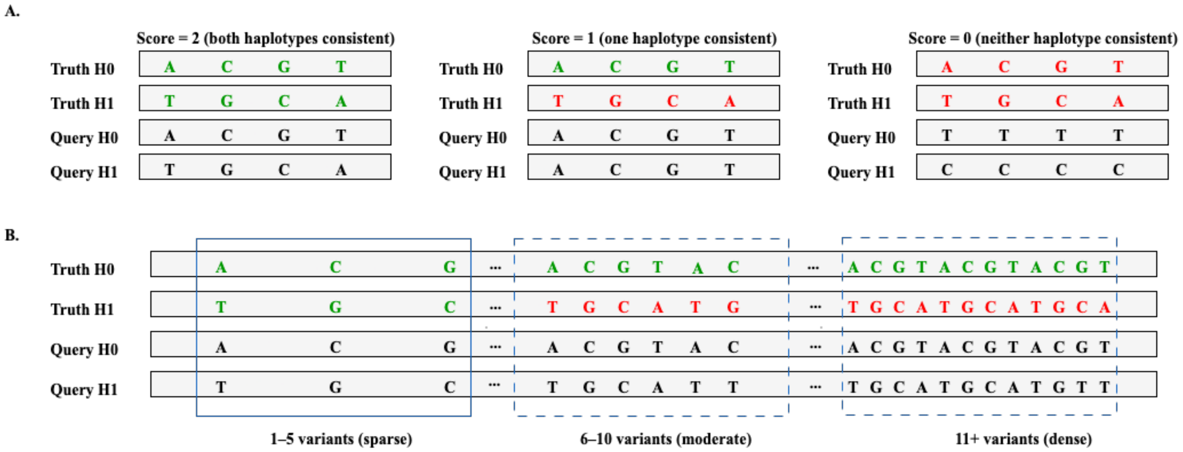

Figure S11: Illustration of the window accuracy metric. **Panel A:** For each variant site, we consider a 200 bp window beginning at that site and extending to the right. Within each window, true haplotypes (*Truth H0*, *Truth H1*) are compared to query haplotypes (*Query H0*, *Query H1*). A window is scored as 0, 1, or 2 depending on whether none, one, or both truth haplotypes exactly match a query haplotype at all sites. To account for phasing ambiguity, either query haplotype can correspond to either truth haplotype within the window, provided that the query haplotypes map to distinct truth haplotypes. The best matching assignment is used for scoring. **Panel B:** This approach enables quantification of phasing accuracy across contiguous genomic regions and allows comparison across windows stratified by variant density (1–5, 6–10, or 11+ variants per window). The rectangular blue boxes represent a few examples of 200 bp windows of varying densities. Note: Although this figure shows only SNVs, the approach works in a similar way for any variant type.
